# A systematic review of gut microbiota composition in observational studies of major depressive disorder, bipolar disorder and schizophrenia

**DOI:** 10.1038/s41380-022-01456-3

**Published:** 2022-02-22

**Authors:** A. J. McGuinness, J. A. Davis, S. L. Dawson, A. Loughman, F. Collier, M. O’Hely, C. A. Simpson, J. Green, W. Marx, C. Hair, G. Guest, M. Mohebbi, M. Berk, D. Stupart, D. Watters, F. N. Jacka

**Affiliations:** 1 The Institute for Mental and Physical Health and Clinical Translation (IMPACT), Food & Mood Centre, School of Medicine and Barwon Health, Deakin University, Geelong, VIC Australia; 2 Murdoch Children’s Research Institute, Parkville, VIC Australia; 3 Melbourne School of Psychological Sciences, Faculty of Medicine, Dentistry and Health Sciences, The University of Melbourne, Melbourne, VIC Australia; 4 Melbourne Neuropsychiatry Centre, Department of Medicine, Faculty of Medicine, Dentistry and Health Sciences, The University of Melbourne and Melbourne Health, Melbourne, VIC Australia; 5 Monash Alfred Psychiatry Research Centre (MAPcr), Central Clinical School, Faculty of Medicine, Nursing and Health Sciences, Monash University, Parkville, VIC Australia; 6 Department of Psychiatry, Peninsula Health, Frankston, VIC Australia; 7 Deakin University, School of Medicine, Geelong, VIC Australia; 8 Department of Gastroenterology, Barwon Health, Geelong, VIC Australia; 9 Department of Surgery, University Hospital Geelong, Barwon Health, Geelong, VIC Australia; 10 Biostatistics Unit, Faculty of Health, Deakin University, Melbourne, VIC Australia; 11 Orygen, The National Centre of Excellence in Youth Mental Health, Centre for Youth Mental Health, Florey Institute for Neuroscience and Mental Health and the Department of Psychiatry, The University of Melbourne, Melbourne, Australia; 12 Centre for Adolescent Health, Murdoch Children’s Research Institute, Melbourne, VIC Australia; 13 Black Dog Institute, Sydney, NSW Australia; 14 College of Public Health, Medical & Veterinary Sciences, James Cook University, Townsville, QLD Australia

**Keywords:** Depression, Bipolar disorder, Schizophrenia

## Abstract

The emerging understanding of gut microbiota as ‘metabolic machinery’ influencing many aspects of physiology has gained substantial attention in the field of psychiatry. This is largely due to the many overlapping pathophysiological mechanisms associated with both the potential functionality of the gut microbiota and the biological mechanisms thought to be underpinning mental disorders. In this systematic review, we synthesised the current literature investigating differences in gut microbiota composition in people with the major psychiatric disorders, major depressive disorder (MDD), bipolar disorder (BD) and schizophrenia (SZ), compared to ‘healthy’ controls. We also explored gut microbiota composition across disorders in an attempt to elucidate potential commonalities in the microbial signatures associated with these mental disorders. Following the PRISMA guidelines, databases were searched from inception through to December 2021. We identified 44 studies (including a total of 2510 psychiatric cases and 2407 controls) that met inclusion criteria, of which 24 investigated gut microbiota composition in MDD, seven investigated gut microbiota composition in BD, and 15 investigated gut microbiota composition in SZ. Our syntheses provide no strong evidence for a difference in the number or distribution (α-diversity) of bacteria in those with a mental disorder compared to controls. However, studies were relatively consistent in reporting differences in overall community composition (β-diversity) in people with and without mental disorders. Our syntheses also identified specific bacterial taxa commonly associated with mental disorders, including lower levels of bacterial genera that produce short-chain fatty acids (e.g. butyrate), higher levels of lactic acid-producing bacteria, and higher levels of bacteria associated with glutamate and GABA metabolism. We also observed substantial heterogeneity across studies with regards to methodologies and reporting. Further prospective and experimental research using new tools and robust guidelines hold promise for improving our understanding of the role of the gut microbiota in mental and brain health and the development of interventions based on modification of gut microbiota.

## Introduction

Gut microbiota—the symbiotic bacteria that live within our gastrointestinal (GI) system—act as ‘metabolic machinery’. They influence many aspects of physiology via neural, hormonal and immunological pathways [1, 2], so much so that some describe the gut microbiome as a ‘virtual organ’ [3, 4]. The interaction of the gut microbiota and central nervous system (CNS) is referred to as the ‘microbiota–gut–brain axis’ [5]. Although gut bacteria assist with the maintenance of health, they can also disrupt homeostatic regulation and may influence the aetiology and pathophysiology of many diseases, including mental disorders [6].

Whilst mental disorders have fundamentally been considered diseases of the CNS, our peripheral nervous system includes a highly innervated neural network dedicated to facilitating CNS communication with the gut [7, 8]: the enteric nervous system (ENS), which is often referred to as our ‘second brain’. Gut symptomatology and mental health are closely linked; indeed, gut symptoms have been identified as the most common somatic symptoms associated with depression [9], and anxiety disorders the most common psychiatric comorbidity in functional GI disorder patients [10]. Moreover, top-down treatments using antidepressants and psychological therapies have been effective in the treatment of irritable bowel syndrome (IBS) [11], further supporting mental disorders as not merely CNS disorders, but disorders with highly complex systemic interconnections [12, 13].

The field of psychiatry is somewhat unique in medicine in that the aetiology of mental disorders is largely unclear, and there are no robust biomarkers to aid in diagnosis or prognosis. This means that the differentiation between major mental disorders, such as mood and psychotic disorders, relies primarily on symptom presentation [14]. Given the rapidly growing evidence base for the gut microbiota’s influence on multiple systems and pathways that are known to be commonly dysregulated across these mental disorders, including inflammation [15, 16] and oxidative stress [17]; tryptophan metabolism and the kynurenine pathway [18, 19]; mitochondrial dysfunction [20]; neurotransmitters [21–25]; brain plasticity and neurotrophic factors [26] and metabolic processes [27, 28], the gut and its resident bacteria are increasingly recognised as important research targets. Critically, the functional potential of different bacteria is increasingly understood [29, 30], meaning that identification of key taxa that are differentially abundant in people with mental disorders and that influence these commonly dysregulated systems is an imperative. Such identification may afford opportunities for both understanding aetiology and identifying clinically useful biomarkers, as well as new targeted treatment strategies, including dietary changes [31–35], antibiotics [36–42], probiotic supplements [43–49] and even faecal microbial transplants [50–52].

Multiple observational studies have now investigated differences in gut microbiota composition in people with mental disorders compared to controls. Previous systematic reviews have synthesised these findings in depression [53–59], psychosis [59–62] and bipolar disorder (BD) [59–63]. These identified significant heterogeneity in study design and results across studies. Since these reviews, there have been a substantial number of new studies published in this field. This reflects the rapid expansion of gut microbiota research, and thus warrants an updated synthesis. However, previous reviews have rarely assessed gut microbiota composition across multiple mental disorders, especially with an aim of synthesising the evidence to identify commonalities or differences across disorders. Having a clear understanding of what bacteria may be commonly differentially abundant across disorders, as well as those that may discriminate between disorders, may afford clinically relevant information regarding aetiology, potential diagnostic and prognostic biomarkers and new treatment targets and strategies to change the gut microbiota in these major psychiatric disorders.

Therefore, we conducted the most up-to-date systematic review of the observational literature, identifying 11 additional studies since the last published review [59] comparing gut microbiota composition in participants with major depressive disorder (MDD), BD and schizophrenia (SZ) to controls. The aim of this systematic review was to synthesise the results of studies assessing possible differences in gut microbiota diversity and taxonomy between participants with mental disorders and controls for each disorder, and to identify any concordance in compositional differences across disorders. We additionally aimed to consider the potential functional significance of any identified compositional differences in relation to underlying pathophysiological processes involved in these serious mental disorders.

## Methods

### Protocol and registration

This systematic review adheres to the relevant criteria of the Preferred Reporting Items for Systematic reviews and Meta-analyses (PRISMA) statement [64] and was registered on PROSPERO (#CRD42020189823).

### Eligibility criteria

The research question and inclusion and exclusion criteria were determined a priori and developed using a PICOS structure (Patient, Intervention/Exposure, Comparators, Outcome, Study Design). Only peer-reviewed, full-text studies published in English were included. Inclusion criteria were: (1) observational study designs including cross-sectional, case-control, and prospective and retrospective cohort studies or intervention studies with baseline data; (2) adults aged 18 years or older; (3) participants with a clinical diagnosis of MDD, BD or SZ as the outcome variable compared to non-psychiatric controls; and (4) reporting of gut microbiota composition data as an exposure, for example measures of specific bacterial taxa, diversity and ordination techniques. We grouped studies by disorder when comparing cases to controls, and then we compared gut microbiota results across the pre-specified mental disorders.

### Information sources and search strategy

We conducted a systematic search using PubMed, EMBASE, CINAHL, CENTRAL and PsycINFO for articles published from database inception through to 3rd December 2021 using the search strategy (microbiome OR microbiota) AND (depression OR depressive OR schizophrenia OR psychosis OR bipolar OR mania OR manic OR “severe mental illness”).

### Study selection and data extraction

Primary screening was conducted independently by two reviewers (AJM, JD) using the web application Rayyan [65]. Full-text secondary screening was also performed in duplicate (AJM, JD) to assess eligibility and exclude studies that did not meet inclusion criteria. Reference lists of relevant publications were examined for studies not identified in the database search. Two reviewers independently extracted the data from eligible studies (AJM, JD). Where there were conflicts, consensus was achieved through discussion.

### Data items

Extracted aggregated data included: publication information; study design; participant demographics and characteristics; covariates and potential confounding variables; psychiatric disorder information and severity; blood biomarkers; gut microbiota data collection, sequencing and analysis methods; and gut microbiota outcome data (e.g. diversity, specific taxa, ordination).

### Synthesis

We were interested in associations between gut microbiota and psychiatric outcomes rather than predictive performance. Hence, where studies used a discovery dataset to determine gut microbiota composition in cases then tested its diagnostic performance for predicting the mental disorder on a validation dataset, we extracted the results from the discovery set only.

We included studies that used 16S rRNA gene sequencing (hereafter 16S) and whole-genome shotgun metagenomic sequencing (hereafter metagenomics), which aim to profile the entire faecal microbiota rather than focusing on specific taxa of interest (e.g. polymerase chain reaction, culture-based methods). 16S profiles faecal bacteria based on variations in their 16S rRNA gene, which is an important housekeeping gene present in all bacteria. Metagenomics sequences all the genetic material in the faecal sample and generates greater sequencing depth and resolution. Where studies reported differing results for both 16S and metagenomics, both sets of results were included.

Alpha-diversity (α-diversity) is a measure of gut microbiota diversity within a single sample, and its metrics are single numbers that describe the gut microbiota environment based on the number (i.e. richness) and/or distribution (i.e. evenness) of bacterial species present within an individual sample [66]. Studies in this review investigated whether people with a mental disorder had a higher or lower number or distribution of bacteria (α-diversity) compared to controls. Beta-diversity (β-diversity) derives from pairwise measures of similarity or dissimilarity in gut microbiota communities between groups. Ordination plots are used to display β-diversity statistics for visual inspection of data and allow researchers to observe whether samples from different groups cluster together or separately, suggesting compositional divergence of the gut microbiota between groups [67]. Studies included here investigated whether people with or without a mental disorder had a different gut microbiota composition from each other, as indicated by a between-group difference in β-diversity, and/or observed differences in visual clustering.

Bacteria are categorised into taxa based on their traits, such as their phylogeny, common metabolic potential, preferred growth environments, morphology and their genetic sequence. The broadest level of classification is called a ‘phylum’. Bacteria are progressively classified together into ranked groups called class, order, family, genus and species, in order of increasing similarity. Studies in this review identified taxa at various ranks that were different in their abundances in the gut microbiota of those with a mental disorder compared to controls. Some studies also identified bacteria that were best able to discriminate between those with and without mental disorders. For this review, we included both differentially abundant and discriminatory taxa in the taxonomy synthesis.

An aim of this review was to identify consistencies in gut microbiota across MDD, BD and SZ. Therefore, we identified and reported taxa that were reported as differentially abundant or discriminatory in at least 20% of the studies that reported data at that taxonomic rank. This allowed us to report taxa present in at least seven studies at phylum level, two studies at class level, three studies at the order level, seven studies at family level and nine studies at genus level. Due to the low resolution at the species level afforded by 16S, only studies using metagenomics were included in the species-level synthesis. Due to the heterogeneity and incomparability of processing and sequencing methodology, a meta-analysis was not conducted.

### Quality assessment

We used the National Institutes of Health (NIH) National Health, Lung and Blood Institute Study Quality Assessment Tool for Observational Cohort and Cross-sectional Studies [68] to assess the internal validity and potential bias of included studies. Study quality was rated as ‘Good’, ‘Fair’ or ‘Poor’ by two reviewers (AJM, CS), with discrepancies addressed through discussion. We considered body mass index, diet, anti-depressant use, or similar psychotropic medication use as the key confounding variables used in the quality assessment.

## Results

### Study selection

**Fig. 1:**
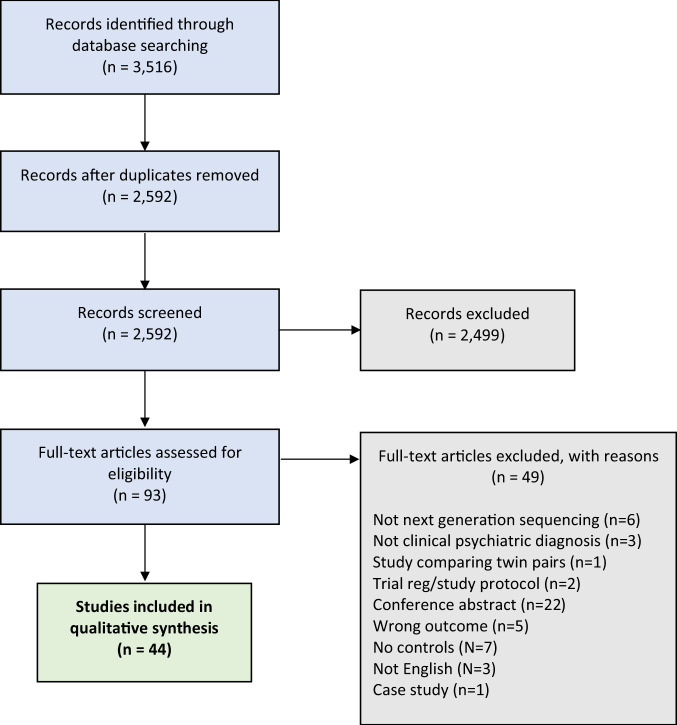
PRISMA flowchart. PRISMA flowchart and decision-making process for the inclusion of studies.

Our database search yielded a total of 3516 potentially relevant studies, with 2591 studies remaining after duplicates were removed. After primary screening, 106 studies were selected for full-text review (Fig. 1). Of the 44 studies that met criteria for inclusion in this systematic review (including a total of 2510 psychiatric cases and 2407 controls), 24 investigated gut microbiota composition in MDD (comprising data on 1038 MDD cases and 1048 non-MDD controls [69–92]), seven investigated BD (comprising data on 527 BD cases and 477 non-BD controls [81, 83, 93–97]) and 15 investigated SZ (comprising data on 945 SZ cases and 882 non-SZ controls [98–112]). Full summaries of study characteristics, results, covariates and methodologies are provided in Supplementary Tables S1–S8. Some studies conducted analyses across subgroups; this resulted in a total of 56 comparison groups across studies that, for simplicity, will be referred to as individual studies when reporting the results.

### Overview: major depressive disorder (MDD)

#### MDD study characteristics

The 24 eligible studies examining the gut microbiota in MDD were published between 2014 and 2021, with sample sizes ranging from 20 to 293 participants, and 75% conducted in China (*N* = 18/24 studies) (Table S1). Four studies included subgroups of the data. Subgroups included participants either aged 18–29 years or 30–59 years [69], males or females [70], active MDD cases or those considered in remission (R-MDD) [73] and MDD patients at baseline then after antidepressant treatment [89]. Results for these subgroups were extracted separately and compared to their control group. Therefore, 24 MDD studies including a total of 28 comparison groups were included in this systematic review.

#### Definitions of MDD cases & controls

Cases of MDD were mainly diagnosed using the DSM-IV criteria (46%; *N* = 11/24 studies), with versions of the Hamilton Depression Rating Scale (71%; *N* = 17/24 studies) the most common symptom severity measure. Most studies included actively depressed participants based on pre-defined symptom severity scores (54%; *N* = 13/24 studies); however, other studies included first-episode and/or drug-naïve participants (22%; *N* = 4/18 studies), patients in symptomatic remission (6%; *N* = 1/18 studies), or only required a prior diagnosis of MDD irrespective of current state (33%; *N* = 6/18 studies).

Most of the included studies specified psychiatric co-morbidity as a case exclusion criterion (63%; *N* = 15/24 studies). Two studies included participants with other psychiatric conditions despite one of these studies defining the presence of any other mental disorder as an exclusion criterion [70, 74]. Another study excluded comorbid psychiatric disorders but reported high anxiety scale scores observed among cases [85].

The inclusion criteria for controls were highly heterogeneous; most stipulated the use of ‘healthy’ controls. However, one study used outpatients from a neurological unit with no identified disorder, but with diffuse symptoms that could be related to a neuropsychiatric disorder [80]. Most studies specified either current (54%; *N* = 13/24 studies) or lifetime (25%; *N* = 6/24 studies) mental illness as an exclusion criterion, and reported the exclusion of systemic illnesses in general, or varying lists of specific diseases and infections (83%; *N* = 20/24 studies). One study reported on physical conditions present in controls [74], and three studies did not report the exclusion of systemic illnesses [77, 80, 82].

### Overview: bipolar disorders (BD)

#### BD study characteristics

The seven studies that investigated gut microbiota in BD were published between 2017 and 2021, with sample sizes ranging from 46 to 340 participants, with over half conducted in China (57%; *N* = 4/7 studies) (Table S2). One study separated the sample into subgroups, comparing the BD case group to either healthy controls or to relatives without BD [93], and results for each of these groups were extracted separately. Therefore, seven BD studies including a total of eight comparison groups were included in this systematic review.

#### Definition of BD cases and controls

Cases of BD were most commonly diagnosed using the DSM-V criteria (71%; *N* = 5/7 studies), with the Young Mania Rating Scale the most common symptom severity measure (57%; *N* = 4/7 studies). Most studies reported a current depressive episode (57%; *N* = 4/7 studies), and five studies provided a breakdown by BD subtype, showing either a higher prevalence of BD type I (60%; *N* = 3/5 studies) or BD type II (40%; *N* = 2/5 studies).

Most studies specified psychiatric co-morbidity as an exclusion criterion (57%; *N* = 4/7 studies). Three studies did not specifically report the exclusion of other psychiatric conditions [93, 94, 97]. No studies reported the inclusion of participants with other psychiatric co-morbidities. All studies referenced the use of ‘healthy’ controls; however, one study also included a subgroup of first-degree relatives with BD [93]. Most studies specifically stated current (57%; *N* = 4/7 studies) or lifetime (43%; *N* = 3/7 studies) psychiatric illness as an exclusion criterion for controls; however, one study only referred to ‘unaffected’ controls [94]. Five studies excluded controls with other physical diseases, whilst the remaining two studies did not specify the exclusion of systemic illnesses [93, 94].

### Overview: schizophrenia (SZ)

#### SZ study characteristics

The 15 studies examining the gut microbiota in SZ were published between 2018 and 2021, with sample sizes ranging from 26 to 214 participants, again predominantly conducted in China (80%; *N* = 12/15 studies) (Table S3). Five studies included subgroups: first-episode or treated-SZ [99], acute-episode or in symptomatic remission [101, 112], treatment resistant or treatment responders [109] and baseline or after treatment [111]. Overall, 15 studies in SZ including a total of 20 comparison groups were included in this systematic review.

#### Definition of SZ cases and controls

Cases of SZ were most commonly diagnosed using the DSM-IV criteria (47%; *N* = 7/15 studies), with the Positive and Negative Syndrome Scale the most common symptom severity measure (73%; *N* = 11/15 studies). Case inclusion criteria were heterogeneous across studies, including first episode (33%; *N* = 5/15 studies), symptomatic (13%; *N* = 2/15 studies) and remitted/stable cases (40%; *N* = 6/15 studies), whereas some studies only required a SZ diagnosis (27%; *N* = 5/15 studies).

The majority of the included studies specified any other psychiatric co-morbidity as an exclusion criterion (80%; *N* = 12/15 studies). No studies reported the inclusion of participants with other psychiatric conditions. All studies referenced the use of ‘healthy’ (87%; *N* = 13/15 studies) or ‘normal’ (13%; *N* = 2/15 studies) controls. Most studies stated the exclusion of psychiatric disorders in controls (73%; *N* = 11/15 studies); however, one study only excluded alcohol or drug abuse in the past year [104], and three studies did not specify any detail [101–103]. In addition, most studies excluded controls with other physical or systemic illnesses (80%; *N* = 12/15 studies). However, three studies did not exclude some cardiometabolic conditions and reported their presence in the results [100, 107, 109].

### Study findings

For α-diversity metrics and differential abundances of taxa, most studies defined a *p* value of <0.05 for significance in the methods and thereafter stated that results were ‘significantly different’ without a specific *p* value. For β-diversity metrics, differences were most commonly determined using permutational multivariate analysis of variance, with a *p* value < 0.05 deemed as significant.

### Alpha-diversity

**Fig. 2:**
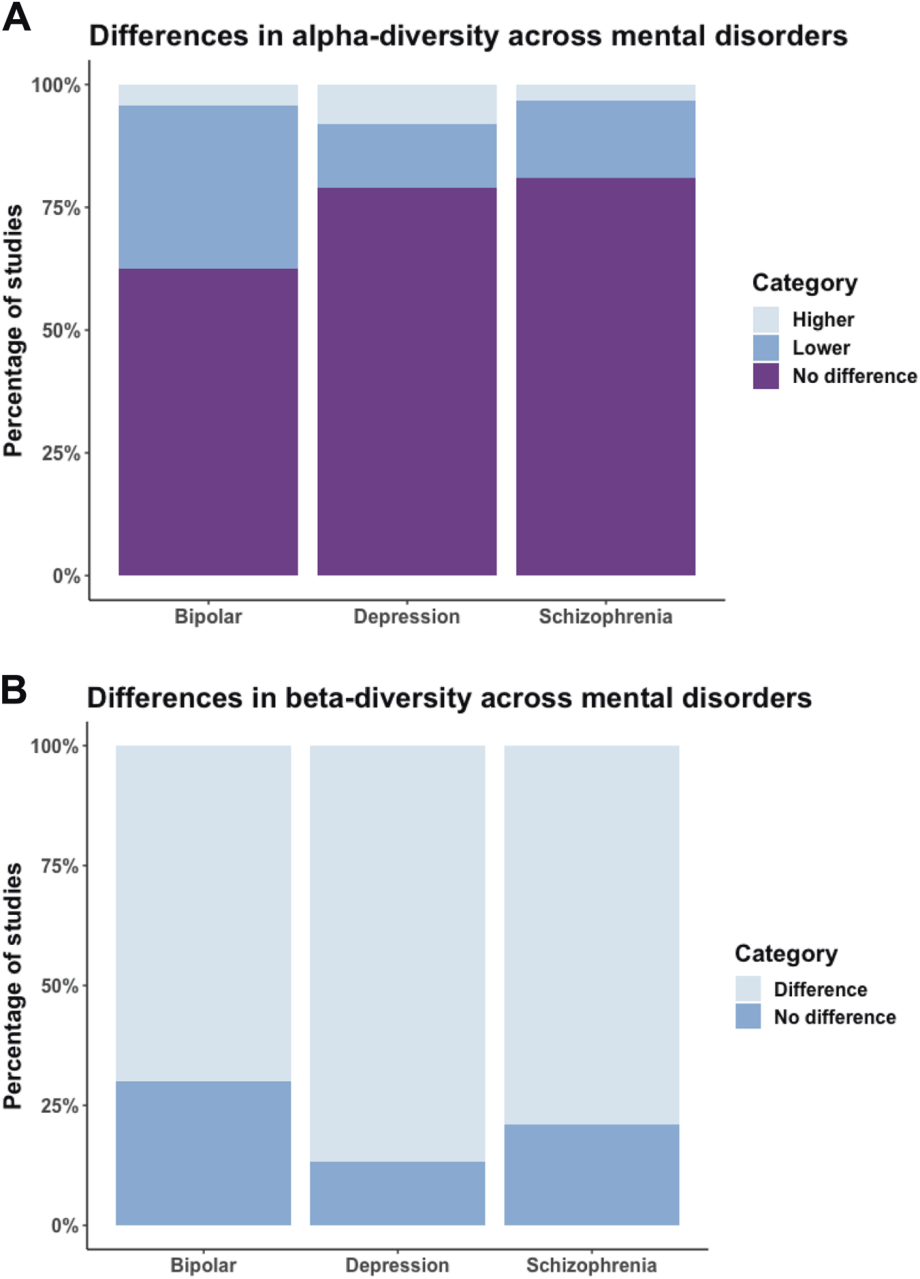
Differences in gut microbiota α- and β-diversity in cases compared to controls across studies of MDD, BD and SZ. **A** Testing of differences in α-diversity across comparison groups in MDD (*N* = 27), BD (*N* = 7), and SZ (*N* = 18). Purple indicates no reported difference in α-diversity, dark blue indicates lower α-diversity and light blue indicates higher α-diversity. **B** Testing of differences in β-diversity across comparison groups in MDD (*N* = 18), BD (*N* = 6) and SZ (*N* = 13). Light blue indicates reported differences in β-diversity, dark blue indicates no reported difference in β-diversity.

A total of 173 α-diversity analyses were conducted across 43 studies (Figs. 2A and S1). Reported metrics included measures of richness, phylogenetic diversity, evenness and composite metrics of evenness and richness. The most commonly reported α-diversity metric was the Shannon Index (27% of total analyses; *N* = 46/173).

The majority of analyses reported no difference in α-diversity between the psychiatric cases and controls (77% of total analyses; *N* = 134/173). Of the studies that did report a difference (*N* = 23%; 39/173 analyses), most reported lower α-diversity in cases (17% of total analyses; *N* = 29/173), and few reported higher α-diversity (6% of total analyses; *N* = 10/173).

In a breakdown of the separate mental disorders, there was no evidence of a difference in α-diversity for the majority of the MDD studies (79%; *N* = 68/86 analyses). However, a small number reported lower (13%; *N* = 11/86 analyses) or higher (8%; *N* = 7/86 analyses) α-diversity in MDD cases compared to controls. A similar pattern was observed for analyses in SZ, with no difference in most SZ studies (81%; *N* = 51/63 analyses); however, there was evidence of lower (16%; *N* = 10/63 analyses) or higher (3%; *N* = 2/63 analyses) α-diversity in a small number of studies. Whilst the results for BD were similar (63% showed no difference; *N* = 15/24 analyses), a greater proportion of studies reported lower alpha diversity compared to the other disorders (33%; *N* = 8/24 analyses) and only one study reported higher α-diversity (4%) (Fig. 2A).

### Beta-diversity and ordination plots

A total of 62 β-diversity analyses were reported across 37 studies (Figs. 2B and S2), most commonly using the unweighted UniFrac distance (34%; *N* = 21/62 analyses). However, nearly a third of these analyses (29%; *N* = 18/62 analyses) presented only the visual ordination plots and did not report any statistical testing.

Of the β-diversity analyses that were tested statistically (71% of total analyses; *N* = 44/62), most reported dissimilarity in the gut microbiota composition of cases compared to controls (80% of statistically tested analyses; *N* = 35/44 analyses). Differences in gut microbiota composition were reported in 87% of MDD β-diversity analyses (*N* = 13/15), 70% of BD analyses (*N* = 7/10) and 79% of SZ analyses (*N* = 15/19) (Fig. 2B). Of the studies that visually observed their data using ordination plots (52% of total studies; *N* = 32/62), most reported clear clustering of participants with a mental disorder separately to controls (59% of total ordination plots; *N* = 19/32).

### Taxonomy

Bacteria at various taxonomic ranks were reported. At the highest level, the phylum most commonly reported to be different between cases and controls was Bacteroidetes (*N* = 18/32 studies), although it was equally reported to be both higher (*N* = 10 studies) and lower (*N* = 12 studies) in abundance in cases (Fig. S3). Of the studies that reported their differential abundance, the phyla most frequently reported to have a higher abundance in psychiatric cases were Actinobacteria (*N* = 10/13 reported studies), Fusobacteria (*N* = 5/6 reported studies) and Proteobacteria (*N* = 10/12 reported studies), whereas Firmicutes was more frequently reported as lower (*N* = 12/19 reported studies).

Taxa at the class (*N* = 9 total studies) and order (*N* = 11 total studies) levels were infrequently reported across studies and few consistent patterns were observed. At the class level, studies reported higher abundances of Coriobacteriia (*N* = 3/9 reported studies) and Deltaproteobacteria (*N* = 3/9 reported studies), and lower abundances of Bacteroidia (*N* = 3/9 reported studies). At the order level, Actinomycetales was frequently reported as higher (*N* = 5/11 reported studies) and Bacteroidales lower (*N* = 4/11 reported studies) in cases compared to controls (Fig. S3).

At the family level, we identified ten families that were reported as being different between cases and controls (Fig. S3). The most commonly reported families were *Lachnospiraceae* (*N* = 21/36 reported studies) and *Ruminococcaceae* (*N* = 17/36 reported studies). Of the studies that reported their differential abundance, results for *Lachnospiraceae* were directionally heterogeneous, whereas *Ruminococcaceae* was predominantly lower in psychiatric cases (*N* = 13/17 reported studies). The bacterial family *Enterobacteriaceae* was consistently reported to be in higher abundance in mental disorders (*N* = 7/7 reported studies). No bacterial families were consistently reported as lower in cases.

Forty-eight studies identified differences in the abundances of taxa at the genus level, and we identified 21 bacterial genera that were consistently different in those with and without a mental disorder (Fig. S4). The most commonly reported genera observed to be differentially abundant in cases were *Bacteroides* (*N* = 20/48 studies), *Faecalibacterium* (*N* = 20/48 studies), *Prevotella* (*N* = 17/48 studies) and *Blautia* (*N* = 15/48 studies). Of the studies that reported on the following taxa, every study reported a higher abundance of *Eggerthella* (*N* = 12/12 reported studies), *Flavonifractor* (*N* = 9/9 reported studies) and *Veillonella* (*N* = 8/8 reported studies) in psychiatric cases compared to controls. No genus was always lower in cases; however, the majority of the studies that reported on *Faecalibacterium* (*N* = 17/20 reported studies), *Coprococcus* (*N* = 12/13 reported studies), *Haemophilus* (*N* = 8/9 reported studies) and *Ruminococcus* (*N* = 11/14 reported studies) observed lower abundances in psychiatric cases.

Differences in the abundances of the above genera were commonly reported across all three mental disorders, however some differences were more pronounced in individual disorders: higher *Alistipes* and *Parabacteroides* and lower *Prevotella* were observed in MDD; higher *Bifidobacterium* and *Oscillibacter* were observed in BD; and higher *Prevotella*, and lower *Bacteroides*, *Haemophilus*, and *Streptococcus*, were observed in SZ (Fig. S4).

Differences in the abundances of some genera were shared between disorders: higher *Escherichia*/*Shigella* and *Veillonella* were common to both MDD and SZ; higher *Megasphaera* and lower *Roseburia* were common to both SZ and BD; and higher *Enterococcus*, *Flavonifractor* and *Streptococcus* and lower *Faecalibacterium* and *Ruminococcus* were commonly observed in both BD and MDD. Finally, *Eggerthella* and *Lactobacillus* were frequently higher, and *Coprococcus* frequently lower, in all three mental disorders compared to controls (Fig. S4).

Twelve metagenomic studies reported differences at the species-level and we identified 18 species as consistently different between cases and controls (Fig. S5). Almost all reported species were higher in abundance in mental disorders (*N* = 16/18 reported species). However, *Haemophilus parainfluenzae* was more commonly reported as lower in mental disorders (*N* = 3/4 reported studies) compared with controls, and *Bacteroides helcogenes* was only reported as lower in mental disorders (*N* = 3/3 reported studies).

### Functional potential

Functional potential was measured in twelve studies of MDD using either the PICRUSt software [113] for 16S data (*N* = 8) or KEGG database mapping [114] for metagenomic data (*N* = 4). Very few pathways were reported in more than one study. One study only investigated pathways associated with tryptophan metabolism and biosynthesis, identifying differences between cases and controls [75]. Another study observed correlations between specific bacterial taxa and functional pathways within the KEGG orthologue database; however, they did not report what the specific pathways were, only providing the KEGG identification number [88]. Of the studies that examined multiple pathways and reported differences (*N* = 9), MDD was associated with an enrichment of: glycan biosynthesis and metabolism (33%; *N* = 3/9 reported studies) [86, 89]; lipopolysaccharide biosynthesis and biosynthesis proteins (33%; *N* = 3/9 reported studies) [72, 85]; and transport and catabolism pathways (33%; *N* = 3/9 reported studies) [86, 89], compared to controls. Conversely, cell motility and secretion (33%; *N* = 3/9 reported studies) [71, 89] and membrane transport (33%; *N* = 3/9 reported studies) [86, 89] were pathways enriched in controls compared to cases. One study did not observe any differences between MDD cases and controls across multiple investigated pathways [81].

Three studies of BD reported functional potential using either PICRUSt (*N* = 1) or KEGG (*N* = 2). The first study only investigated pathways associated with tryptophan metabolism and biosynthesis, finding differences between cases and controls [97]. The second study identified 31 pathways that were different between cases and controls; pathways associated with BD included tetrahydrofolate biosynthesis, pentose phosphate pathway and ornithine biosynthesis, whereas pathways associated with controls included peptides and nickel transport systems, branched chain amino acid transport system and putative sugar transport system [81]. The third study did not identify any pathways associated with either cases or controls across multiple investigated pathways [81]. Therefore, insufficient data were available to detect common functional pathways differentially reported across BD studies.

Six studies of SZ reported functional potential using PICRUSt (*N* = 5) or KEGG (*N* = 1). One of these studies reported differences across functional pathways relating to trimethylamine-N-oxide reductase, Kdo_2_-lipid A biosynthesis, and glycerol degradation to 1,2-propanediol [107]. Of the studies that reported on specific functional pathways (*N* = 4), those commonly enriched in SZ included: alpha-linolenic acid metabolism (50%; *N* = 2/4 reported studies) [101, 102]; ascorbate and aldarate metabolism (50%; *N* = 2/4 reported studies) [98, 102]; geraniol degradation (50%; *N* = 2/4 reported studies) [101, 102]; nucleotide metabolism (50%; *N* = 2/4 reported studies) [98, 102]; and pertussis (50%; *N* = 2/4 reported studies) [101, 102] pathways. On the other hand, pathways commonly enriched in controls included: phenylpropanoid biosynthesis (50%; *N* = 2/4 reported studies) [98, 102]; RNA transport (50%; *N* = 2/4 reported studies) [98, 102]; and starch and sucrose metabolism (50%; *N* = 2/4 reported studies) [98, 102] pathways. In addition, one study of SZ investigated gut brain modules (GBMs) [106]; GBMs are a framework developed to investigate microbial pathways believed to have neuroactive metabolic potential [115]. This study identified pathways associated with short-chain fatty acid (SCFA) synthesis (acetate, propionate, butyrate and isovaleric acid), tryptophan metabolism, and synthesis of neurotransmitters such as glutamate, GABA, and nitric oxide to be enriched in SZ cases compared to controls. One study did not report any differences in functional pathways between either first-episode or treated SZ cases compared to controls [99].

### Psychiatric symptom severity and gut microbiota composition

Of the studies that investigated associations between psychiatric symptom severity and differentially abundant taxa in MDD (39%; *N* = 11/28 MDD studies), very few taxa were commonly associated with depressive symptoms across multiple studies. Taxa belonging to *Blautia* (27%; *N* = 3/11 reported studies), *Parabacteroides* (18%; *N* = 2/11 reported studies) and *Ruminococcus* (18%; *N* = 2/11 reported studies) were reported as positively associated with depressive symptoms, whereas *Faecalibacterium* (36%; *N* = 4/11 reported studies), *Roseburia* (18%; *N* = 2/11 reported studies), and *Veillonella* (18%; *N* = 2/11 reported studies) were inversely associated with depressive symptoms (Table S4). Only two studies of MDD investigated associations between psychiatric symptom severity and diversity measures; one study observed no associations between depressive symptoms and measures of α- or β-diversity [79], whereas the other study reported contradictory associations between different measures of α-diversity and anxiety symptoms [81].

Few studies investigated associations between gut microbiota composition and psychiatric symptom severity in BD (38%; *N* = 3/8 BD studies). There were no commonalities in the results observed across BD studies, however one study reported the relative abundance of *Faecalibacterium* (33%; *N* = 1/3 reported studies) as inversely associated with depressive symptoms in BD patients, which was also observed in our synthesis of MDD. Ten studies examined associations between gut microbiota composition and symptom severity in SZ (45%; *N* = 10/22). Bacteria belonging to Firmicutes (20%; *N* = 2/10 reported studies), *Haemophilus* (20%; *N* = 2/10 reported studies), and *Lachnoclostridium* (20%; *N* = 2/10 reported studies) were positively correlated with SZ symptom severity, whereas *Bifidobacterium* (20%; *N* = 2/10 reported studies), *Coprococcus* (20%; *N* = 2/10 reported studies) and *Ruminococcaceae* (20%; *N* = 2/10 reported studies) were inversely associated with symptom severity (Table S4).

### Covariate data of relevance to the gut microbiota

#### Lifestyle behaviours

Smoking was the lifestyle factor most commonly reported or excluded across studies (52%; *N* = 23/44 studies). Dietary data were rarely collected or included as covariates (18%; *N* = 8/44 studies). Some studies reported certain diets as exclusion criteria, such as weight loss, high-fat, vegan, or completely vegetable-based diets or recent change in dietary habits (27%; *N* = 12/44 studies). Few studies collected data regarding sleep (9%; *N* = 4/44 studies) or physical activity (9%; *N* = 4/44 studies) (Table S5).

#### Use of probiotics, antibiotics, prebiotics and synbiotics

Most studies reported probiotic (59%; *N* = 26/44 studies) and/or antibiotic (77%; *N* = 34/44 studies) intake as an exclusion criterion, most commonly within the month prior to faecal sampling. Two of these studies also excluded participants with regular consumption of yogurt or milk fortified with probiotics [96] or probiotic-related drinks in the previous month [76]. Two studies reported participants’ use of antibiotics within the past year [100, 107]. The remaining studies did not report on probiotic (41%; *N* = 18/44 studies) and/or antibiotic (23%; *N* = 10/44 studies) intake. Less commonly excluded were use of prebiotic (34%; *N* = 15/44 studies) and/or synbiotic (20%; *N* = 9/44 studies) supplements. Eight studies (18%) did not report the intake, or exclusion of, any biotics (Table S5).

#### Psychotropic and other medication use

Most studies included cases who were taking psychotropic medications (59%; *N* = 26/44 studies); however, some studies included only drug-naïve (14%; *N* = 6/44 studies) or unmedicated (23%; *N* = 10/44 studies) participants or did not report psychotropic use (7%; *N* = 3/44 studies). Two thirds of studies did not report on, or exclude, non-psychotropic medication use (66%; *N* = 29/44 studies). The most common non-psychotropic medication exclusions were glucocorticoids, cytokines and biological agents (11%; *N* = 5/44 studies), as well as anti-diarrhoea or GI disorder medications (9%; *N* = 4/44 studies). Only three studies (7%) reported the use of non-psychotropic medications by participants [80, 96, 109] (Table S5).

#### Gastrointestinal co-morbidity and other medical conditions

Almost half of the included studies did not report on, or exclude, GI comorbidities (48%; *N* = 21/44 studies). The remaining studies reported GI-related exclusion criteria including IBS (11%; *N* = 5/44 studies), inflammatory bowel disease (IBD; 23%; *N* = 10/44 studies), GI surgeries (25%; *N* = 11/44 studies), GI symptoms (18%; *N* = 8/44 studies) and/or a GI illness/disease/disorder (20%; *N* = 9/44 studies). One study performed colonoscopies on all participants to confirm absence of organic colonic diseases [78].

Most studies excluded participants with chronic, metabolic or severe physical diseases (75%; *N* = 33/44 studies). Active bacterial, viral or fungal infections were also common exclusions (23%; *N* = 10/44 studies). Two studies of SZ reported on the proportion of participants with diabetes, hypertension and heart disease [100, 107] and did not exclude participants with these diseases due to their high co-morbidity with SZ. One study of MDD included participants with dyslipidaemia and hypertension but excluded those with arthritis and diabetes [74]. Nine studies (20%) did not report on physical co-morbidities or consider them as exclusion criteria (Table S5).

#### Biomarkers

Of the studies that collected additional biomarker data (27%; *N* = 12/44 studies), immune-related and inflammatory markers, such as interleukin 6 and C-reactive protein, were the most commonly reported (58%; *N* = 7/12 reporting studies). Five studies conducted metabolomic analyses: two studies in MDD found no differences in faecal metabolites [74] or neuroendocrine hormones [88]; two studies observed differences in multiple faecal metabolites in MDD cases compared to controls, mainly related to amino acid, nucleotide, carbohydrate, and lipid metabolism [86], and inflammatory pathways [87]; and a fifth study in SZ observed differences in tryptophan and kynurenine pathway metabolites [106] (Table S5).

#### Adjustments

Very few studies adjusted for covariates in their analyses (16%; *N* = 7/44); commonly considered variables included age, sex and/or BMI (Table S6). These studies adjusted when assessing α-diversity (5%; *N* = 2/44) and differential abundances of specific taxa (16%; *N* = 7/44), however only one study (2%) adjusted for covariates when addressing β-diversity.

#### Associations between gut microbiota composition and clinical covariates

Studies commonly explored multivariable associations (43%; *N* = 19/44) and/or bivariable associations (57%; *N* = 25/44) between gut microbiota composition and clinical covariates (Table S6). Of the studies that explored multivariable associations, most examined associations between clinical covariates and β-diversity (25%; *N* = 11/44) or differential abundances of bacteria (20%; *N* = 9/44), and only one study examined associations with α-diversity (2%; *N* = 1/44). The most common clinical covariates considered were sex and psychotropic medication-use, however most analyses did not report associations.

Of the studies that explored bivariable associations between gut microbiota composition and clinical covariates most examined associations with differential abundances of bacteria (48%; *N* = 21/44), whilst few examined associations with α-diversity (11%; *N* = 5/44) or β-diversity (5%; *N* = 2/44). The most investigated bivariable associations were between gut microbiota composition measures and self-reported psychiatric symptom scales.

#### Associations between gut microbiota composition and psychotropic treatment

Six studies with repeated measurements (follow-up) data observed changes in gut microbiota composition associated with treatment. Three studies were conducted in MDD: one study collected data at three time points across 30 days of escitalopram treatment [76] and did not observe clustering of MDD cases based on visit number using PCoA of weighted UniFrac distances, however they did observe changes in bacterial taxa across the three time points suggesting compositional changes associated with treatment; one study reported changes in gut microbiota composition after individualised treatment with escitalopram (maximum dose 20 milligrams per day) and found that gut microbiota composition was more similar to controls after treatment [89]; and the third study observed changes in gut microbiota composition after 4-weeks and 8-weeks of treatment with vortioxetine hydrobromide and observed a attenuation of the differences in α-diversity between cases and controls associated with treatment, and changes in the relative abundance of specific taxa after treatment [90]. One study, in BD, observed differences in the enrichment of specific bacterial taxa associated with 4-weeks of quetiapine treatment [95]. Two studies were conducted in SZ; one study observed a reduction in the number of bacteria that discriminated cases from controls after 3 months of antipsychotic treatment [106]; and one study observed an increase in α-diversity, changes in the relative abundance of *Lachnoclostridium* and *Romboutsia*, and the potential for basal levels of these taxa to predict treatment response, after 24-weeks treatment with risperidone [111]. In addition, one study in SZ compared first-episode drug-naïve SZ patients to chronic SZ patients who had been receiving antipsychotic treatment for at least 3 months [99], and one study compared treatment participants who were treatment resistant versus treatment responders, compared with healthy controls [109].

### Microbiome methods

#### Stool sample collection, transport and storage methods

Most studies collected and processed fresh faecal samples (67%; *N* = 28/44 studies). Other studies reported the use of stool sample collection and transport stabilisation kits (14%; *N* = 6/44 studies) or did not report on stool sample collection (23%; *N* = 10/44 studies). Duration and/or temperature of transportation was frequently not reported (55%; *N* = 24/44 studies) or samples were reported as immediately frozen (18%; *N* = 8/44 studies). Studies that did report transport reported cold-chain methods (11%; *N* = 5/44 studies). Most studies reported long-term storage at −70 or −80 °C (82%; *N* = 36/44 studies), with two of these studies reporting prior storage in home −20 °C freezers [80, 103]. One study only froze 21 of 64 faecal samples [74]. Seven studies (16%) did not mention storage methods (Table S7).

#### DNA extraction, sequencing and analysis methods

Most studies performed DNA extraction using commercially available DNA extraction kits (89%; *N* = 39/44 studies). Three studies did not use commercial kits [71, 79, 111], and three studies did not report the DNA extraction method [87, 91, 106]. Sequencing methods included 16S (86%; *N* = 38/44 studies) and metagenomics (16%; *N* = 7/44 studies), predominantly using the Illumina MiSeq system (55%; *N* = 24/44 studies). The 16S studies most commonly sequenced the V3–V4 hypervariable region (47%; *N* = 18/38 16S studies), and binned sequencing data into operational taxonomic units (OTUs;87%; *N* = 33/38 16S studies), often reporting a 97% similarity threshold (74%; *N* = 28/38 16S studies). Most studies taxonomically assigned sequences against the Ribosomal Database Project (37%; *N* = 14/38 16S studies) or SILVA (32%; *N* = 12/38 16S studies) mapping databases. Some studies explicitly report rarefying (20%; *N* = 9/44 studies) or normalising (11%; *N* = 5/44 studies) their gut microbiota data. Less than half of the included studies reported adjusting for multiple comparisons (45%; *N* = 20/44 studies) (Tables S7, S8).

### Quality assessment

Most studies were rated as ‘Fair’ (39%; *N* = 17/44 studies) or ‘Poor’ (43%; *N* = 19/44 studies) quality. The major sources of potential bias were lack of adequately specified and defined study populations, poor reporting of recruitment details and inclusion and exclusion criteria consistency, and lack of consideration of the pre-specified key potential confounding variables (Table S9). Only eight studies (18%) were categorised as ‘Good’ [73, 77, 86, 89, 90, 95, 106, 111], and only two studies (5%) clearly considered all of the key potential confounding variables [86, 106].

## Discussion

This is the largest systematic literature review to date of gut microbiota composition across the major psychiatric conditions MDD, BD and SZ, comprising 56 comparison groups across 44 studies, and a total of 2510 psychiatric cases and 2407 controls. Our syntheses provide no strong evidence for a difference in the number or distribution of gut bacteria (α-diversity) in those with, compared to those without, a mental disorder. However, we did observe consistent differences in the overall composition of the gut microbiota (β-diversity) between cases and controls within each mental disorder category. In addition, we identified specific bacterial taxa with differential abundances between cases and controls, some of which were observed to be consistently different from controls across all three mental disorders. We identified substantial heterogeneity across studies in methodologies and reporting, including differences in study population inclusion and exclusion criteria, methods of gut microbiota stool sample collection, storage, processing and analysis, and consideration of, or adjustment for, key variables known to be associated with gut microbiota composition. Finally, we conducted a quality assessment of the included studies, the results of which highlight the need for guidelines on the conduct and reporting of microbiome-related research.

### Measures of α-diversity are not useful indicators for mental disorders

Our syntheses provided no evidence for discrimination between mental disorder cases and healthy controls based on the richness and evenness (i.e. α-diversity) of their gut microbiota. Previously, higher α-diversity has been considered a marker of ‘better’ gut health. The assumption is that greater species number and diversity may increase gut ecosystem resilience and stability due to increased functional redundancy for metabolic functions, and a more robust resistance to pathogenic invasion [116, 117]. However, growing evidence from gut microbiota research in humans suggests that α-diversity metrics are of limited utility as a measure of gut health or to discriminate between disease cases and controls. This has been demonstrated in similar reviews of the gut microbiota’s associations with obesity [118], type 2 diabetes mellitus [119], IBS [120] and ulcerative colitis [121]. These reviews align with our syntheses, and are also concordant with recent findings in other neurological and psychiatric conditions such as Parkinson’s disease [122], multiple sclerosis [123], autism spectrum disorder [124] and anxiety, which also report equivocal α-diversity findings [56].

Our findings are further corroborated by a recent systematic literature review and meta-analysis of gut microbiota composition across psychiatric disorders [59]. Concordant with our observations, the authors reported no differences in α-diversity composite indices (i.e. Shannon Index, Simpson Index) or phylogenetic diversity across mental disorders. With regards to richness, meta-analyses identified a pooled reduction in richness; however, when looking at individual diagnoses this reduction was only observed in BD, which we also observed. To date, very few studies have investigated gut microbiota composition in BD compared to the other psychiatric disorders included in this review, and no studies have investigated state-associated compositions across different phases of this disorder. Additional research into the gut microbiota in BD is required in order to confirm and further elucidate the relevance of reduced richness in this mental disorder.

The assumption that lower α-diversity is a biomarker of a poorer gut ecosystem is problematic. For example, two individuals may have equivocal α-diversity of their gut microbiota, but one individual’s ecosystem may be abundant in taxa with potential pro-inflammatory metabolism and pathogenicity, whereas the other may be abundant in microbes associated with the production of beneficial metabolites. Despite similar α-diversity, the implications of these two gut microbiota compositions for host health may be completely different. The basic metrics of α-diversity are derived from ecology frameworks; therefore, their applicability to complex and dynamic human physiological systems may be inadequate. In this sense, α-diversity metrics may have greater utility in studies that employ a common intervention across participants, such as antibiotic or probiotic treatment strategies, where change in α-diversity may be a useful marker.

Seven of the included studies reported higher α-diversity associated with mental disorders. Of these studies, all but one was very recently published (2020/21) and used either the SILVA or RDP Classifier for taxonomic assignment for 16S sequencing. These two databases are iteratively updated to reflect advances in taxonomic discoveries. This is in comparison to the Greengenes database, which has not been updated since 2013. Despite this, Greengenes has been widely used for gut microbiota research for almost a decade, including for nine of the studies included in this review. It is possible that earlier studies, using Greengenes or less complete databases, may have been missing or misidentified many bacterial taxa, impacting on the accuracy of α-diversity measures as a reflection of gut microbiota richness and evenness. Moreover, one third of the studies that reported higher α-diversity used metagenomic sequencing. A recent study comparing taxonomic characterisation using 16S and metagenomics revealed that 16S was only able to detect a part of the gut microbiota community that was identified using metagenomics, and that metagenomics was superior for detecting low abundance, yet biologically relevant, taxa [125]. Estimates of α-diversity are also inherently biased by sampling depth, which is the number of sequences yielded per sample [126]. As sampling depth is rarely adjusted for in analyses, comparisons of α-diversity between studies are difficult to interpret [126]. In order to elucidate the variations in α-diversity that are possibly being obscured by the use of previously outdated databases and limitations of 16S, such as poorer sequencing depth and resolution, these results suggest that the use of α-diversity metrics should ensure they use the most recently updates databases or, ideally, use metagenomic sequencing, and that caution should be applied in using α-diversity as a proxy of host health.

### Measures of β-diversity suggest differences in overall community composition between cases and controls

On the other hand, β-diversity analyses consistently suggested that individuals within each mental disorder category harboured a more similar gut microbiota composition to each other than to the clustered compositions observed in ‘healthy’ controls. Our findings are somewhat discordant with those of several previous systematic reviews [53, 54, 56, 59, 61], which report less consistent differences in β-diversity; however, this may be due to the greater number of studies included in our review. Whilst β-diversity analyses can indicate whether differences exist between groups, they do not indicate what the differences are, or at which level of taxonomy they are occurring. Therefore, the causes of these divergences in gut microbiota composition, the clinical or functional meaning of these differences, or how these differences might reflect disruptions in physiological processes, all remain unclear. Whilst metagenomics can suggest functional potential of bacteria, further research combining multi-omics approaches, including meta-transcriptomics (to identify which genes are being expressed), proteomics (to identify which proteins are being formed), and metabolomics (to identify which metabolites are being produced), are likely to provide greater insight into functional differences and assist in understanding how these compositional differences are associated with pathological processes potentially influencing mental health.

### Taxa that are commonly differentially abundant across mental disorders compared to controls

**Fig. 3:**
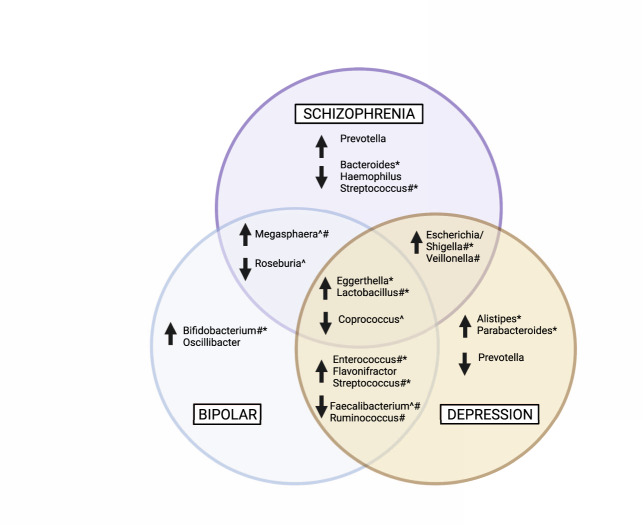
Bacterial genera that were commonly higher or lower in mental disorders compared to controls. Bacterial genera that were differentially abundant or discriminatory in equal to or >20% (*N* = 9) of the included studies that reported differences at the genus level were collated. The purple circle indicates genera that were different in SZ cases compared to controls, the blue circle indicates genera that were different in BD cases compared to controls, and the yellow circle indicates bacterial genera that were different in MDD cases compared to controls. Genera within overlapping sections indicate where differences in the abundance of that genus was a shared feature between two disorders. The centre overlap indicates genera that were commonly different in cases compared to controls across all three mental disorders. MDD Major depressive disorder, BD Bipolar disorder, SZ Schizophrenia, GABA γ-aminobutyric acid. ^ indicates butyrate-producing genera; # indicated lactic acid-producing and utilising genera; * indicates bacteria that influence glutamate and GABA metabolism. Created with BioRender.com.

Our review identified some bacterial genera that were commonly differentially abundant in cases compared to controls across all three mental disorders (Fig. 3); these were higher *Eggerthella* and *Lactobacillus*, and lower *Coprococcus*. In addition, some bacterial genera were overlapping between two disorders. Both MDD and SZ were also associated with higher *Escherichia*/*Shigella* and *Veillonella*, and SZ and BD shared similarities of higher *Megasphaera* and lower *Roseburia*. The two mood disorders, MDD and BD, had more commonalities, including higher *Enterococcus*, *Flavonifractor*, and *Streptococcus*, and lower *Faecalibacterium* and *Ruminococcus*. The potential functional relevance of these differentially abundant taxa is discussed below.

### Taxa differentially abundant in specific mental disorders

Whilst there were common and overlapping taxa differentially abundant between cases and controls across all three mental disorders, there were some taxa for which differential abundances were specific to each mental disorder (Fig. 3). MDD was often characterised by higher *Alistipes* and *Parabacteroides* and lower *Prevotella*; BD was often characterised by higher *Bifidobacterium* and *Oscillibacter*; and SZ was often characterised by higher *Prevotella* and lower *Bacteroides*, *Haemophilus*, and *Streptococcus*. Again, the potential relevance of these differentially abundant taxa is discussed below.

### Potential functional implications of differentially abundant taxa identified in this review

#### Evidence of enriched bacteria with the potential to produce and utilise lactic acid

**Fig. 4:**
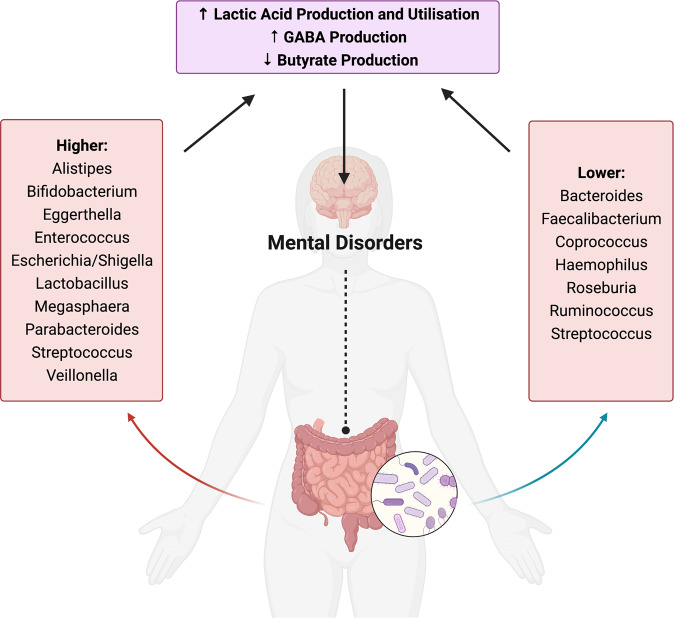
Potential functional implications of bacterial genera implicated as different in mental disorders in this review. Bacterial genera belonging to the human gut microbiota that were commonly different or discriminatory in people with mental disorders compared to controls. The functional potential of these bacteria to (1) produce and utilise lactic acid, (2) produce butyrate, and (3) influence the metabolism of glutamate and GABA, may be mechanistic pathways through which differences in these bacteria may influence their human host and contribute to mental disorder pathophysiology. Created with BioRender.com.

Our synthesis provided evidence of higher levels of lactic acid-producing bacteria across MDD, BD and SZ (Fig. 4). The genus *Lactobacillus* was higher in cases across all three of the major mental disorders. Similarly, higher abundances of other lactic acid producers were reported across disorders, including higher *Enterococcus* and *Streptococcus* in MDD and BD, higher and *Escherichia/Shigella* in MDD and SZ and higher *Bifidobacterium* in BD. These bacteria are generally considered beneficial to the host and can regulate metabolism, protect from pathogenic invasion, and have immunomodulatory effects [127, 128]. Lactic acid-producing bacteria also provide lactate for bacteria that use this molecule as a substrate to produce metabolites, such as the SCFA butyrate [129], in a process known as ‘cross-feeding’. However, there are some circumstances in which lactate production and utilisation may be detrimental to host health. Accumulation of lactate in the gut is potentially deleterious and associated with acidosis, cardiac arrhythmia and neurotoxicity [129, 130]. Many psychiatric disorders are associated with dysregulated mitochondrial energy generation, indexed by increased lactate and decreased pH (i.e. increased acidity) in the brain [131]. Increased faecal lactate is also associated with GI diseases such as short bowel disease and ulcerative colitis, whereas faecal lactate is seldom detected under normal conditions [129, 130]. Increased lactic acid production is a well described phenomenon in SZ and BD and is linked to mitochondrial dysfunction [132]. Lactate is also able to cross the blood brain barrier [133]; increased levels of lactic acid have been found in the brains of patients with MDD [134], and higher brain lactate levels have been observed in post-mortem brains of people with BD and SZ [131, 135]. We also observed higher abundances of bacterial genera that utilise lactate across studies, including *Megasphaera* in BD and SZ, and *Escherichia/Shigella* and *Veillonella* in SZ and MDD, which may indicate a compensatory mechanism in response to increased lactate production. Thus, we speculate that increased abundances of lactic acid-producing bacteria, such as those observed in this review, may influence mental disorder pathophysiology via lactate accumulation.

However, it should be noted that lactate has alternative metabolic fates, which further highlights the complex nature of the gut microbiome ecosystem and cross-feeding. For example, this systematic review also identified consistently higher abundances of *Veillonella* and *Megasphaera* in mental disorders. Species within these genera metabolise lactate to the SCFAs propionate and acetate while producing hydrogen [136]. Whilst propionate has been hypothesised to have antidepressant effects, excess propionate has been associated with increased depressive-like behaviours in animal studies [137] and elevated levels of propionate have been reported in Alzheimer’s disease [138]. In addition, it has been hypothesised that a by-product of lactate metabolism—hydrogen—may also influence host physiology [130, 139]. Hydrogen cross-feeding can occur with sulphate-reducing bacteria (SRB), methanogenic archaea, and acetogenic bacteria, which respectively produce hydrogen sulphide, methane and acetate [140]. Microorganisms that produce hydrogen sulphide (e.g. *Desulfosporosinus*, *Desulfotomaculum*, *Desulfovibrio*) and methane (e.g. *Methanobrevibacter*) have been reported to be in higher abundance in those with mental disorders [70, 81, 97, 102, 106, 112]. Functional pathways associated with methanogenesis, methane metabolism, and methane oxidation, have also been reported as enriched in mental disorders [66, 71, 95, 106]. Research investigating the influence of SRB and methanogens and their associated metabolites on health are inconsistent; both have been associated with both positive and negative health outcomes, but are hypothesised to be pro-inflammatory [140, 141]. Future studies employing metabolomics, alongside gut microbiome composition and functional analyses, are required to further our understanding of the potential role of the gut microbiome and lactate metabolism pathways in mental disorder pathophysiology.

#### Evidence of reduced bacteria with the potential to produce butyrate

Our trans-diagnostic approach identified lower levels of the butyrate-producing bacteria *Coprococcus* across all three mental disorders. Again, there was very little evidence to suggest this pattern was particularly associated with any specific disorder. Moreover, lower *Faecalibacterium* was a shared feature of MDD and BD, and lower *Roseburia* was a shared feature of BD and SZ; these bacteria are also butyrate producers. These findings are concordant with a Dutch study that identified *Faecalibacterium* and *Coprococcus* as positively correlated with quality-of-life scores in two large independent cohorts [115]. *Coprococcus* was also identified as lower in participants with general practitioner- or self-reported depression, even when controlling for the use of anti-depressants [115], which—like antipsychotics and anticonvulsants—have documented antimicrobial effects [142]. Similarly, a large US study reported positive associations between *Coprococcus* and *Faecalibacterium* and a ‘health-related’ group of host factors [143]. Lower *Roseburia* levels have been observed in epilepsy and post-traumatic stress disorder, however inconsistent findings have been observed for autism spectrum disorder and Parkinson’s disease [144]. Our findings are concordant with those observed across other mental disorders, which commonly report lower levels of faecal butyrate as well as reduced levels of butyrate-producing bacteria [144].

The potential role of butyrate-producing bacteria has been extensively studied [145, 146]. The production of butyrate and other SCFAs by host bacteria is primarily derived from the anaerobic fermentation of dietary fibre in the gut [147]. However, *Roseburia* species can produce butyrate via degradation of the mucin layer of the gut [148]. Butyrate is a SCFA understood to confer health benefits predominantly through influencing the immune system and intestinal homeostasis [149]. Butyrate is the primary source of energy for colon cells and plays an important role in maintaining gut barrier integrity. Butyrate receptors are also highly expressed throughout the body, especially on immune and endocrine cells [148]. Thus, it is possible that reduced butyrate production may contribute to the impaired gut barrier permeability and subsequent bacterial translocation into the systemic circulation, alongside systemic inflammation, that have been implicated [150] in, and observed [151] in mental disorders. Importantly, high fibre dietary interventions that have already demonstrated efficacy in improving outcomes in moderate to severe MDD [32] also increase butyrate-producing bacteria [152].

#### Evidence of enriched bacteria with the potential to influence GABA metabolism

Our review also indicated that there were higher levels of bacteria associated with the metabolism of glutamate and γ-aminobutyric acid (GABA) across all three mental disorders. Again, there was very little evidence to suggest this pattern was particularly associated with any specific disorder, with higher *Lactobacillus* a common feature across all disorders. Higher abundances of *Alistipes* and *Parabacteroides* were a feature of MDD, higher *Bifidobacterium* a feature of BD, higher *Enterococcus* a feature of both MDD and BD and lower *Bacteroides* and *Streptococcus* a feature of SZ; these bacteria are associated with glutamate and GABA metabolism.

The previously mentioned lactic acid-producing bacteria *Lactobacillus*, *Bifidobacterium* and *Enterococcus* contain genes encoding glutamate decarboxylase (GAD) enzymes, which catalyse the reaction of L-glutamate to GABA [153, 154]. *Eggerthella* species are less commonly studied, however may also influence glutamate metabolism via GAD, and higher levels of *Eggerthella* have been associated with changes in glutamate metabolism in children with autism spectrum disorder [155]. In addition, *Bacteroides*, *Escherichia* and *Parabacteroides* have also been associated with GABA production [156]. It is possible that these gut bacteria observed in higher abundances across mental disorders may facilitate greater utilisation of glutamate (i.e. depletion) and increased synthesis of GABA.

The pathophysiological implications of differential abundances of specific bacteria remains to be confirmed. This highlights the need for multi-omics approaches to better understand the dynamic and complex functionality of the human gut microbiota. In addition, whether gut microbiota differences are the cause or consequence of pathophysiology, or are jointly influenced by shared risk factors such as diet, requires further exploration. Future longitudinal cohort studies will afford the documentation of changes in the gut microbiota and their relationship to disease development and may help to determine causality. Finally, intervention studies may help to further elucidate the mechanistic and biochemical implications of specific bacterial taxa on host health and disease.

#### Methodologies across studies are highly heterogenous and lacking reproducibility

This review highlights the significant heterogeneity in the collection and reporting of human microbiota data. As the field is rapidly evolving, consensus on best-practice methodologies is constantly changing or being superseded, making the development or identification of ‘gold-standards’ complex. Budgetary constraints often influence study design, and studies often have relatively small sample sizes. Given the lack of established power calculation protocols for microbiome studies, it is often unclear as to whether they are adequately powered to detect differences. Due to the influence of differing microbiome-related study methodologies on study results [157–159], there is an urgent need for clarity in the reporting of microbiome research, and the consideration of these limitations within individual studies. Factors including, but not limited to, medication use [142] and diet [160, 161] are also strongly associated with changes to the gut microbiome. Therefore, collection of data on these factors and their adequate consideration in analyses and interpretation is imperative. However, as gut microbiota composition is often a secondary study outcome, these factors may not have been included when determining study design and this may explain the lack of collection and consideration of covariates. Using the ‘Strengthening the Organizing and Reporting of Microbiome Studies’ (STORMS) tool [162], a newly developed checklist for the reporting of human microbiome studies, is a necessary step towards enhancing methodological consistency and reproducibility [163].

### Limitations of the current review

Our current review has several methodological characteristics that need to be considered when interpreting these results. Firstly, included studies were comprised of cross-sectional data, which cannot infer causality nor account for temporal variations in the gut microbiome. Secondly, geographical distribution of studies shows a clear overrepresentation of studies conducted in China. Considering different geographic regions are associated with different microbial compositions [164], this imbalance in sampling region may have influenced the results of our synthesis. Thirdly, this systematic review focused only on characterising the bacterial members of the gut microbiome, due to their documented association with host mood and behaviour. As the GI tract harbours a myriad of other microorganisms including archaea, viruses, bacteriophages, and fungi, their potential influence on host mental health, which is newly being investigated [104, 165], should not be overlooked. Fourth, the focus on compositional data within the field of gut microbiome research (primarily using 16S) is a significant limitation due to acknowledged factors such as limited resolution and lower sensitivity [166, 167]. Despite these limitations, these data provide an important foundation for our understanding of the gut microbiome in psychiatry. As the field continues to grow and develop, a greater number of studies using other omics techniques (such as metagenomics, metabolomics, and meta-transcriptomics) will be produced, and thus our ability to explore the gut microbiome beyond its composition will be possible. This may be of particular benefit to the field of psychiatry, which currently lacks biomarkers for diagnosis and prognosis, as well as a clear understanding of disorder aetiology. Fifth, our syntheses may be biased by unmeasured confounding. The collection of covariate data was heterogenous and inconsistent across studies, and very few studies adjusted for potential confounding in their analyses. Finally, this review identified many methodological differences between studies. A comprehensive discussion of these differences is outside the scope of this review, however we have provided a summary of the key differences in the supplementary material.

## Conclusions

To conclude, our systematic review indicated that the mental disorders MDD, BD and SZ were not characterised by differences in the number or distribution (α-diversity) of gut bacteria, however each mental disorder appeared to display overall compositional differences compared to controls (β-diversity). The identification of lower levels of butyrate-producing bacteria, higher levels of lactic acid-producing bacteria, and higher levels of bacteria associated with glutamate and GABA metabolism, was relatively consistent across studies. However, future research employing multi-omics approaches is required to clarify the implications of compositional and taxonomic differences for mental disorder pathophysiology and aetiology. If further research confirms our findings, these bacterial genera may have future diagnostic and prognostic potential. Moreover, these findings may support novel treatment strategies, such as dietary interventions that target the gut microbiome. Finally, there is a clear and urgent need for the harmonisation of reporting and methodologies in the field of human microbiome research. The development of new tools and guidelines holds promise for achieving consistency and reproducibility, and for improving our understanding of the role of the gut microbiota in psychiatry.

## Supplementary information

Supplementary Tables S1-S9

Supplementary Figures S1-S5
