# Supplementary material for: A systematic review of gut microbiota composition in observational studies of major depressive disorder, bipolar disorder and schizophrenia
Source: Mol Psychiatry. 2022 Feb 22;27(4):1920–35. doi: 10.1038/s41380-022-01456-3 (PMC9126816; doi:10.1038/s41380-022-01456-3)
Supplement: Supplementary file 1 — Supplementary Tables S1-S9 [file 41380_2022_1456_MOESM1_ESM.pdf]

Supplementary Tables

Table S1: Characteristics of studies investigating gut microbiota composition in major depressive disorder (MDD)
A summary of individual MDD study participant characteristics including age; sex; BMI; disorder diagnosis and severity; case/control definitions; and inclusion/exclusion criteria.

Table S2: Characteristics of studies investigating gut microbiota composition in bipolar disorders (BD)
A summary of individual BD study participant characteristics including age; sex; BMI; disorder diagnosis and severity; case/control definitions; and inclusion/exclusion criteria.

Table S3: Characteristics of studies investigating gut microbiota composition in schizophrenia (SZ)
A summary of individual SZ study participant characteristics including age; sex; BMI; disorder diagnosis and severity; case/control definitions; and inclusion/exclusion criteria.

Table S4: Associations between gut microbiota composition and psychiatric symptom severity
Overview of associations between gut microbiota composition and psychiatric symptom severity across disorders.

Table S5: Potential gut microbiota covariates collected across studies
A summary of potential covariates commonly collected or excluded across studies, including lifestyle factors, biotics, medication use, GI comorbidities, psychiatric comorbidities, physical comorbidities, and biomarker data.

Table S6: Additional covariate considerations and adjustments
Overview of statistical adjustments across studies

Table S7: Description of gut microbiota collection and processing methods across studies
A summary of the different methodological techniques used across studies including sample collection, transport, storage, preprocessing and sequencing.

Table S8: Brief overview of gut microbiota analyses across studies
A brief overview of commonly used microbiome statistical techniques.

Table S9: Quality assessment of reviewed studies
National Institutes of Health National Heart, Lung, and Blood Institute Study Quality Assessment Tool for Observational Cohort and Cross-sectional Studies

Table S1. Characteristics of studies investigating gut microbiota composition in major depressive disorder (MDD)

Ref	Author (Year)	Country	Diagnostic criterion	Sub-group	Sample Size (N)		Male (%)		Age (mean)		BMI (mean)		Severity index	Description of cases	Description of controls	Exclusion criteria
					MDD	HC	MDD	HC	MDD	HC	MDD	HC				
87	Bai et al. (2021)	China	DSM-IV	N/A	60	60	35%	40%	35.62	35.13	20.9	21.19	HDRS (score >17 inclusion)	Psychiatrist-diagnosed MDD.	HCs from the hospital medical examination centre of the hospital.	**Cases**: HDRS score <17; pre-existing mental disorders or Axis I/II disorders; pregnant or within the first year after delivery; illicit drug use; alcohol abuse; antidepressant treatments 1 month prior to sample collection.<br>**Controls**: Axis I/II disorders; systemic medical illness; illicit drug use; alcohol abuse.
70	Chen et al. (2018)	China	Psychiatrist-diagnosed	Female cohort	24	24	0%	0%	41.5	43.95	22.01	22.6	HDRS-17	First-episode drug-naïve MDD patients recruited from a psychiatric centre.	HCs from a medical examination centre.	**Cases**: obesity; diabetes; substance abuse; pre-existing physical diseases; other mental disorders; receiving nonpharmacologic treatments.<br>**Controls**: previous lifetime history of Axis I/II neurological diseases; systemic medical illness.<br>**All**: using antibiotics or probiotics; pregnant, nursing, or currently menstruating.
				Male cohort	20	20	100%	100%	40.35	42.8	22.22	22.5				
69	Chen et al. (2020)	China	DSM-IV	Young cohort (18-29 years)	25	27	28%	30%	24	24.96	22.13	21.53	HDRS (score =>17 for inclusion)	MDD outpatients from the psychiatric centre of Chongqing Medical University, majority who were first-episode drug-naïve.	HCs from the Medical Examination Centre of Chongqing Medical University.	**Cases:** other mental disorders; illicit drug use; substance abuse; pregnant; menstrual women.<br>**Controls:** mental disorders; illicit drug use; systemic medical illness.
				Middle-aged (30-59 years)	45	44	31%	23%	44.96	47.16	22.64	23.23				
85	Chen et al. (2021)	China	DSM-V	16S rRNA sequencing	62	46	0%	0%	39.58	36.93	21.99	22.24	HDRS-17 (score =>18 inclusion)<br>HAMA<br>PANSS<br>GAF	MDD patients from the Psychiatric Department at Xijing Hospital, Xi'an, China, with a current depressive episode.	HCs with a HAMD-17 score <8, without physical or psychiatric illness.	**All**: prebiotics, probiotics, or antibiotics in the month before enrolment; IBD; diseases of the digestive system; obesity; hyperglycaemia; diagnosis of/treatment for diabetes; triglycerides ≥ 2.3 mmol/l; hypertension; severely imbalanced diet, such as high-fat diet preferences or long-term vegetarians; pregnancy; lactation; other mental disorders; psychotropic substances (except benzodiazepines) for more than 3 consecutive days in the 2 weeks before study enrolment; smoking; alcohol use.
				Metagenomics	20	21	0%	0%	N/R	N/R	N/R	N/R				
71	Chung et al. (2019)	Taiwan	DSM-V	N/A	36	37	22%	38%	45.83	41.19	22.8	23.95	BDI<br>BAI<br>PSS	MDD patients referred by psychiatrists in several central and regional hospitals in Taipei.	HCs collected from community in the same catchment area as cases.	**Cases**: mental retardation; SZ; schizoaffective disorder; substance induced secondary MDD.<br>**Controls**: past diagnosis of major psychiatric disorders (e.g., anxiety disorder, mood disorder, SZ, mental retardation, and substance-use disorder).<br>**All**: using antibiotics, probiotics, prebiotics or symbiotics; known active bacterial, fungal, or viral infections; GI surgery in the previous 2 months.
88	Dong et al. (2021)	China	DSM-V	N/A	23	10	30%	40%	30.04	30.22	21.87	21.45	HDRS-24	MDD outpatients who received treatment at the West China Hospital from January to June 2019 and were newly diagnosed with depression or had not used psychotropic drugs for at least 6 months.	NC subjects included worker volunteers without current or past major psychiatric disorders.	**Cases**: <18 or >45 years; organic aetiology for psychiatric symptoms, psychotic features, or intellectual disability.<br>**Controls**: current or past major psychiatric disorders.<br>**All**: lifetime history of BD, SZ, schizoaffective, or other psychiatric disorders; hypertension; CVD; diabetes mellitus; obesity; liver cirrhosis; fatty liver disease; IBS; IBD; drug or alcohol abuse in the previous year; use of antibiotics, probiotics, prebiotics, or synbiotics in the 6 months before sample collection; known active bacterial, fungal, or viral infections; and obvious dietary preferences (e.g., vegetarians).
72	Huang et al. (2018)	China	ICD-10	N/A	27	27	26%	26%	48.7	42.3	23.8	23.4	N/R	First-episode MDD patients without systemic antidepressant treatment.	HCs matched for age and sex.	**Cases**: depressive episodes caused by organic and substance abuse; those with atypical characteristics.<br>**Controls**: any other mental illness.<br>**All**: chronic disease such as hypertension, diabetes mellitus, metabolic syndrome, immune deficiency, autoimmune disease, cancer; IBD; diarrhea in the last 3 months; antibiotics, glucocorticoids, cytokines, large doses of probiotics and biological agents; gastroscopy, colonoscopy, or barium meal in the digestive tract in the last 6 months; major GI surgery (cholecystectomy, appendicectomy, intestinal tract resection) in the past 5 years; restricted movement due to a major physical or mental illness; significant dietary changes in the last 6 months; gestating women.
73	Jiang et al. (2015)	China	DSM-IV	Active-MDD (A-MDD)	29	30	62%	50%	25.3	26.8	20.3	19.6	HDRS-24 (HDRS =>20 for inclusion)<br>MADRS	Patients recruited from the Seventh People's Hospital of Hangzhou in Hangzhou, Zhejiang.<br>A-MDD: patients defined as having a HDRS score =>20.<br>R-MDD: patients defined as those with a baseline HDRS score =>20 upon admission to the hospital, but with samples collected at the time their HDRS scores showed a 50% reduction after 4 weeks treatment.	HCs from the same cohort with no psychiatric or physical illness.	**All**: hypertension; CVD; diabetes mellitus; obesity; liver cirrhosis; fatty liver disease; IBS; IBD; drug or alcohol abuse in the last year; use of antibiotics, probiotics, prebiotics, or synbiotics in the month before sample collection; known active bacterial, fungal, or viral infections.
				Remitted-MDD (R-MDD)	17	30	53%	50%	27.1	26.8	21.8	19.6				
74	Kelly et al. (2016)	Ireland	DSM-IV	N/A	34	33	62%	58%	45.8	45.8	26.2	24.58	HDRS (score >17 for inclusion)<br>BDI<br>BAI<br>PSS<br>CTE	Depressed patients recruited from outpatient and inpatient psychiatric clinics by a psychiatrist.	HCs matched for gender, age and ethnicity recruited from advertisements directed at staff at Cork University Hospital and University College Cork.	**All**: use of probiotics, antibiotics use in the previous 4 weeks; active infections; glucocorticoids, NSAIDs; diabetes; IBD, IBS; recent GI surgery; arthritis; pregnancy; active alcohol or substance abuse or dependency; inpatient admission greater than 1 week.

75	Lai et al. (2019)	China	DSM-V	N/A	26	29	31%	23%	43.73	39.41	21.17	21.1	HDRS-17 (score >17 for inclusion)<br>HAMA<br>HCL-32	MDD patients from the inpatient and outpatient units of Shenzhen Kangning Hospital, Guangdong, China.	HCs from the nearby communities free of any psychiatric or physical illnesses.	**All**: psychoactive substance abuse; acute or chronic diseases (including stroke, epilepsy, hypertension, endocrine disease, diabetes mellitus, fatty liver disease, or severe VD); combined neurological or physical illness confirmed by physical examination, especially a neurological examination, routine blood test, and a brain computed tomography scan if necessary; specific dietary habits, such as a weight loss diet or completely vegetable-based; specific treatments within 6 months, such as TMC or ECT; use of antibiotic, probiotic, prebiotic, or synbiotic within the past month; BMI>24; pregnancy or breast feeding.
76	Lin et al. (2017)	China	DSM-IV-TR	N/A	10	10	60%	60%	36.2	38.1	23.8	24.2	HDRS-17 (score =>23 for inclusion)	MDD patients who received a single escitalopram treatment of 10 mg once per day.	HCs in good mental and physical health who are unrelated to an individual with MDD.	**Cases**: antibiotic, antifungal medications or any probiotics and probiotics related drink within the last month; change of dose during the study period.<br>**Controls**: stomach/gut problems such as chronic diarrhea, constipation, gas, heartburn, bloating.
77	Liu et al. (2016)	China	DSM-IV	N/A	15	20	27%	35%	44.8	43.9	22	24.6	N/R	Depressed patients from the outpatient department of the Institute of Mental Health of Peking University, evaluated by both a gastroenterologist and a psychologist.	HCs evaluated by both a gastroenterologist and a psychologist.	**Cases**: other types of psychological disorders.<br>**All**: antibiotics, probiotics/prebiotics, and psychotropic medications during the previous 4 weeks; history of systemic or GI tract diseases, such as diabetes mellitus and IBD; current infectious diseases of the respiratory, digestive, or urinary system; history of abdominal surgery. All subjects also underwent colonoscopy or received a barium enema to rule out organic colonic diseases.
78	Liu et al. (2020)	USA	DSM-V	N/A	43	47	12%	28%	21.9	22.1	N/R	N/R	PROMIS (score >21 for inclusion)<br>C-SSRS<br>SITBI	MDD patients meeting diagnostic criteria for a current major depressive episode.	HCs with a PROMIS score <13.	**Controls**: PROMIS score < 13; no lifetime history of MDD; no lifetime history of suicidal ideation, suicide attempts, or non-suicidal self-injury as assessed by the C-SSRS and SITBI.<br>**All**: smoked cigarettes or cigars in the past 12 months; vegan; GI illness in the past 6 months; diarrhea in the past 2 weeks; anti-diarrhea medication in the past 6 weeks; antibiotics in the past 3 months.
79	Mason et al. (2020)	USA	DSM-IV	N/A	14	10	21%	40%	41.9	33	31	25.6	QIDS-SR (score >9 for inclusion)<br>GAD-7	Patients with a primary diagnosis of non-psychotic MDD.	HCs who are psychologically healthy.	**All**: pregnant or breastfeeding; history of psychotic depressive, schizophrenic, schizoaffective, or other Axis I psychotic disorders; current alcohol or substance abuse, dependence, or use disorder; unstable general medical condition; taking antipsychotic medications.
80	Naseribafrouei et al. (2014)	Norway	ICD-10	N/A	37	18	46%	39%	49.2	46.1	25.9	24.7	MADRS	Depressed patients from an inward and outpatient mental health clinic of the Innlandet Hospital in Norway, with mild to severe depression.	HCs with the same age and gender distribution, recruited from an outpatient neurological unit at the same hospital, with diffuse symptoms that could possibly be related to cerebral disorders, but no disorders could be found. They all had a careful neurological examination and CT/ MRI scans.	N/R
81	Rong et al. (2019)	China	DSM-IV	N/A	31	30	71%	47%	41.58	39.47	21.46	21.97	HDRS-17 (score >17 for inclusion)<br>HAMA<br>HCL-32<br>MDQ	MDD patients recruited from the inpatient and out- patient units of Shenzhen Kangning Hospital, Guangdong, China, with a current major depressive episode (HDRS >17).	Healthy controls from the nearby districts without psychiatric or physical illnesses.	**All**: other comorbid mental disorders; history of psychoactive substance abuse; history of stroke, epilepsy, hypertension, endocrine disease, diabetes mellitus, fatty liver disease or severe cardiovascular disease; extreme diet, such as a weight loss diet or vegetarianism; history of transcranial magnetic stimulation (TMS) or electroconvulsive therapy (ECT) treatments within the previous 6 months; pregnancy; history of antibiotic, probiotic, prebiotic or synbiotic use within the previous one month; body mass index (BMI) > 24; unwillingness to provide detailed address or refusal to participate in follow-up.
89	Shen et al. (2021)	China	N/R (MINI)	Baseline - before treatment	30	30	43%	50%	44.83	43.97	23.99	23.83	HDRS (score =>24 for inclusion)	First episode (acute) MDD patients with HDRS => 24 and duration of symptoms between 1 and 24 months	NCs without a diagnosis of a mental disorder and with a HDRS score <7.	**All**: BMI <18 or >28 kg/m2; history of antipsychotic medication; somatic diseases such as IBD, immune system diseases, diabetes; antibiotics, probiotics or microbiological products used in recent 3 months; history of medical examination or surgery through the GI tract in recent 6 months; obvious changes in dietary habits; presence of obvious diarrhea, constipation and other symptoms in recent 1 month.<br>**Cases**: history of treatment with antidepressant medication; presence of other mental disorders such as Axis I, personality disorder and mental retardation; psychotropic drug use.<br>**Controls:** diagnosis of mental disorder (excluded by two psychiatrists according to the MINI); HDRS-17 score was > 7.
				Follow-up - after treatment	/	/	/	/	/	/	/	/	/			
82	Stevens et al. (2020)	USA	DSM-IV	N/A	20	20	50%	30%	34*	34*	N/R	N/R	N/R	Volunteer subjects meeting criteria for depression as outpatients at the University of Florida Department of Psychiatry.	HCs without a diagnosed mental illness.	N/R
86	Yang et al. (2020)	China	DSM-IV	Discovery set	118	118	43%	43%	27.19	26.86	22.5	22.09	HDRS-17<br>QIDS	Patients meeting the DSM-IV criteria for MDD.	HCs recruited through advertisements who did not meet the criteria for any DSM-IV Axis I psychiatric disorder.	**Controls**: any DSM-IV Axis I psychiatric disorder.<br>**All**: a lifetime history of BD, SZ, schizoaffective, or other Axis I psychiatric disorders; diagnostic diseases (e.g., chronic inflammatory disorders, diabetes, CVD, thyroid disease, or cancer); alcohol abuse, drugs abuse, or acute poisoning; current pregnancy or breastfeeding; changes of diet habit or history of antibiotic use within 1 month before sampling.

90	Ye et al. (2021)	China	DSM-IV	Baseline	26	28	19%	25%	26.04	26.04	19.78	21.59	HDRS-17 (score =>24 for inclusion)	MDD patients from the First Affiliated Hospital of China Medical University in Shenyang, Liaoning.	Patients from the First Affiliated Hospital of China Medical University in Shenyang, Liaoning.	**All**: major physical disease history, such as hypertension, diabetes or metastatic tumours, liver cirrhosis, fatty liver, IBS, and IBD; unstable physical illness, severe asthma, abnormal nervous system history, including major head trauma (continuous loss of consciousness lasting longer than 5min), epilepsy, cerebrovascular disease, brain tumours and neurodegenerative diseases; physical diseases that may cause mood disorders, such as multiple sclerosis and thyroid diseases, and autism or extensive developmental disorders; use of antidepressant or antipsychotic drugs during the previous 2 weeks; long-acting antipsychotic drugs or ECT within the previous month; pregnant and lactating women; stress events in the previous week; drug abuse or alcohol abuse in the previous year; use of antibiotics, probiotics, or synbiotics in the previous month; and known active bacterial, fungal or viral infections<br>**Cases**: HDRS-17 score did not decrease by 50% after 4 weeks of vortioxetine hydrobromide treatment in MDD patients.
91	Zhang et al. (2021)	China	ICD-10	N/A	36	45	58%	42%	36.81	39.29	24.47	23.94	HDRS-17 (score >17 for inclusion)	MDD patients with current depressive episode from inpatients at Peking University Huilongguan Clinical Medical School, Beijing Huilongguan Hospital.	HCs from nearby communities without any history of psychiatric disorders or psychosis among their first- degree relatives. Candidates were hospital staff, care workers, and patients' accompanying family members and friends with no consanguinity.	**Cases**: does not meet depressive episode of at least moderate severity by two trained psychiatrists; HAM-D <17; not drug naive or without treatment for ≥1 week and without long-acting antipsychotics >6 months before the study.<br>**Controls**: history of psychiatric disorders or psychosis among their first-degree relatives.<br>**All**: history of persistent infection, allergy, or inflammatory diseases whether systematic or local inflammation; a prior medical history of central nervous system disease, severe head injury, substance abuse or dependence, intellectual disability, and other severe medical records; use of antibiotics or probiotic synbiotics within 30 days of study participation; history of GI surgery or severe congenital abnormalities; night shift or rotating schedule within the past 3 months; history of ECT within the previous 6 months; pregnancy; and comorbidities associated with other sleep disorders (e.g., sleep apnoea).
84	Zheng et al. (2016)	China	DSM-IV-TR	N/A	58	63	79%	37%	40.6	41.8	22	22.6	HDRS-17	MDD patients from the psychiatric centre of the First Affiliated Hospital at Chongqing Medical University.	Demographically matched HCs from the medical examination centre of the First Affiliated Hospital at Chongqing Medical University.	**Cases**: substance abuse; pregnancy, nursing, or current menstruation.<br>**Controls**: history of systemic medical illness or mental disorders or family history of any psychiatric disorder.<br>**All**: using antibiotics or prebiotics.
83	Zheng et al. (2020)	China	DSM-IV	Discovery set	122	171	37%	43%	26.54	26.85	22.41	22.07	HDRS	Unmedicated MDD participants experiencing a depressive episode.	HCs matched for key demographic variables including age, gender, and BMI, recruited from advertising.	**All**: physical or other mental disorders or illicit drug use; antibiotics, probiotics, or prebiotics within 1 month prior to sampling.
92	Zheng et al. (2021)	China	ICD-10	N/A	30	30	40%	43%	30.8	33.37	N/R	N/R	HDRS-24	Outpatients and inpatients with depression from Beijing Hui-Long-Guan Hospital treated for the first time.	HCs without clinically diagnosed GI disease.	**All**: Blood routine, blood biochemistry, and routine examination with abnormalities; antibiotics 4 weeks prior to study; contraceptives, NSAIDs, laxatives/antidiarrhea drugs within the first 2 weeks of enrolment; changed eating habits within the first 4 weeks of enrolment; antidepressants and other antipsychotic drugs in the past month; history of abdominal surgery other than appendicitis; serious physical illness; pregnancy or lactation in women.<br>**Controls**: clinically diagnosed GI disease.<br>**Cases**: Mental illness other than depression

N/A = Not applicable; N/R = Not reported.
A-MDD = Active major depressive disorder; BAI = Beck Anxiety Inventory; BD = Bipolar disorder; BDI = Beck Depression Inventory; BMI = Body mass index; C-SSRS = Columbia-Suicide Severity Rating Scale; CTE = Childhood Traumatic Events; CVD = Cardiovascular disease; DSM = Diagnostic and Statistical Manual of Mental Disorders; ECT = Electroconvulsive therapy; GAD = General Anxiety Disorder questionnaire; GAF = Global Assessment of Functioning; GI = Gastrointestinal; HAMA = Hamilton Anxiety Rating Scale; HC = Healthy controls; HCL = Hypomanic Check List; HDRS = Hamilton Depression Rating Scale; IBD = Inflammatory bowel disease; IBS = Irritable bowel syndrome; ICD = International Classification of Diseases; MADRS = Montgomery Asberg Depression Rating Scale; MDD = Major Depressive Disorder; MDQ = Mood Disorder Questionnaire; NC = Normal controls; NSAIDs = Non-steroidal anti-inflammatory drugs; PANSS = Positive and Negative Syndrome Scale; PROMIS = Patient-Reported Outcomes Measurement Information System; PSS = Perceived Stress Scale; QIDS-SR = Quick Inventory of Depressive Symptomatology-Self Report; R-MDD = Remitted major depressive disorder; SITBI = Self-Injurious Thoughts and Behaviour Interview; SZ = Schizophrenia; TMS = Transcranial magnetic stimulation.
*Median values of both case and control groups combined.

Table S2. Characteristics of studies investigating gut microbiota composition in bipolar disorder (BD)

Ref	Author (Year)	Country	Diagnostic criterion	Sub-group	Sample Size (N)		Male (%)		Age (mean)		BMI (mean)		Severity index	BD Type	BD State	Description of cases	Description of controls	Exclusion criteria
					MDD	HC	MDD	HC	MDD	HC	MDD	HC						
93	Coello et al. (2019)	Denmark	ICD-10<br>DSM-V	BD vs HCs	113	77	38%	29%	31	29	24.8	24.2	HDRS-17<br>YMRS	BD I (38.9%)<br>BD II (57.5%)<br>Single manic episode (2.7%)	Remission (60.7%)<br>Affective episode (38.9%)<br>Depressive episode (23.0%)<br>Mixed episode (5.3%)<br>Hypomanic episode (7.1%<br>Manic episode (2.5%)<br>N/A (0.9%)	Patients with newly diagnosed or first-episode BD recruited in the Copenhagen Affective Disorder Clinic.	HCs: age- and sex-matched HCs recruited among blood donors at the Blood Bank at Rigshospitalet, Copenhagen, which covers the same catchment area as the Copenhagen Affective Disorder Clinic.<br>Non-BD relatives: siblings and offspring of the included patients with BD.	**Healthy controls**: Personal or first-degree family history of psychiatric disorders that had required treatment.<br>**Non-BD relatives**: diagnosis lower than F34 including substance abuse, psychotic illnesses, and mood disorders.
				BD vs non-BD relative	113	39	38%	46%	31	28	24.8	24.4						
94	Evans et al. (2017)	USA	DSM-IV	N/A	115	64	28%	38%	50.2	48.6	29.3	26	PHQ-9<br>ASRM<br>SF-12<br>GAD-7	BD I (66.1%)<br>BD II (25.2%)<br>BD NOS (8.7%)	N/R	BD individuals recruited from the Heinz C. Prechter Longitudinal Study of Bipolar Disorder at the University of Michigan Depression Centre.	Unaffected HCs who were willing to return stool samples.	N/R
95	Hu et al. (2019)	China	DSM-IV-TR	N/A	52	45	52%	52%	24.15	36.29	21.58	22.37	HDRS-17 (score =>14 for inclusion)<br>MADRS<br>YMRS	BD I (23.08%)<br>BD II (73.08%)<br>BD NOS (3.85%)	N/R	BD patients with a current depressive episode, recruited from the Psychiatric Department of the First Affiliated Hospital, School of Medicine, Zhejiang University, who were either first-episode or psychotropic drug free for at least 3 months.	HCs with no psychiatric disorder or a family history of psychiatric disorder.	**Cases**: any other psychiatric comorbidities.<br>**All**: severe physical diseases involving the heart, lung, liver, and gut; acute and chronic infections; substance abuse disorders; pregnant or breast-feeding; use of antibiotics, probiotics, or prebiotics for less than 4 weeks before sample collection.
97	Lai et al. (2021)	China	DSM-V	N/A	25	28	56%	46%	36.92	39.21	22.11	21.14	HDRS-17 (score >17 for inclusion)<br>HAMA<br>HCL-32<br>MDQ	BD I (72%)<br>BD II (25%)	Current depressive episode (HDRS >17)	BD patients recruited from the inpatient and outpatient units of Shenzhen Kangning Hospital, China.	Age- and sex-matched HCs from nearby communities without a personal or first-degree family history of psychiatric disorders.	**All**: a diagnosis of severe CVD, diabetes mellitus, obesity, and liver cirrhosis; psychoactive substance abuse in the last year; specific treatments (i.e., TMS or ECT within the prior 6 months); use of antibiotics, probiotics, prebiotics or synbiotics in the month before sample collection; pregnant or breast feeding; specific dietary habits (e.g., weight loss diet or completely vegetable-based).
96	McIntyre et al. (2019)	Canada	DSM-V	N/A	23	23	BD1:40%<br>BD2:12.5%	30%	BD1: 43.5<br>BD2: 49	43.75*	BD1: 31<br>BD2: 28*	26*	HDRS-17<br>YMRS<br>CTQ	BD I (65.2%)<br>BD II (34.8%)	Most exhibiting clinically significant depressive symptoms, and none exhibiting manic/hypomanic symptoms.	Participants with BD recruited from the St. Joseph's Healthcare system in Hamilton, Ontario, and the Mood Disorder Psychopharmacology Unit (MDPU) at the University Health Network (UHN) in Toronto, Ontario.	Age- (+/− 5 years) and sex-matched HCs from the Hamilton community, with no current or past history of mental or major medical disorders.	**All**: diagnosis of a major organic GI, autoimmune, rheumatological or immunological disorder; a primary diagnosis other than BD; currently meeting criteria for an eating disorder, substance use disorder, major neurocognitive disorder, and/or delirium; currently pregnant and/or breastfeeding; consumption of antibiotics and/or probiotics within 4 weeks preceding study enrolment; including regular consumption (>3 days/week) of yogurt or milk products fortified with probiotics.
81	Rong et al. (2019)	China	DSM-V	N/A	30	30	50%	47%	38.4	39.47	21.92	21.97	HDRS-17 (score >17 for inclusion)<br>HAMA<br>MDQ<br>HCL-32	N/R	Current depressive episode (HDRS >17)	Depressed BD patients recruited from the inpatient and out-patient units of Shenzhen Kangning Hospital, Guangdong, China, with a current major depressive episode (HDRS >17).	HCs from the nearby districts were without psychiatric or physical illnesses.	**All**: other comorbid mental disorders; history of psychoactive substance abuse; history of stroke, epilepsy, hypertension, endocrine disease, diabetes mellitus, fatty liver disease or severe CVD; extreme diet, such as a weight loss diet or vegetarianism; history of TMS or ECT treatments within the previous 6 months; pregnancy; antibiotic, probiotic, prebiotic or synbiotic use within the previous 1 month; BMI > 24; unwillingness to provide detailed address or refusal to participate in follow-up.
83	Zheng et al. (2020)	China	DSM-IV	Discovery set	169	171	50%	43%	25.59	26.85	21.77	22.07	YMRS	N/R	N/R	Unmedicated BD patients with a current depressive, but not manic, episode.	HCs matched for age, gender, and BMI, recruited from advertising in two centres.	**All**: physical or other mental disorders; illicit drug use; antibiotics, probiotics, or prebiotics within 1 month prior to sampling.

N/A = Not applicable; N/R = Not reported.
ASRM = Altman Self-Rating Mania scale; BD = Bipolar disorder; BD1 = Bipolar disorder type 1; BD2 = Bipolar disorder type 2; BMI = Body mass index; CTQ = Childhood Trauma Questionnaire; CVD = Cardiovascular disease; DSM = Diagnostic and Statistical Manual of Mental Disorders; ECT = Electroconvulsive therapy; GAD = General Anxiety Disorder questionnaire; GI = Gastrointestinal; HAMA = Hamilton Anxiety Rating Scale; HC = Healthy controls; HCL = Hypomanic Check List; HDRS = Hamilton Depression Rating Scale; ICD = International Classification of Diseases; MADRS = Montgomery Asberg Depression Rating Scale; MDD = Major Depressive Disorder; MDQ = Mood Disorder Questionnaire; NOS = Not otherwise specified; PHQ = Patient Health Questionnaire; SF = Short Form; TMS = Transcranial magnetic stimulation.
*Median values of both case and control groups combined.

Table S3. Characteristics of studies investigating gut microbiota composition in schizophrenia (SZ)

Ref	Author (Year)	Country	Diagnostic criterion	Sub-group	Sample Size (N)		Male (%)		Age (mean)		BMI (mean)		Severity index	SZ State	Description of cases	Description of controls	Exclusion criteria
					MDD	HC	MDD	HC	MDD	HC	MDD	HC					
98	Li et al. (2020)	China	DSM-IV-TR	N/A	82	80	56%	49%	42.15	41.03	24.48	23.03	PANSS	N/R	SZ patients from Guangzhou Huiai Hospital, with symptoms steady >2 weeks, and the PANSS evaluated rate of change ≤20% in 2 weeks and the total score of PANSS ≥30.	HCs of Han nationality with no specific religious beliefs, from Guangzhou and surrounding areas through multiple methods, including recruitment flyers in the community, internet ads and word-of-mouth, and who were age, sex and nationality matched with cases.	**Cases**: any other psychiatric Axis I disorder including schizoaffective disorder, mental retardation, MDD, BD, delirium, dementia, memory disorder and other cognitive disorders; constipation, diarrhea, diabetes, hypertension, heart disease, thyroid diseases or any somatic diseases; a history of epilepsy, except for febrile convulsions; ECT in the past 6 months; lactating, pregnant, or planning to become pregnant; alcohol dependence or noncompliant drug administration; lack of legal guardians.<br>**Controls**: antibiotic intake in the last 3 months; diarrhoea; chronic disease; BMI <18 or >30; any major GI tract surgery within 5 years; any head surgery and/or mental disorders.
108	Li et al. (2021)	China	DSM-IV-TR	N/R	38	38	53%	58%	35.26	35.47	23.7	22.63	PANSS	Stable	SZ patients from the Affiliated Brain Hospital of Guangzhou Medical University with stable symptoms for>2 weeks and a total PANSS score of≥30 with a rate of change of ≤ 20% at 2 weeks.	Age, sex, and BMI matched HCs.	**All**: any other current major Axis I diagnoses; any somatic diseases; a history of epilepsy, except for febrile convulsions; a history of having received ECT in the past 6 months; lactating, pregnant, or planning to become pregnant; alcohol dependence or noncompliance with drug administration or a lack of legal guardians.
99	Ma et al. (2020)	China	DSM-IV	First-episode SZ	40	69	54%	54%	24.19	23.14	N/R	N/R	PANSS	FSCZ (32%) TSCZ (68%)	FSCZ patients: illness duration ≤12 months and no treatment with antipsychotic medications or other psychotropics previously.<br>TSCZ patients: illness duration > 12 months and received antipsychotic treatment for at least the past 3 months.	HCs without any physical or other mental disorders during the lifetime.	**Cases**: Patients with any other psychiatric diagnoses or any physical disorders.<br>**All**: antibiotics, probiotics, or prebiotics within 1 month before sampling.
				Treated SZ	85	69	N/R	/	N/R	/	N/R	/					
109	Manchia et al. (2021)	Italy	DSM-IV-TR	Treatment resistant	18	20	89%	65%	44	37.7	27.3	22.63	N/R	N/R	SZ patients from community mental health centre of the Section of Psychiatry of the Department of Medical Science and Public Health, University of Cagliari, and University Hospital Agency of Cagliari.	HCs from community mental health centre of the Section of Psychiatry of the Department of Medical Science and Public Health, University of Cagliari and University Hospital Agency of Cagliari, without a personal history of mental disorders.	**All**: presence of acute infections; presence of chronic autoimmune inflammatory conditions (e.g., rheumatoid arthritis, thyroiditis); presence of eating disorders; presence of PTSD; presence of current substance use disorders; presence of neurological disorders; past traumatic brain injury; presence of severe co-morbidities that may influence molecular testing (such as cancer, HIV infection); antibiotics in the 3 months preceding the sampling procedure or chronic use of probiotics.<br>**Controls**: personal history of mental disorders
				Responders	20	/	90%	/	50	/	26.9	/					
110	Miao et al. (2021)	China	DSM-IV	N/A	100	90	41%	36%	22.6	23	21	21.2	PANSS (score =>60 for inclusion)	N/A	SZ patients treated in the First Affiliated Hospital of Zhengzhou University.	HCs treated in the First Affiliated Hospital of Zhengzhou University.	**All**: suffer from chronic diseases e.g., hypertension, heart disease, diabetes, or other neuropsychiatric disease; take folic acid or vitamin B12 related drugs within 1 month; take immunosuppression within 3 months; diarrhoea or abnormal stool routine examination; pregnant or lactating women.
100	Nguyen et al. (2019)	USA	DSM-IV-TR	N/A	25	25	56%	60%	52.9	54.7	31.8	28.9	SAPS SANS PHQ-9 SF-36 CIRS	N/R	Outpatients with SZ or schizoaffective disorder, referred to collectively as SZ.	Demographically matched NCs recruited by an ongoing survey study of successful aging in healthy adults.	**Controls**: past or present diagnosis of a major neuropsychiatric illnesses.<br>**All**: other current major Axis I diagnoses; alcohol or other substance (other than tobacco) abuse or dependence within 3 months prior to enrolment; diagnosis of dementia, intellectual disability disorder, or a major neurological disorder; any medical disability that interfered with a subject's ability to complete study procedures.
107	Nguyen et al. (2019)	USA	DSM-IV-TR	N/A	48	48	60%	60%	53.2	54.1	31.8	28.5	SAPS SANS PHQ-9 SF-36 CIRS	N/R	People diagnosed with SZ based on the DSM-IV-TR.	NCs screened for major neuropsychiatric disorders and excluded if they had a past or present diagnosis of a major neuropsychiatric illness.	**All**: other current major Axis I diagnoses; alcohol or other substance (other than tobacco) abuse or dependence within 3 months prior to enrolment; diagnosis of dementia, intellectual disability disorder, or a major neurological disorder; any medical disability that interfered with a subject's ability to complete study procedures.
101	Pan et al. (2020)	China	DSM-IV	N/A	29	29	34%	34%	34.9	34.8	23.7	23.5	PANSS	N/R	SZ patients of Han nationality and local residents of Anhui, China, with PANSS <60 and no change in antipsychotic regimen in the past 2 weeks.	HCs from the communities where the hospital was located, matched by age (±3), sex and BMI.	**Cases**: combined with other mental illnesses; history of smoking or drinking; significant changes in eating habits in the past 2 weeks; GI surgery, gastroscopy, colonoscopy, or GI barium in the past 5 years.<br>**All**: used antibiotics, corticosteroids, cytokines, probiotics, or prebiotics in the past 2 weeks; no chronic physical diseases such as cancer, high blood pressure, diabetes, autoimmune diseases, IBD, and other diseases; no diarrhea, fever in the past 2 weeks.

102	Shen et al. (2018)	China	ICD-10	N/A	64	53	56%	66%	42	39	23.49	23.14	PANSS (score <=60 for inclusion)	N/R	Patients with SZ with an illness duration ≤10 years and received antipsychotic drugs treatment >6 months in hospital or outpatient clinic, with psychiatric symptoms steady for 3 months, and the PANSS evaluated the rate of change ≤20% and the total score of PANSS ≤60.	HCs of Han nationality who were local residents of Huludao area in China with no special religious beliefs.	**Cases**: diagnosis of schizoaffective disorder and other SZ spectrum disorders<br>**All**: chronic disease e.g., hypertension, diabetes, immunodeficiency, autoimmune diseases, cancer, IBD, diarrhea in the last 3 months; absence of any specific drug use for the latest 6 months, including antibiotics, glucocorticoids, cytokines, large doses of probiotics and biological agents; absence of gastroscopy, colonoscopy or GI barium meal; absence of major surgery with GI tract within 5 years; absence of activity limitation caused by major physical disease or psychiatric symptoms; absence of significant changing in dietary habits and middle or high doses of alcohol abuse or dependence.
103	Xu et al. (2020)	China	DSM-V	Validation set	44	44	64%	57%	35	35	22	23.09	N/R	N/R	Patients diagnosed with SZ recruited from Longgang Central Hospital of Shenzhen and Shenzhen Kangning Hospital in Shenzhen, China.	HCs who were age- and sex-matched and unrelated to the patients with SZ.	**All**: genetic metabolic diseases; brain injury; acute physical illness; antibiotic use and/or drug use for GI diseases in the 2 weeks prior to starting the study.
111	Yuan et al. (2021)	China	DSM-IV	Baseline	107	107	48%	35%	19	23	20.71	21.17	PANSS (score >60 for inclusion)	First-episode drug-naïve	First-episode, drug-naïve SZ patients.	HCs recruited from local communities through online advertisement; matched to SZ patients by age, gender, education, smoking habits, and BMI.	**All**: diagnosis of autoimmune diseases, heart diseases, hepato-biliary and GI diseases, blood diseases, diabetes (type I and type II), neurological diseases, mental retardation, or other psychiatric diseases; pregnant or lactating; treated with any antibiotics or anti-inflammatory agents in the previous month; obese (body mass index, BMI > 28 kg/m2).
				After treatment	60	/	/	/	/	/	/	/					
104	Zhang et al. (2019)	China	DSM-IV	N/A	10	16	60%	56%	37.6	35.8	23.3	22.3	PANSS (score >60 for inclusion)	First-episode drug-naïve	First-episode SZ patients enrolled from the Seventh People's Hospital of Hangzhou in Hangzhou, Zhejiang, without antipsychotic treatment, and having never been on any other psychotropic treatment (antidepressant, mood stabilizer, or benzodiazepine).	HCs were recruited from the local community at the same time through advertising.	**All**: BMI > 24 kg/m2; history of systemic diseases or co-morbidities; alcohol or drug abuse during the past year; use of probiotics, prebiotics, synbiotics, or antibiotics in the previous 2 months before collection of the faecal sample; known active bacterial, fungal, or viral infections.
105	Zheng et al. (2019)	China	DSM-IV	N/A	63	69	67%	52%	43.49	39.99	22.9	23.16	PANSS	N/R	SZ patients recruited from the First Affiliated Hospital of Chongqing Medical University.	HCs recruited from the First Affiliated Hospital of Chongqing Medical University.	**All**: any physical or other mental disorders or illicit drug use; antibiotics, probiotics, or prebiotics within 1 month before sampling.
106	Zhu et al. (2020)	China	DSM-IV	N/A	90	81	51%	51%	28.6	33	20.64	21.69	PANSS	Acutely relapsed or first-episode	Acutely relapsed SZ within the last three months (ARSCZ) and first-episode SZ (FESCZ) patients with an episode in the past year, recruited from inpatients at five clinical centres in the Shaanxi Province, China.	HCs recruited from native residents of communities and villages in same the city where the clinical centre was located, not diagnosed with any mental disorders and selected to be well-matched to the patients in relation to demographic features, socio-economic levels, alcohol and tobacco use, and diet habit.	**All**: current physical illness (such as diabetes, heart disease, thyroid disease, autoimmune disease, or any recent infections) or axis I or axis II disorders (except SZ in patients); prescribed medications that could affect the central nervous, endocrine, or immune system; pregnant; acute digestive tract disorder during the last 30 days; antibiotics during the last 30 days.
112	Zhu et al. (2021)	China	DSM-V	Acute	42	44	40%	36%	39.8	42.1	22.3	24.3	PANSS (score =>60 for inclusion)	First-episode drug-naïve	Patients who had been hospitalised; the first episode of SZ without anti-psychotic treatment	HCs recruited by the hospital physical examination centre from the local community at the same time as other participants through advertising.	**All**: a history of craniocerebral trauma, organic cerebral diseases, physical diseases, or other mental disorders; a history of alcohol or other substance use; diabetes, hypertension, dyslipidaemia, endocrine disease, or known medical conditions that might affect metabolism; pregnant or lactating women; a history of digestive tract diseases, abdominal surgery, or intestinal infection in the past 3 months; administration of ECT without convulsions before enrolment; diet change significantly in the past 6 months; treatment with antibiotics or corticosteroids, probiotic preparations, or other immune preparations in the past 3 months.
				Remission	40	/	40%	/	41.1	/	24.3	/	PANSS (score <60 for inclusion)	Remitted	SZ patients who had been hospitalised with a total course of disease <10 years, received only second-generation antipsychotic, such as risperidone, quetiapine, and aripiprazole, and whose clinical symptoms disappeared after treatment and self-consciousness and social function had recovered for at least 3 months.		

N/A = Not applicable; N/R = Not reported

BD = Bipolar disorder; BMI = Body mass index; CIRS = Cumulative Illness Rating Scale; ECT = Electroconvulsive therapy; FSCZ = First episode schizophrenia; GI = Gastrointestinal; HIV = Human immunodeficiency virus; IBD = Inflammatory bowel disease; MDD = Major depressive disorder; PANSS = Positive and Negative Syndrome Scale; PHQ = Patient Health Questionnaire; PTSD = Post-traumatic stress disorder; SAPS = Scale for the Assessment of Positive Symptoms; SANS = Scale for the Assessment of Negative Symptoms; SF = Short Form; SZ = Schizophrenia; TSCZ = Treated schizophrenia;

Table S4. Associations between gut microbiota composition and psychiatric symptom severity

Ref	Author (Year)	Correlations with diversity	Taxa positively correlated with symptom severity	Taxa negatively correlated with symptom severity
**Major depressive disorder (MDD)**				
87	Bai et al. (2021)	N/R	N/R	N/R
70	Chen et al. (2018)	N/R	Collinsella (HDRS, Males)	Clostridium XIVa (HDRS, Females)<br>Erysipelotrichaceae incertae sedis (HDRS, Females)<br>Streptococcus (HDRS, Females)<br>Veillonella (HDRS, Males)<br>Veillonella (HDRS, all MDD combined)
69	Chen et al. (2020)	N/R	N/R	N/R
85	Chen et al. (2021)	N/R	Anaeroglobus (PANSS, HDRS)<br>Anaerotruncus (HDRS)<br>Lachnospiraceae_UCG-006 (PANSS)<br>Parabacteroides (HDRS)	Agathobacter (PANSS)<br>Butyricicoccus (PANSS)<br>Fusicatenibacter (HDRS)<br>Lachnospiraceae_ND3007_group (PANSS)<br>Roseburia (PANSS)<br>Ruminococcaceae_UCG-013 (PANSS)<br>Ruminococcus_1(PANSS)
71	Chung et al. (2019)	N/R	Actinobacteria (PSS)<br>Blautia (BDI, PSS)<br>Clostridium XI (BDI)<br>Eggerthella (BDI, BAI, PSS)<br>Holdemania (PSS)<br>Parabacteroides (BDI, BAI)<br>Peptostreptococcaceae (BDI)<br>Porphyromonadaceae (BDI, BAI)<br>Ruminococcus (BDI, PSS)	Proteobacteria (PSS)<br>Prevotellaceae (BDI, PSS)<br>Alcaligenaceae (BDI, PSS)<br>Prevotella (BDI, PSS)<br>Sutterella (BDI, PSS)
88	Dong et al. (2021)	N/R	/	Christensenellaceae_R7_group (HDRS)
72	Huang et al. (2018)	N/R	N/R	N/R
73	Jiang et al. (2015)	N/R	/	Faecalibacterium (HDRS, all)<br>Faecalibacterium (MADRS, all)
74	Kelly et al. (2016)	N/R	N/R	N/R
75	Lai et al. (2019)	N/R	N/R	N/R
76	Lin et al. (2017)	N/R	/	Klebsiella (HDRS)
77	Liu et al. (2020)	N/R	Bacteroidales (PROMIS)<br>Bacteroidetes (PROMIS)<br>Bacteroidia (PROMIS)<br>Flavonifractor (PROMIS)<br>Gammaproteobacteria (PROMIS)	Firmicutes (PROMIS)<br>Clostridia (PROMIS)<br>Clostridiales (PROMIS)<br>[Eubacterium] coprostanoligenes group (PROMIS)<br>Faecalibacterium (PROMIS)<br>Ruminococcaceae (PROMIS)
78	Liu et al. (2016)	N/R	N/R	N/R
79	Mason et al. (2020)	Non-significant for alpha- and beta-diversity	/	Clostridium leptum group (GAD-7)<br>Eubacteria (GAD-7)<br>Eubacterium rectale/Clostridium cluster XIVa (QIDS-SR)
80	Naseribafrouei et al. (2014)	N/R	N/R	N/R
81	Rong et al. (2019)	Significant for alpha-diversity	N/R	N/R

Ref	Author (Year)	Correlations with diversity	Taxa positively correlated with symptom severity	Taxa negatively correlated with symptom severity
89	Shen et al. (2021)	N/R	N/R	N/R
82	Stevens et al. (2020)	N/R	N/R	N/R
86	Yang et al. (2020)^	N/R	Blautia wexlerae (QIDS)<br>Blautia sp._Marseille-P2398 (QIDS)<br>Ruminococcus sp._5_1_39BFAA (QIDS)<br>Oscillibacter sp. ER4 (HDRS)	/
90	Ye et al. (2021)	N/R	Parasutterella<br>Dialister<br>Bacteroides<br>Prevotella-9<br>Agathobacter	Lachnospira<br>Roseburia<br>Subdoligranulum<br>Faecalibacterium<br>Blautia
91	Zhang et al. (2021)	Yes - HDRS	N/R	N/R
84	Zheng et al. (2016)	N/R	N/R	N/R
83	Zheng et al. (2020)	N/R	/	Peptostreptococcaceae OTU901 (HDRS)
92	Zheng et al. (2021)	N/R	N/R	N/R
**Bipolar disorders (BD)**				
93	Coello et al. (2019)	N/R	N/R	N/R
94	Evans et al. (2017)	N/R	/	Faecalibacterium (PHQ-9)
95	Hu et al. (2019)	N/R	Acetanaerobacterium (MADRS)<br>Anaerotruncus (MADRS)<br>Raoultella (MADRS)<br>Stenotrophomonas (MADRS)	Acinetobacter (MADRS)<br>Cronobacter (MADRS)
97	Lai et al. (2021)	N/R	N/R	N/R
96	McIntyre et al. (2019)	N/R	N/R	N/R
81	Rong et al. (2019)		N/R	N/R
83	Zheng et al. (2020)	N/R	Pseudomonadaceae OTU79 (HDRS)	Lachnospiraceae OTU2572 (HDRS)<br>Lachnospiraceae OTU2016 (HDRS)
**Schizophrenia (SZ)**				
98	Li et al. (2020)^^	N/R	Succinivibrio (PANSS)	Corynebacterium (PANSS)
108	Li et al. (2021)	N/R	N/R	N/R
99	Ma et al. (2020)	N/R	N/R	N/R
109	Manchia et al. (2021)	N/R	N/R	N/R
110	Miao et al. (2021)	No	/	Bifidobacterium
100	Nguyen et al. (2019)#	N/R	Bacteroides (PHQ-9)	Ruminococcaceae (SANS)
107	Nguyen et al. (2021)#	N/R	N/R	N/R
101	Pan et al (2020)	N/R	/	Clostridium sensu stricto 1 (PANSS)
102	Shen et al. (2018)	N/R	N/R	N/R
103	Xu et al. (2020)	N/R	N/R	N/R

Ref	Author (Year)	Correlations with diversity	Taxa positively correlated with symptom severity	Taxa negatively correlated with symptom severity
111	Yuan et al. (2021) - Baseline	No	Lachnoclostridium<br>Firmicutes	/
104	Zhang et al. (2020)	N/R	N/R	N/R
105	Zheng et al. (2019)	N/R	Bacteroidaceae OTU172 (PANSS)<br>Lachnospiraceae OTU477 (PANSS)<br>Lachnospiraceae OTU629 (PANSS)<br>Ruminococcaceae OTU181 (PANSS)<br>Streptococcaceae OTU834 (PANSS)	Ruminococcaceae OTU725 (PANSS)<br>Veillonellaceae OTU191 (PANSS)
106	Zhu et al. (2020)	N/R	N/R	N/R
112	Zhu et al. (2021) - Acute	No	Haemophilus	Coprococcus

^ Adjusted for age and BMI
^^ Adjusted for age, sex and years of education
# Healthy control subjects were matched to schizophrenia subjects on sequencing plate after initial recruitment. Authors found the nearest matching neighbours on the sequencing plate based on age, sex, race, BMI category (obese vs. not obese), and history of antibiotic use (in the past year) to control for clinical factors and known major drivers of microbiome changes that could confound the results.

Table S5. Potential gut microbiota covariates collected across studies

Ref	Author (Year)	Lifestyle factors/ behaviours	Use of biotics	Psychiatric medications	Non-psychiatric medications	Psychiatric co-morbidity	Gastrointestinal co-morbidity	Other physical co-morbidities	Biomarkers
**Major depressive disorder (MDD)**									
87	Bai et al. (2021)	N/R	N/R	**Exclusion**: antidepressant use one-month before sampling	N/R	**Exclusion** (including alcohol abuse)	N/R	**Exclusion** (systemic medical illness)	**Metabolomics**
70	Chen et al. (2018)	N/R	**Probiotics**: exclusion at time of recruitment<br>**Antibiotics**: exclusion at time of recruitment	**Drug-naïve**	N/R	**Exclusion***<br>*Female cohort:*<br>**Anxiety disorder** (n=3)<br>*Male cohort:*<br>**Anxiety disorder** (n=2)	N/R	**Exclusion** (obesity; diabetes; existing physical disease)	N/R
69	Chen et al. (2020)	N/R	N/R	**Young cohort** (28%)<br>**middle-aged cohort** (31%)<br>**Drug naïve total** (70%)	N/R	**Exclusion**	N/R	**Exclusion** (in controls only)	N/R
85	Chen et al. (2021)	**Smoking** (exclusion)<br>**Alcohol** (exclusion)<br>**Diet** (exclusion - participants with a severely imbalanced diet, such as high-fat diet preferences or long-term vegetarians)	**Probiotics**: exclusion month before enrolment<br>**Antibiotics**: exclusion month before enrolment<br>**Prebiotics**: exclusion month before enrolment	**Anti-depressants** (42%) however not for more than 3 consecutive days in the 2 weeks before study enrolment, and without antipsychotics	N/R	**Exclusion** (however, reported high anxiety scale scores)	**Exclusion** (IBD; other diseases of the digestive system such as Crohn's disease and gastritis)	**Exclusion** (obesity, hyperglycaemia; diabetes; hypertension)	N/R
71	Chung et al. (2019)	**Smoking**<br>**Diet** (FFQ - compared major food groups and fatty acids between cases and controls)	**Probiotics**: exclusion at time of recruitment<br>**Antibiotics**: exclusion at time of recruitment<br>**Prebiotics**: exclusion at time of recruitment<br>**Synbiotics**: exclusion at time of recruitment	N/R	N/R	N/R	**Exclusion** (GI surgery in past 2 months)	**Exclusion** (known active bacterial, viral, or fungal infection)	N/R
88	Dong et al. (2021)	**Diet** (exclusion obvious dietary preferences e.g., vegetarian)	**Probiotics**: Exclusion 6 months before sampling<br>**Antibiotics**: Exclusion 6 months before sampling<br>**Prebiotics**: : Exclusion 6 months before sampling<br>**Synbiotics**: : Exclusion 6 months before sampling	**Exclusion** (last 6 months)	N/R	**Exclusion** (including drug and alcohol abuse)	**Exclusion** (IBS, IBD)	**Exclusion** (chronic and metabolic diseases e.g., hypertension; cardiovascular disease; diabetes mellitus; obesity; liver cirrhosis; fatty liver disease; irritable bowel syndrome; inflammatory bowel disease; active infections)	**Neuroendocrine hormone analysis**
72	Huang et al. (2018)	**Alcohol**<br>**Smoking**<br>**Diet** (participants with no special eating habits; exclusion - no significant dietary changes in past 6 months - exclusion)	**Probiotics**: Exclusion previous 6 months<br>**Antibiotics**: Exclusion previous 6 months	**Unmedicated** (without systematic anti-depressive treatment)	**Exclusion** (drugs that affect gut microbiota have been used in the last 6 months, including antibiotics, glucocorticoids, cytokines, large doses of probiotics and biological agents etc.)	N/R	**Exclusion** (IBD, diarrhoea in past 3 months; gastroscopy, colonoscopy, or barium meal in the digestive tract in the past 6 months; major GI surgery in past 5 years)	**Exclusion** (chronic disease that may affect the stability of the gut microbiota, such as hypertension, diabetes mellitus, metabolic syndrome, immune deficiency, autoimmune disease, cancer, IBD, diarrhea in the last 3 months)	N/R

Ref	Author (Year)	Lifestyle factors/ behaviours	Use of biotics	Psychiatric medications	Non-psychiatric medications	Psychiatric co-morbidity	Gastrointestinal co-morbidity	Other physical co-morbidities	Biomarkers
73	Jiang et al. (2015)	**Smoking**	**Probiotics**: Exclusion month before faecal sample<br>**Antibiotics**: Exclusion month before faecal sample<br>**Prebiotics**: Exclusion month before faecal sample<br>**Synbiotics**: Exclusion month before faecal sample	*A-MDD:*<br>**Anti-depressants** (72%)<br>**Anti-psychotics** (26%)<br>**Opioids** (89%)<br>*R-MDD:*<br>**Anti-depressants** (100%)<br>**Anti-psychotics** (29%)<br>**Opioids** (59%)	N/R	N/R	**Exclusion** (IBS; IBD)	**Exclusion** (hypertension; cardiovascular disease; diabetes mellitus; obesity; liver cirrhosis; fatty liver disease; drug or alcohol abuse in the last year; known active bacterial, fungal, or viral infections)	**BDNF**<br>**IL-6**<br>**IL-1b**<br>**TNF-a**
74	Kelly et al. (2016)	**Smoking**<br>**Sleep** (PSQI)<br>**Alcohol use**<br>**Physical activity** (IPAQ)<br>**Diet** (FFQ - compared major macro- and micro-nutrient intakes between cases and controls)	**Probiotics**: Exclusion<br>**Antibiotics**: Exclusion	**Antidepressants** (SSRI 95%)<br>**Antipsychotic** (37%)<br>**Opioids** (30%)<br>**Mood stabilizers** (10%)<br>**Z-drugs** (15%)	**Exclusion** (glucocorticoids, NSAIDS)	**Anxiety disorder** (n=4).<br>**Exclusion** (active alcohol or substance abuse or dependency)	**Exclusion** (IBS; IBD; recent gastrointestinal surgery)	**Exclusion** (active infection, diabetes, arthritis)<br>**Dyslipidaemia** (n=7)<br>**Hypertension** (n=3)	**IL-6**<br>**IL-8**<br>**TNF-a**<br>**CRP**<br>**KYN/TRP ratio**<br>**AUCg/cortisol**<br>**LBP**
75	Lai et al. (2019)	**Diet** - exclusion specific dietary habits, such as a weight loss diet or completely vegetable-based	**Probiotics**: Exclusion previous month<br>**Antibiotics**: Exclusion previous month<br>**Prebiotics**: Exclusion previous month<br>**Synbiotics**: Exclusion previous month	**Medication** (81%)<br>**SSRIs** (46%)<br>**SNRIs** (27%)<br>**Other antidepressants** (23%)<br>**Atypical antipsychotics** (0%)	N/R	**Exclusion** (psychoactive substance abuse)	N/R	**Exclusion** (acute or chronic diseases including stroke, epilepsy, hypertension, endocrine disease, diabetes mellitus, fatty liver disease, or severe CVD; combined neurological or physical illness; significant abnormal results of physical examination, neurological examination, or routine blood tests)	N/R
76	Lin et al. (2017)	**Smoking**	**Probiotics**: Exclusion previous month<br>**Probiotic-related drink**: Exclusion previous month<br>**Antibiotics**: Exclusion previous month	**Antidepressants** (100%)	N/R	N/R	**Exclusion** (controls only – no stomach/gut problems such as chronic diarrhea, constipation, gas, heartburn, bloating, etc.)	N/R	N/R
77	Liu et al. (2016)	**Diet** (data collected by a gastroenterologist = compared the main diet constitution categorised as either 'balanced', 'high fat/protein' or 'vegetarian' between cases and controls)	**Probiotics**: Exclusion previous 4 weeks<br>**Antibiotics**: Exclusion previous 4 weeks<br>**Prebiotics**: Exclusion previous 4 weeks	**Exclusion**	N/R	**Exclusion**	**Exclusion** (IBS; IBD; organic colon diseases; history of GI diseases; history of abdominal surgery)	**Exclusion** (systemic illness such as diabetes; current infectious diseases of the respiratory, digestive, or urinary system)	N/R

Ref	Author (Year)	Lifestyle factors/ behaviours	Use of biotics	Psychiatric medications	Non-psychiatric medications	Psychiatric co-morbidity	Gastrointestinal co-morbidity	Other physical co-morbidities	Biomarkers
78	Liu et al. (2020)	**Smoking** (exclusion)<br>**Diet** (vegan exclusion)	**Antibiotics**: Exclusion previous 3 months	**Antidepressants** (72%)<br>**Antipsychotics** (5%)<br>**Opioids** (9%)<br>**Other psychiatric** (16%)	**Exclusion** (anti-diarrhoea medication in past 6 weeks)	N/R	**Exclusion** (gastrointestinal illness in past 6 months; diarrhoea in past 2 weeks)	N/R	**IL-6**<br>**IL-1b**<br>**IL-8**<br>**IL-10**<br>**IL-12p70**<br>**IL-17a**<br>**IL-18**<br>**IL-23**<br>**IL-33**<br>**TNF-a**<br>**IFN-a2**<br>**IFN-y**<br>**MCP-1**
79	Mason et al. (2020)	N/R	N/R	**Antidepressants** (64%)<br>**Anti-anxiety** (36%)<br>**Anti-psychotics** (exclusion)	N/R	**Exclusion**	N/R	**Exclusion** (unstable general medical conditions)	N/R
80	Naseribafrouei et al. (2014)	N/R	N/R	**Yes** (some taking depression medication)	**Yes** (some taking blood pressure medication)	N/R	N/R	N/R	N/R
81	Rong et al. (2019)	**Diet** (exclusion extreme diet, such as a weight loss diet or vegetarianism)	**Probiotics**: Exclusion previous month<br>**Antibiotics**: Exclusion previous month<br>**Prebiotics**: Exclusion previous month<br>**Synbiotics**: Exclusion previous month	**Antidepressants** (SSRIs 41.9%; SNRIs 19.4%; 19.4% other antidepressants)<br>**Atypical antipsychotics** (0%)	N/R	**Exclusion**	N/R	**Exclusion** (history of stroke, epilepsy, hypertension, endocrine disease, diabetes mellitus, fatty liver disease or severe cardiovascular disease)	N/R
89	Shen et al. (2021)	**Smoking**<br>**Alcohol**<br>**Diet** (exclusion obvious changes in dietary habits)	**Probiotics**: Exclusion previous 3 months<br>**Antibiotics**: Exclusion previous 3 months	**Exclusion** (history of antipsychotics or antidepressants)	**Exclusion** (microbiological past 3 months)	**Exclusion** (other mental disorders such as axis I, personality disorder and mental retardation)	**Exclusion** (IBD; no history of medical examination or surgery through the gastrointestinal tract in recent 6 months; presence of obvious diarrhea, constipation, and other symptoms in recent 1 month)	**Exclusion** (somatic diseases known to affect the gut microbiota such as inflammatory bowel disease, immune system diseases, diabetes, etc.)	N/R
82	Stevens et al. (2020)	N/R	**Probiotics**: Exclusion month before faecal sample<br>**Antibiotics**: Exclusion month before faecal sample	**Yes** (80%)	N/R	N/R	**Reported no gastrointestinal disorders**	N/R	N/R
86	Yang et al. (2020)	**Diet** (FFQ; exclusion changes in diet habit 1 month before samples)<br>**Smoking**	**Antibiotics**: Exclusion month prior to sampling	**Unmedicated**	N/R	**Exclusion** (lifetime history of bipolar disorder, schizophrenia, schizoaffective, or other Axis I psychiatric disorders)	N/R	**Exclusion** (having diagnostic diseases (e.g., chronic inflammatory disorders, diabetes, cardiovascular disease, thyroid disease, or cancer)	N/R

Ref	Author (Year)	Lifestyle factors/ behaviours	Use of biotics	Psychiatric medications	Non-psychiatric medications	Psychiatric co-morbidity	Gastrointestinal co-morbidity	Other physical co-morbidities	Biomarkers
90	Ye et al. (2021)	N/R	**Probiotics**: Exclusion previous month<br>**Antibiotics**: Exclusion previous month<br>**Synbiotics**: Exclusion previous month	**Exclusion** (use of antidepressant or antipsychotic drugs during the previous 2 weeks; long-acting antipsychotic drugs or electroshock therapy (MECT) within the previous month)	N/R	**Exclusion** (major head trauma; continuous loss of consciousness lasting longer than 5min, epilepsy, cerebrovascular disease, brain tumours and neurodegenerative diseases, autism or extensive developmental disorders, stress events in the previous week; drug abuse or alcohol abuse in the previous year)	**Exclusion** (IBS; IBD)	**Exclusion** (major physical disease history, especially diseases that may be associated with brain tissue changes, such as hypertension, diabetes or metastatic tumours, liver cirrhosis, fatty liver; unstable physical illness, severe asthma, physical diseases that may cause mood disorders, such as multiple sclerosis and thyroid diseases, and autism or extensive developmental disorders; known active bacterial, fungal, or viral infections)	N/R
91	Zhang et al. (2021)	**Smoking**<br>**Sleep** (PSQI)<br>**Diet** (same hospital diet for duration of study)	**Probiotics**: Exclusion month before participation<br>**Antibiotics**: Exclusion month before participation<br>**Synbiotics**: Exclusion month before participation	**Exclusion**: treatment for ≥1 week or long-acting antipsychotics >6 months before the study; history of electroconvulsive therapy within the previous 6 months	N/R	**Exclusion** - prior medical history of central nervous system disease, severe head injury, substance abuse or dependence, intellectual disability, and other severe medical records	**Exclusion** (history of gastrointestinal surgery)	**Exclusion** (test results showing those with history of persistent infection, allergy, or inflammatory diseases whether systematic or local inflammation; other severe medical records; severe congenital abnormalities; comorbidities associated with other sleep disorders e.g., sleep apnoea)	N/R
84	Zheng et al. (2016)	**Smoking**	**Prebiotics**: Exclusion<br>**Antibiotics**: Exclusion	**Anti-depressants** (32.8%)	N/R	**Exclusion** (substance abuse disorders)	N/R	N/R	N/R
83	Zheng et al. (2020)	N/R	**Probiotics**: Exclusion month before faecal sample<br>**Antibiotics**: Exclusion month before faecal sample<br>**Prebiotics**: Exclusion month before faecal sample	**Unmedicated**	N/R	**Exclusion**	N/R	**Exclusion**	N/R
92	Zheng et al. (2021)	**Diet** (exclusion: changed eating habits within the first four weeks of enrollment)	**Antibiotics**: Exclusion month before participation	**Exclusion** (antidepressants and other antipsychotic drugs in the past month)	**Exclusion** (contraceptives, nonsteroidal anti-inflammatory drugs, and laxatives/antidiarrhea drugs within the first two weeks of enrolment)	**Exclusion** (mental illness other than depression)	**Exclusion** (gastrointestinal disease clinically diagnosed (in control group); history of abdominal surgery other than appendicitis (all))	**Exclusion** (blood routine, blood biochemistry, and routine examination abnormalities; Combined with serious physical illness)	N/R
**Bipolar disorder (BD)**									
93	Coello et al. (2019)	**Smoking**<br>**Physical activity** (IPAQ)	N/R	**Antidepressants** (26.5%)<br>**Antipsychotics** (38.0%)<br>**Lithium treatment** (38.9%)<br>**Antiepileptic** (51.3%)<br>**Unmedicated** (12.4%)	N/R	N/R	N/R	N/R	**Serum CRP**
94	Evans et al. (2017)	**Sleep** (PSQI)	N/R	**Yes** (most taking more than one psychiatric medication)	N/R	N/R	N/R	N/R	N/R
95	Hu et al. (2019)	N/R	**Probiotics**: Exclusion month before faecal sample<br>**Antibiotics**: Exclusion month before faecal sample<br>**Prebiotics**: Exclusion month before faecal sample	**Drug-free** (for 3 months or first-episode)	N/R	**Exclusion**	**Exclude** (severe physical diseases of the gut)	**Exclusion** (severe physical diseases of heart, lung, liver; acute and chronic infections)	N/R

Ref	Author (Year)	Lifestyle factors/ behaviours	Use of biotics	Psychiatric medications	Non-psychiatric medications	Psychiatric co-morbidity	Gastrointestinal co-morbidity	Other physical co-morbidities	Biomarkers
97	Lai et al. (2021)	**Diet** (exclusion specific dietary habits e.g., weight loss diet or completely vegetable-based)	**Probiotics**: Exclusion month before sampling<br>**Antibiotics**: Exclusion month before sampling<br>**Prebiotics**: Exclusion month before sampling<br>**Synbiotics**: Exclusion month before sampling	**Medicated** (80%; antipsychotics, anticonvulsants, lithium, antidepressants, within a week)<br>**Drug-free** (20%; for 6-months)	N/R	**Exclusion** (psychoactive substance abuse in the last year)	N/R	**Exclusion** (diagnosis of severe cardiovascular disease, diabetes mellitus, obesity, and liver cirrhosis)	N/R
96	McIntyre et al. (2019)	**Smoking**<br>**Diet** (FFQ)	**Probiotics**: Exclusion within 3 weeks of enrolment<br>**Antibiotics**: Exclusion within 3 weeks of enrolment<br>**Exclusion**: regular consumption (>3 days/week) of yogurt or milk products fortified with probiotics	N/R (authors report medication was allowed but did not report specific medications)	**Yes** (allowed but not reported, except for oral contraception use 14% of cases, 30% controls)	**Exclusion**	**Exclusion** (any organic gastrointestinal disorder;<br>gut symptoms measured using the GSRS over the past week)	**Exclusion** (diagnosis of a major organic medical disorder that may alter the microbiome, specifically any organic GI, autoimmune, rheumatological or immunological disorder)	N/R
81	Rong et al. (2019)	**Diet** (exclusion specific dietary habits e.g., weight loss diet or completely vegetable-based)	**Probiotics**: Exclusion previous month<br>**Antibiotics**: Exclusion previous month<br>**Prebiotics**: Exclusion previous month<br>**Synbiotics**: Exclusion previous month	**Antidepressants** (SSRIs 36.7%; SNRIs 10%;3% other)<br>**Atypical antipsychotics** (23%)	N/R	**Exclusion**	N/R	**Exclusion** (history of stroke, epilepsy, hypertension, endocrine disease, diabetes mellitus, fatty liver disease or severe cardiovascular disease)	N/R
83	Zheng et al. (2020)	N/R	**Probiotics**: Exclusion month before faecal sample<br>**Antibiotics**: Exclusion month before faecal sample<br>**Prebiotics**: Exclusion month before faecal sample	**Unmedicated**	N/R	**Exclusion**	N/R	**Exclusion**	N/R
Schizophrenia (SZ)									
98	Li et al. (2020)	**Smoking**<br>**Alcohol use**	**Antibiotics**: Exclusion previous 3 months (controls only)	**Anti-psychotics** (91.5%)	N/R	**Exclusion**	**Exclusion** (constipation; diarrhoea, major GI surgery in past 5 years (controls only))	**Exclusion** (diabetes, hypertension, heart disease, thyroid diseases, or any somatic diseases; history of epilepsy, except for febrile convulsions; absence of chronic disease that may affect the stability of the gut microbiota (controls only))	**Serum glucose**<br>**Total cholesterol**<br>**Total triglycerides**<br>**Serum HDL**<br>**Serum LDL**
108	Li et al. (2021)	**Smoking**<br>**Alcohol**	N/R	**Anti-psychotics** (92%)	N/R	**Exclusion** (any other current major DSM-IV-TR Axis I diagnoses; history of ECT)	N/R	**Exclusion** (any somatic diseases; a history of epilepsy, except for febrile convulsions)	**HDLC**<br>**LDLC**<br>**Glu**
99	Ma et al. (2020)	N/R	**Probiotics**: Exclusion month before faecal sample<br>**Antibiotics**: Exclusion month before faecal sample<br>**Prebiotics**: Exclusion month before faecal sample	**FSCZ**: Drug-naïve<br>**TSCZ**: antipsychotic treatment for at least past three months	N/R	**Exclusion**	N/R	N/R	N/R

Ref	Author (Year)	Lifestyle factors/ behaviours	Use of biotics	Psychiatric medications	Non-psychiatric medications	Psychiatric co-morbidity	Gastrointestinal co-morbidity	Other physical co-morbidities	Biomarkers
109	Manchia et al. (2021)	**Smoking**<br>**Alcohol**<br>**Diet** (based on Mediterranean diet patterns)<br>**Physical activity**	**Antibiotics**: Exclusion 3 months before sampling<br>**Probiotics**: Exclusion chronic use	*Treatment resistant:*<br>**Typical** (16.7%)<br>**Atypical** (83.3%)<br>**Mood stabilisers** (38.9%)<br>**Antidepressants** (27.8%)<br>*Responders:*<br>**Typical** (20%) typical<br>**Atypical** (65%) atypical<br>**Aripiprazole** (15%)<br>**Mood stabilisers** (20%)<br>**Antidepressants** (30%)	*Treatment resistant:*<br>**Concomitant drugs** (44%)<br>*Responders:*<br>**Concomitant drugs** (45%)	**Exclusion** (eating disorder, current PTSD, neurological disorder, TBI, reported history of suicide attempt)	N/R	N/R	N/R
110	Miao et al. (2021)	N/R	N/R	**Exclusion** (antipsychotics, antidepressants, or other psychotropics)	**Exclusion** (folic acid or vitamin B12 related drugs within 1 month or immunosuppression past 3 months)	**Exclusion** (other neuropsychiatric condition)	**Exclusion** (diarrhoea or abnormal stool routine examination)	**Exclusion** (chronic diseases e.g., hypertension, heart disease, diabetes)	**Serum folate**
100	Nguyen et al. (2019)	**Smoking**	**Antibiotics**: 28% within past year	**Anti-psychotics** (84%)	N/R	**Exclusion** (any other current major DSM-IV-TR Axis I diagnoses; diagnosis of dementia, intellectual disability disorder, or a major neurological disorder)	N/R	**Diabetes** (36%)<br>**Hypertension** (76%)<br>Report no exclusion of chronic diseases due to high co-morbidity of SZ with such conditions	N/R
107	Nguyen et al. (2021)	**Smoking**	**Antibiotics**: 25% within past year	**Anti-psychotics** (83%)	N/R	**Exclusion** (any other current major DSM-IV-TR Axis I diagnoses; diagnosis of dementia, intellectual disability disorder, or a major neurological disorder)	N/R	**Diabetes** (50%)<br>**Heart disease** (17%)<br>**Hypertension** (67%)<br>Report no exclusion of chronic diseases due to high co-morbidity of SZ with such conditions	**IL-6**<br>**TNF-a**<br>**IL-10**<br>**CRP**
101	Pan et al. (2020)	**Smoking** (exclusion)<br>**Alcohol use** (exclusion)	**Probiotics**: Exclusion 2 weeks before faecal sample<br>**Antibiotics**: Exclusion 2 weeks before faecal sample<br>**Prebiotics**: Exclusion 2 weeks before faecal sample	**History of psychiatric medication** (90% yes; participants were asked 'have you ever received psychiatric medication?')	**Exclusion** (corticosteroids, cytokines)	**Exclusion**	**Exclusion** (IBD; gastrointestinal surgery, gastroscopy, colonoscopy, or gastrointestinal barium in the past 5 years; Diarrhea in the past two weeks)	**Exclusion** (combined with chronic physical diseases such as cancer, hypertension, diabetes, autoimmune diseases, inflammatory bowel diseases, etc.)	N/R
102	Shen et al. (2018)	**Smoking**<br>**Alcohol use**<br>**Diet** (exclusion significantly changing dietary habits)	**Probiotics**: Exclusion past 6 months<br>**Antibiotics**: Exclusion past 6 months	**Anti-psychotics** (100%)	**Exclusion** (drug-use in past 6 months including antibiotics, glucocorticoids, cytokines, large doses of probiotics and biological agents)	**Exclusion** (schizoaffective and other schizophrenia spectrum disorders)	**Exclusion** (IBD, diarrhoea past 3 months; gastroscopy, colonoscopy, or barium meal in past 6 months; major GI surgery in past 5 years)	**Exclusion** (chronic disease or disease that may affect the stability of gut microbiota, such as hypertension, diabetes, immunodeficiency, autoimmune diseases, cancer)	N/R
103	Xu et al. (2020)	**Diet** (FFQ - compared major macro- and micro-nutrient intakes between cases and controls)<br>**Sleep** (collected using a general diet and lifestyle habits questionnaire)<br>**Smoking**<br>**Physical activity**	**Antibiotics**: Exclusion previous 2 weeks	N/R	**Exclusion** (drug use for gastrointestinal diseases in the 2 weeks prior to starting the study)	N/R	N/R	**Exclusion** (genetic metabolic diseases, brain injury, acute physical illness)	**Stool IgA**<br>**Glutamate synthase activity**

Ref	Author (Year)	Lifestyle factors/ behaviours	Use of biotics	Psychiatric medications	Non-psychiatric medications	Psychiatric co-morbidity	Gastrointestinal co-morbidity	Other physical co-morbidities	Biomarkers
111	Yuan et al. (2021)	**Smoking**	**Antibiotics**: Exclusion month before participation	**Exclusion**	**Exclusion** (anti-inflammatory agents in the previous month)	**Exclusion** (neurological diseases, mental retardation, or other psychiatric diseases)	**Exclusion** (gastrointestinal diseases)	**Exclusion** (diagnosis of autoimmune diseases, heart diseases, hepato-biliary and gastrointestinal diseases, blood diseases, diabetes (type I and type II))	**hs-CRP**<br>**HCY**
104	Zhang et al. (2020)	**Smoking**	**Probiotics**: Exclusion 2 months before faecal sample<br>**Antibiotics**: Exclusion 2 months before faecal sample<br>**Prebiotics**: Exclusion 2 months before faecal sample<br>**Synbiotics**: Exclusion 2 months before faecal sample	**Drug-naïve**	N/R	**Exclusion** (alcohol or drug abuse during the past year)	N/R	**Exclusion** (history of systemic diseases or co-morbidities; known active bacterial, fungal, or viral infections)	N/R
105	Zheng et al. (2019)	N/R	**Probiotics**: Exclusion month before faecal sample<br>**Antibiotics**: Exclusion month before faecal sample<br>**Prebiotics**: Exclusion month before faecal sample	**Anti-psychotics** (92.1%)<br>**Unmedicated** (7.9%)	N/R	**Exclusion**	N/R	**Exclusion** (any physical disorders)	N/R
106	Zhu et al. (2020)	**Smoking**<br>**Diet** (collection method not reported; matched cases and controls based on diet habit, collecting data on staple food structure, and frequency of consumption of vegetables, livestock meat, poultry, nutritional supplements, and drinking yoghurt or probiotic drinks)	**Antibiotics**: Exclusion previous 30 days	**Treatment-free** (first episode - less than 100 mg chlorpromazine equivalents in the last 2 weeks)	**Exclusion** (prescribed medications that could affect the central nervous, endocrine, or immune system; drug use for GI disorders 2 weeks prior to study)	**Exclusion**	**Exclusion** (acute digestive tract disorder during the last 30 days)	**Exclusion** (current physical illness such as diabetes, heart disease, thyroid disease, autoimmune disease or any recent infections)	**Dopamine**<br>**Serotonin**<br>**GABA**
112	Zhu et al. (2021)	**Diet** (Exclusion diet change significantly in the past 6 months)	**Antibiotics**: Exclusion 3 months before study<br>**Probiotics**: Exclusion 3 months before study	*Acute*:<br>**Exclusion** (antipsychotic treatment; administration of electroconvulsive therapy without convulsions before enrolment)<br>*Remitted*:<br>**Yes** (received only second-generation anti-psychotic, such as risperidone, quetiapine, and aripiprazole; exclusion administration of electroconvulsive therapy without convulsions before enrolment)	**Exclusion** (corticosteroids or other immune preparations in the past 3 months)	**Exclusion** (a history of craniocerebral trauma, organic cerebral diseases, physical diseases, or other mental disorders; a history of alcohol or other substance use)	**Exclusion** (history of digestive tract diseases, abdominal surgery, or intestinal infection in the past 3 months)	**Exclusion** (history of diabetes, hypertension, dyslipidemia, endocrine disease, or known medical conditions that might affect metabolism)	N/R

N/R = Not reported; N/A = Not applicable; FFQ = Food Frequency Questionnaire; PSQI = Pittsburgh Sleep Quality Index; IPAQ = International Physical Activity Questionnaire; FEP = First episode; FSCZ = First episode schizophrenia; TSCZ = Treated schizophrenia; MDD = Major depressive disorder; BD = Bipolar disorder; SZ = Schizophrenia; SSRIs = Selective serotonin reuptake inhibitors; SNRIs = Serotonin-norepinephrine reuptake inhibitors; A-MDD = Active major depressive disorder; R-MDD = Responded major depressive disorder; NSAIDs = Non-steroidal anti-inflammatory drugs; DSM = Diagnostic and Statistical Manual of Mental Disorders; IBS = Irritable Bowel Syndrome; IBD = Inflammatory Bowel Disease; GI = Gastrointestinal; GSRS = Gastrointestinal Symptom Rating Scale; CVD = Cardiovascular disease; BDNF = Brain-derived neurotrophic factor; IL = Interleukin; TNF = Tumour Necrosis Factor; CRP = C-reactive protein; KYN = Kynurenine; TRP = Tryptophan; AUCg = Area under the curve with respect to ground; LBP = Lipopolysaccharide binding protein; IFN = Interferon; MCP = Monocyte chemoattractant protein; HDL = High-density lipoprotein; LDL = Low-density lipoprotein; IgA = Immunoglobulin A; GABA = gamma-y-aminobutyric acid.

*Exclusion criteria other mental disorders however had anxiety disorders in the cohort

Table S6. Additional covariate considerations and adjustments

Ref	Author (Year)	Adjusted for alpha-diversity	Adjusted for beta-diversity	Adjusted for differential abundance	Associations between gut microbiota composition and covariates (multivariable)	Associations between gut microbiota composition and covariates (bivariable)
**Major depressive disorder (MDD)**						
87	Bai et al. (2021)	No	No	No	No	**Yes** - correlations between differentially abundant taxa (metabolites*)
70	Chen et al. (2018)	No	No	No	No	**Yes** - correlations with differentially abundant taxa (age*, BMI*, HDRS*)
69	Chen et al. (2020)	No	No	No	**Yes** - differential abundance (age*, medication)	No
85	Chen et al. (2021)	No	No	No	**Yes** - differential abundance (psychiatric symptoms*, family history*)	**Yes** - correlations with beta-diversity (age, area, BMI, character, marital status, family history*, time of illness, illness duration, sleep, food intake, fasting blood glucose, blood pressure, thyroid, functioning, HDRS, HAMA, PANSS*)
71	Chung et al. (2019)	No	No	**Yes** (sequencing platform, fat intake)	No	**Yes** - correlations with differentially abundant taxa (HDRS*)
88	Dong et al. (2021)	No	No	No	No	**Yes** - correlations between differentially abundant taxa (hormones, symptom severity)
72	Huang et al. (2018)	No	No	No	No	No
73	Jiang et al. (2015)	No	No	No	No	**Yes** - correlations with differentially abundant taxa (BDNF*, serum inflammatory markers, HDRS*, MADRS*)
74	Kelly et al. (2016)	No	No	No	No	No
75	Lai et al. (2019)	No	No	No	**Yes** - alpha- and beta-diversity (medication)	No
76	Lin et al. (2017)	No	No	No	No	**Yes** - correlations with differentially abundant taxa (HDRS*)
77	Liu et al. (2020)	No	No	No	**Yes** - differential abundance (psychotropic medication use*, PROMIS*)	**Yes** - correlations with alpha-diversity (psychotropic medication use*, PROMIS)
78	Liu et al. (2016)	No	No	No	No	No
79	Mason et al. (2020)	**Yes** (gender, age, race, BMI)	No	**Yes** (gender, age, race, BMI)	**Yes** - beta-diversity and differential abundance (QIDS-SR, GAD-7, SHAPS*)	No
80	Naseribafrouei et al. (2014)	No	No	No	No	No

Ref	Author (Year)	Adjusted for alpha-diversity	Adjusted for beta-diversity	Adjusted for differential abundance	Associations between gut microbiota composition and covariates (multivariable)	Associations between gut microbiota composition and covariates (bivariable)
81	Rong et al. (2019)	No	No	No	**Yes** - differential abundance<br>(medication)	**Yes** - correlations with alpha-diversity<br>(age, BMI*, age of onset, total disease course*, episode duration, episodes of disease, total treatment time, MDQ, HCL-33, HDRS, HAMA*)
89	Shen et al. (2021)	No	No	No	No	No
82	Stevens et al. (2020)	No	No	No	**Yes** - beta-diversity<br>(antidepressants, sex)	No
86	Yang et al. (2020)	No	No	No	No	**Yes** - correlations with differentially abundant taxa<br>(HDRS*, QIDS*)
90	Ye et al. (2021)	No	No	No	No	No
91	Zhang et al. (2021)	No	No	No	**Yes** - differential abundance<br>(sleep quality, age, gender, BMI, HDRS score)	No
84	Zheng et al. (2016)	No	No	No	**Yes** - beta-diversity<br>(age, sex, BMI, smoking status, antidepressant use)	No
83	Zheng et al. (2020)	No	No	No	**Yes** - beta-diversity<br>(BMI)	**Yes** - correlations with differentially abundant taxa<br>(HDRS*)
92	Zheng et al. (2021)	No	No	No	No	No
**Bipolar disorder (BD)**						
93	Coello et al. (2019)	No	No	**Yes** - differential abundance<br>(sex, age, current smoker, waist circumference, physical activity, illness duration, BD type, HDRS, YMRS, current affective state, psychotropic medication use)	No	No
94	Evans et al. (2017)	No	No	**Yes** - differential abundance<br>(age, sex, BMI)	No	**Yes** - correlations with differentially abundant taxa<br>(PCS*, MCS, PSQI*, GAD-7, PHQ-9*, ASRM)
95	Hu et al. (2019)	No	No	No	**Yes** - beta-diversity<br>(age*)	**Yes** - correlations with differentially abundant taxa<br>(BMI*, duration of illness*, MADRS*)
97	Lai et al. (2021)	No	No	No	No	No
96	McIntyre et al. (2019)	No	No	No	**Yes** - beta-diversity<br>(diet, BD type)	No
81	Rong et al. (2019)	No	No	No	**Yes** - differential abundance<br>(medication)	**Yes** - correlations with alpha-diversity<br>(age, BMI, age of onset, total disease course*, episode duration, episodes of disease*, total treatment time, MDQ, HCL-33, HDRS, HAMA*)
83	Zheng et al. (2020)	No	No	No	**Yes** - beta-diversity<br>(BMI, location, BD type, age)	**Yes** - correlations with differentially abundant taxa<br>(HDRS*)

Ref	Author (Year)	Adjusted for alpha-diversity	Adjusted for beta-diversity	Adjusted for differential abundance	Associations between gut microbiota composition and covariates (multivariable)	Associations between gut microbiota composition and covariates (bivariable)
**Schizophrenia (SZ)**						
98	Li et al. (2020)	No	No	No	No	**Yes** - correlations with differentially abundant taxa (PANSS*)
108	Li et al. (2021)	No	No	No	No	**Yes** - correlations with alpha-diversity, beta-diversity, and differentially abundant taxa (MRI*)
99	Ma et al. (2020)	No	No	No	No	**Yes** - correlation with differentially abundant taxa (brain volumes*)
109	Manchia et al. (2021)	No	No	**Yes** (age, gender, BMI - specifically did not consider lifestyle variables as they were intrinsically related to the healthy control profile)	No	No
110	Miao et al. (2021)	No	No	No	**Yes** - differential abundance of Bifidobacterium (folic acid, PANSS*)	No
100	Nguyen et al. (2019)	No	No	No	**Yes** - beta-diversity (sex*, smoking status*, illness duration, diabetes, heart disease, hypertension)	**Yes** - correlations with alpha-diversity (age, age of disease onset*)<br>**Yes** - correlation with differentially abundant taxa (illness duration, depressive symptoms, negative symptoms, mental wellbeing, CHD risk)
107	Nguyen et al. (2021)	No	No	No	**Yes** - beta-diversity (sex*, BMI category*, smoking status*, current antipsychotic medication use)	No
101	Pan et al. (2020)	No	No	No	No	**Yes** - correlations with differentially abundant taxa (PANSS*)
102	Shen et al. (2018)	No	No	No	No	No
103	Xu et al. (2020)	No	No	No	No	**Yes** - correlation with differentially abundant taxa (GOGAT*, IgA*, ME*)
111	Yuan et al. (2021)	**Yes** (age, gender, education, disease duration, smoking status, and BMI)	**Yes** (age, gender, education, disease duration, smoking status, and BMI)	**Yes** (age, gender, education, disease duration, smoking status, and BMI)	**Yes** - differential abundance (PANSS*, change in PANSS*)	No
104	Zhang et al. (2020)	No	No	No	No	**Yes** - correlation with differentially abundant taxa (mycobiota*)
105	Zheng et al. (2019)	No	No	No	**Yes** - beta-diversity (sex, antipsychotic use, antipsychotic type)	**Yes** - correlation with differentially abundant taxa (PANSS*)
106	Zhu et al. (2020)	No	No	**Yes** (diet, BMI, age, sex)	No	**Yes** - correlation with differentially abundant taxa (MCCB, PANSS, neurotransmitter, overall diet)

Ref	Author (Year)	Adjusted for alpha-diversity	Adjusted for beta-diversity	Adjusted for differential abundance	Associations between gut microbiota composition and covariates (multivariable)	Associations between gut microbiota composition and covariates (bivariable)
112	Zhu et al. (2021)	No	No	No	No	**Yes** - correlation with differentially abundant taxa (PANSS*)

*** Significant differences in composition associated with the specific covariate, or correlations identified between covariate and gut microbiota composition**
Multivariable associations estimated from regression models e.g., linear regression, logistic regression.
Bivariable associations estimated from Spearman or Pearson correlations.

Table S7. Description of gut microbiota collection and processing methods across studies

Ref	Author (Year)	Stool sample collection	Stool sample transport	Stool sample storage	DNA extraction kit	Sequencing type	Sequencing technology	Sequencing region	Bioinformatics	Mapping database	OTUs/ASVs	Rarefied	Normalised
**Major depressive disorder (MDD)**													
87	Bai et al. (2021)	N/R	N/R	N/R	N/R	16S rRNA sequencing	N/R	N/R	N/R	Ribosomal Database Project	OTUs (97%)	No	No
70	Chen et al. (2018)	N/R	N/R	At -80ºC	PowerSoil Kit with MoBio lysis buffer and garnet bead tubes	16S rRNA sequencing	Roche 454 sequencing	V3-V5	Mothur	Ribosomal Database Project	OTUs	No	No
69	Chen et al. (2020)	N/R	N/R	N/R	PowerSoil Kit with MoBio lysis buffer and garnet bead tubes	16S rRNA sequencing	Roche 454 sequencing	V3-V5	Mothur	Ribosomal Database Project	OTUs (97%)	No	No
85	Chen et al. (2021)	Fresh	N/R	At -80ºC	Qiagen QIAamp DNA Stool Mini Kit	16S rRNA sequencing Shotgun metagenomics	Illumina Miseq Illumina NovaSeq	V3-V4 Whole-genome	USEARCH; UPARSE; Mothur; QIIME; MegaHit; MetaGeneMark; CD-HIT; Bowtie2; eXperss; DIAMOND	Ribosomal Database Project	OTUs (97%) mOTUs	No	Yes (for beta-diversity)
71	Chung et al. (2019)	Fresh	Transported at 4ºC	At -80ºC	QIAamp DNA stool mini kit OR phenol-chloroform extraction method	16S rRNA sequencing	Illumina Miseq and MiniSeq platforms	V3-V4 and V4	PEAR, QIIME,	Greengenes (May 2013 version)	OTUs (97%)	No	No
88	Dong et al. (2021)	N/R	Not required - immediately frozen	At -80ºC	Qiagen QIAamp DNA Stool Mini Kit	16S rRNA sequencing	Illumina Miseq	V3-V4	UPARSE; UCHIME	Ribosomal Database Project Classifier using SILVA	OTUs (97%)	Yes	No
72	Huang et al. (2018)	Fresh	N/R	At -80ºC	PowerSoil DNA Kit	16S rRNA sequencing	Illumina Miseq	V3-V4	QIIME, UCHIME, UCLUST	Greengenes	OTUs (97%)	Yes	No
73	Jiang et al. (2015)	Fresh	Icebox for 15 minute travel	At -80ºC	QIAamp DNA stool Mini Kit with glass-bead steps	16S rRNA sequencing	Roche 454 sequencing	V1-V3	Mothur, ChimeraSlayer	Ribosomal Database Project	OTUs (97%)	No	No
74	Kelly et al. (2016)	Fresh with AnaeroGen Compact Oxoid sachet	N/R	At -80ºC for n=21 samples	QIAamp DNA Stool Mini Kit (21 frozen, 43 fresh)	16s rRNA sequencing	Illumina Miseq	N/R	FLASH, QIIME, USEARCH, PyNAST,	SILVA	OTUs (97%)	Yes	Yes
75	Lai et al. (2019)	N/R	N/R	At -80ºC	StoolGen DNA kit	Shotgun metagenomics	Illumina Hiseq2500	Whole genome	MEGAN5	NCBI genome database	N/A	No	No
76	Lin et al. (2017)	Fresh	Not required - immediately frozen	At -70ºC	Tiagen DNA Stool Mini Kit	16S rRNA sequencing	Illumina Hiseq2+H15500	V3-V4	Mothur	SILVA	OTUs (97%)	No	No
77	Liu et al. (2016)	Fresh	Not required - immediately frozen	At -80ºC	PowerSoil DNA Isolation Kit	16S rRNA sequencing	Roche 454 sequencing	V1-V3	Mothur	Ribosomal Database Project	OTUs	No	No

Ref	Author (Year)	Stool sample collection	Stool sample transport	Stool sample storage	DNA extraction kit	Sequencing type	Sequencing technology	Sequencing region	Bioinformatics	Mapping database	OTUs/ASVs	Rarefied	Normalised
78	Liu et al. (2020)	OMNIgene Gut	N/R (in stabiliser)	At -80ºC	ZymoBIOMICS 96 DNA kit	16S rRNA sequencing	Illumina Miseq	V4	QIIME2	SILVA	ASVs	No	No
79	Mason et al. (2020)	N/R	N/R	N/R	STE/Phenol/chloroform/isoamyl extraction method	16S rRNA sequencing	Roche 454 sequencing	V4	QIIME, UCLUST	SILVA	OTUs (97%)	No	No
80	Naseribafrouei et al. (2014)	Fresh then STAR buffer	At below 0ºC	At -20ºC in home freezer, then at -80ºC in lab	Mag mini kit	16s rRNA sequencing	Illumina Miseq	N/R	QIIME, UCLUST	Ribosomal Database Project	OTUs (99%)	No	No
81	Rong et al. (2019)	N/R	N/R	At -80ºC	StoolGen DNA kit	Shotgun metagenomics	Illumina Hiseq2500	Whole genome	VEGAN	N/R	N/A	No	No
89	Stevens et al. (2020)	OMNIgene Gut	N/R (In stabiliser)	At -80ºC	E.Z.N.A Stool Extraction Kit	16S rRNA sequencing	Illumina Miseq	V3-V4	DADA2, ALDEx2, PIME	SILVA	ASVs (filtered)	No	Yes
82	Shen et al. (2021)	Fresh	Immediately in liquid nitrogen	At -80ºC	PowerSoil DNA Isolation Kit	16S rRNA sequencing	Illumina Hiseq2500	V3-V4	Mothur, QIIME, USEARCH, UNITE (fungi)	SILVA	OTUs (97%)	No	No
86	Yang et al. (2020)	N/R	N/R	N/R	E.Z.N.A. Soil DNA Kit	Shotgun metagenomics	Illumina NovaSeq	Whole genome	Sickle; MegaHIT; Metagene; Diamond	NCBI genome database	N/A	No	No
90	Ye et al. (2021)	Fresh	N/R	At -80ºC	Power Soil DNA Isolation Kit	16S rRNA sequencing	Illumina Hiseq2500	V3-V4	FLASH, UCHIME, UPARSE	Ribosomal Database Project	OTUs (97%)	No	No
91	Zhang et al. (2021)	Fresh	N/R (in stabiliser)	At -80ºC	N/R	16S rRNA sequencing	Illumina NovaSeq	V4-V5	Mothur, UPARSE	N/R	OTUs (97%)	No	No
84	Zheng et al. (2016)	Fresh	Not required - immediately frozen	At -80ºC	PowerSoil Kit	16S rRNA sequencing	Roche 454 sequencing	V3-V5	Mothur; QIIME; PyNAST	Ribosomal Database Project	OTUs (97%)	No	No
83	Zheng et al. (2020)	N/R	N/R	N/R	OMEGA-soil DNA Kit	16S rRNA sequencing	Illumina Miseq	V3-V4	UPARSE	Ribosomal Database Project	OTUs (97%)	No	No
92	Zheng et al. (2021)	Fresh	N/R (in stabiliser)	At -80ºC	QIAamp DNA Stool Mini Kit	16S rRNA sequencing	Illumina Miseq	V4-V5	Mothur, UPARSE	N/R	OTUs (97%)	No	No
**Bipolar disorder (BD)**													
93	Coello et al. (2019)	OMNIgene Gut	Pre-paid mailer within 14 days	At -80ºC	NucleoSpin 96 soil kit vacuum protocol	16S rRNA sequencing	Illumina MiSeq	V3-V4	USEARCH, Mothur, in-house scripts, SINTAX	Ribosomal Database Project	OTUs (97%)	Yes	No
94	Evans et al. (2017)	OMNIgene Gut	Pre-paid mailer	At -80ºC	PowerMag Microbiome RNA/DNA Isolation Kit	16S rRNA sequencing	Illumina MiSeq	V4	Mothur	SILVA	OTUs (97%)	No	No
95	Hu et al. (2019)	Fresh	N/R	At -80ºC	PSP Spin Stool DNA Plus Kit	16S rRNA sequencing	Illumina MiSeq	V3-V4	Flash, UCHIME	Ribosomal Database Project	OTUs (97%)	No	No
97	Lai et al. (2021)	Fresh	N/R	At -80ºC	StoolGen DNA kit	Shotgun metagenomics	Illumina Hiseq2500	Whole genome	MEGAN5	N/R	N/A	No	No
96	McIntyre et al. (2019)	Fresh	N/R	Frozen at home	Anaerobically. MagMax robot	16S rRNA sequencing (not explicitly stated)	Illumina MiSeq	N/R	Sickle, Cutadapt, PandaSeq, QIIME	Greengenes	OTUs (97%)	Yes	No
81	Rong et al. (2019)	N/R	N/R	At -80ºC	StoolGen DNA kit	Shotgun metagenomics	Illumina Hiseq2500	Whole genome	VEGAN	N/R	N/A	No	No

Ref	Author (Year)	Stool sample collection	Stool sample transport	Stool sample storage	DNA extraction kit	Sequencing type	Sequencing technology	Sequencing region	Bioinformatics	Mapping database	OTUs/ASVs	Rarefied	Normalised
83	Zheng et al. (2020)	N/R	N/R	N/R	OMEGA-soil DNA Kit	16S rRNA sequencing	Illumina Miseq	V3-V4	UPARSE	Ribosomal Database Project	OTUs (97%)	No	No
**Schizophrenia (SZ)**													
98	Li et al. (2020)	Fresh	N/R	At -80ºC	PowerSoil DNA Isolation Kit	16S rRNA sequencing	Illumina MiSeq	V4	QIIME2; DADA2;	Greengenes	N/R	Yes	No
108	Li et al. (2021)	Fresh (fasted)	N/R	At -80ºC	MOBIO PowerSoil DNA Isolation Kit	16S rRNA sequencing	Illumina Miseq	V4	QIIME, DADA2	Greengenes	N/R	No	Yes
99	Ma et al. (2020)	Fresh	Not required - immediately frozen	At -80ºC	QIAamp DNA mini kit	16S rRNA sequencing	Illumina MiSeq	V4	In-house scripts, UCHIME, UPARSE, QIIME	Greengenes	OTUs (97%)	No	No
109	Manchia et al. (2021)	Fresh	N/R (in stabiliser)	N/R	QIAamp DNA Stool Mini Kit	16S rRNA sequencing	Illumina Miseq	V3-V4	N/R	Custom database (based on NCBI)	N/R	No	Yes
110	Miao et al. (2021)	Fresh	N/R	At -80ºC	QIAamp FAST DNA kit	16S rRNA sequencing	IonS5TMXL sequencer platform	V3-V4	QIIME	SILVA	OTUs (97%)	No	No
100	Nguyen et al. (2019)	BD Swube Dual Swab Collection System	Returned via mail	At -80ºC	MoBio PowerMag Soil DNA Isolation Kit	16S rRNA sequencing	Illumina MiSeq	V4	QIIME 2, Deblur, UCLUST, Calour	Greengenes	sOTUs	Yes	No
107	Nguyen et al. (2021)	BD Swube Dual Swab Collection System	Returned via mail	At -80ºC	Qiagen MagAttract PowerSoil DNA kit	16S rRNA sequencing	Illumina Miseq and HiSeq	V4	QIIME2; Deblur; UCLUST	Greengenes	ASVs	Yes	No
101	Pan et al. (2020)	Fresh	Polyethylene box with ice blocks	At -80ºC	E.Z.N.A. ® soil kit	16S rRNA sequencing	Illumina MiSeq	V3-V4	USEARCH; QIIME	Ribosomal Database Project	OTUs (97%)	No	No
102	Shen et al. (2018)	Fresh	N/R	At -80ºC	PowerSoil DNA kit	16S rRNA sequencing	Illumina Hiseq 2500	V3-V4	QIIME, UCLUST	Greengenes	OTUs (97%)	Yes	No
103	Xu et al. (2020)	Fresh - sample taken from middle of faecal sample	N/R	At -20ºC in home freezer, then at -80ºC in lab within 48 hours	StoolGen fecal DNA extraction kit	*Discovery set:* Shotgun metagenomics *Validation set:* 16s rRNA sequencing	*Discovery set:* Illumina Hiseq4000 *Validation set:* Illumina HiSeq2500	*Discovery set:* Whole genome *Validation set:* V4	*Discovery set:* Bowtie, MEGAN5 *Validation set:* Mothur, UCHIME, QIIME, UPARSE, USEARCH, MUSCLE	*Discovery set:* NCBI genome databases *Validation set:* SILVA	*Discovery set:* N/A *Validation set:* OTUs (97%)	No	No
111	Yuan et al. (2021)	Fresh	Not required - immediately frozen	At -80ºC	Cetyltrimethylammonium Ammonium Bromide CTAB/SDS method	16S rRNA sequencing	IonS5TMXL sequencer platform	V3-V4	UCHIME, UPARSE, Mothur	SILVA	OTUs (97%)	No	No
104	Zhang et al. (2020)	Fresh	In an icebox	At -80ºC	FastDNATM SPIN Kit for Feces	16S rRNA sequencing	Illumina MiSeq	N/R	QIIME; ChimeraSlayer	N/R	OTUs (97%)	No	No
105	Zheng et al. (2019)	Fresh	Not required - immediately frozen	At -80ºC	QIAamp DNA Stool Mini Kit	16S rRNA sequencing	Illumina MiSeq	V3-V4	QIIME; UCHIME; USEARCH	SILVA	OTUs (97%)	No	No
106	Zhu et al. (2020)	N/R	N/R	N/R	N/R	Shotgun metagenomics	Illumina (type not specified)	Whole genome	N/R	SOAP de novo assembly	mOTUs	No	No
112	Zhu et al. (2021)	Fresh	N/R	At -80ºC	Qiagen QIAamp Fast DNA stool MINI Kit	16S rRNA sequencing	Illumina Hiseq2500	V3-V4	N/R	N/R	N/R	No	No

N/R = Not reported; N/A = Not applicable
16S = 16S rRNA sequencing; ASV = Amplicon sequence variant; OTU = Operational taxonomic unit.

Table S8. Brief overview of gut microbiota analyses across studies

Ref	Author (Year)	Relative/absolute abundance?	Adjusted for multiple comparisons?	LEfSe	PICRUST	KEGG	ROC/AUC	Random Forest Algorithm	Rarefaction curve analysis
**Major depressive disorder (MDD)**									
87	Bai et al. (2021)	**Relative**	No	**Yes**	**Yes**	No	**Yes**	No	No
70	Chen et al. (2018)	**Relative**	No	**Yes** (>2.0)	No	No	No	**Yes**	No
69	Chen et al. (2020)	**Relative**	No	**Yes** (>2.0)	No	No	**Yes**	**Yes**	No
85	Chen et al. (2021)	**Relative**	**Yes**	**Yes** (>2.4)	No	**Yes**	**Yes**	**Yes**	No
71	Chung et al. (2019)	**Relative**	Unclear	No	**Yes**	No	No	No	No
88	Dong et al. (2021)	**Relative**	**Yes**	No	**Yes**	No	No	No	No
72	Huang et al. (2018)	**Relative**	No	**Yes** (>2.0)	**Yes**	No	No	No	No
73	Jiang et al. (2015)	**Relative**	No	**Yes** (>2.0)	No	No	No	No	**Yes**
74	Kelly et al. (2016)	**Relative**	**Yes**	No	No	No	No	No	No
75	Lai et al. (2019)	**Relative**	**Yes**	**Yes** (>3.0)	No	**Yes**	**Yes**	**Yes**	No
76	Lin et al. (2017)	Not specified	No	No	No	No	No	No	No
77	Liu et al. (2020)	**Relative**	No	**Yes** (>2.0)	**Yes**	No	No	No	No
78	Liu et al. (2016)	**Relative**	No	No	No	No	No	No	**Yes**
79	Mason et al. (2020)	Not specified	**Yes**	No	No	No	No	No	No
80	Naseribafrouei et al. (2014)	Not specified	**Yes**	No	No	No	**Yes**	No	No
81	Rong et al. (2019)	**Relative**	**Yes**	No	No	**Yes**	No	No	No
89	Shen et al. (2021)	**Relative**	No	**Yes** (>4.0)	**Yes**	No	No	No	No
82	Stevens et al. (2020)	**Relative**	No	No	**Yes**	No	No	**Yes**	No
86	Yang et al. (2020)	**Relative**	No	**Yes** (>2.5)	No	**Yes**	**Yes**	**Yes**	No
90	Ye et al. (2021)	**Relative**	No	**Yes** (>3.5)	No	No	No	No	**Yes**
91	Zhang et al. (2021)	**Relative**	**Yes**	**Yes** (>2.0)	No	No	No	No	**Yes**
84	Zheng et al. (2016)	**Relative**	No	No	No	No	No	**Yes**	No
83	Zheng et al. (2020)	**Relative**	Unclear	**Yes** (>2.5)	No	No	**Yes**	**Yes**	No
92	Zheng et al. (2021)	**Relative**	No	**Yes**	No	No	No	No	**Yes**
**Bipolar disorders (BD)**									
93	Coello et al. (2019)	Relative	**Yes**	No	No	No	No	No	No
94	Evans et al. (2017)	Relative	**Yes**	No	No	No	No	No	No
95	Hu et al. (2019)	Relative	No	**Yes** (>2.0)	**Yes**	No	**Yes**	**Yes**	No
97	Lai et al. (2021)	Relative	**Yes**	**Yes** (LDA>3.0)	No	**Yes**	**Yes**	**Yes**	No

Ref	Author (Year)	Relative/absolute abundance?	Adjusted for multiple comparisons?	LEfSe	PICRUST	KEGG	ROC/AUC	Random Forest Algorithm	Rarefaction curve analysis
96	McIntyre et al. (2019)	Relative	**Yes**	No	No	No	No	No	No
81	Rong et al. (2019)	Relative	**Yes**	No	No	**Yes**	No	No	No
83	Zheng et al. (2020)	Relative	Unclear	**Yes** (>2.5)	No	No	**Yes**	**Yes**	No
**Schizophrenia (SZ)**									
98	Li et al. (2020)	Relative	**Yes**	**Yes** (>2.0)	**Yes**	No	No	No	No
108	Li et al. (2021)	Relative	**Yes**	No	No	No	No	No	No
99	Ma et al. (2020)	Relative	**Yes**	No	**Yes**	No	No	No	**Yes**
109	Manchia et al. (2021)	Relative	No	No	No	No	No	No	No
110	Miao et al. (2021)	Relative	No	**Yes** (>4.0)	No	No	No	No	No
100	Nguyen et al. (2019)	Relative	**Yes**	No	No	No	No	No	No
107	Nguyen et al. (2021)	Relative	**Yes**	No	**Yes**	No	**Yes**	**Yes**	No
101	Pan et al. (2020)	Relative	No	**Yes** (>2.0)	**Yes**	No	**Yes**	**Yes**	No
102	Shen et al. (2018)	Relative	No	**Yes** (<2.0)	**Yes**	No	**Yes**	**Yes**	No
103	Xu et al. (2020)	Relative	**Yes**	No	No	No	**Yes**	No	No
111	Yuan et al. (2021)	Relative	**Yes**	**Yes** (>3.0)	No	No	**Yes**	**Yes**	**Yes**
104	Zhang et al. (2020)	Relative	No	**Yes** (>2.0)	No	No	No	No	No
105	Zheng et al. (2019)	Relative	No	**Yes**	No	No	**Yes**	No	**Yes**
106	Zhu et al. (2020)	Relative	**Yes**	No	No	**Yes***	**Yes**	**Yes**	**Yes**
112	Zhu et al. (2021)	Relative	No	**Yes** (>2.0)	No	No	No	No	No

LEfSe = Linear Discriminant Analysis Effect Size; PICRUST = Phylogenetic Investigation of Communities by Reconstruction of Unobserved States; KEGG = Kyoto Encylopedia of Genes and Genomes; ROC = Receiver operating characteristic; AUC = Area under the curve.
*** Used Gut-Brain Modules (GBMs)**

Table S9. Quality Assessment of Reviewed Studies

Ref	Author (Year)	Q1	Q2	Q3	Q4	Q5	Q6	Q7	Q8	Q9	Q10	Q11	Q12	Q13	Q14	Quality Rating	Notes
87	Bai et al. (2021)	Yes	No	Yes	No	Yes	No	No	Yes	No	No	No	N/A	N/A	No	Poor	2. Lack of clarity regarding study population; No time-period for recruitment mentioned.<br><br>4. Lack of clarity regarding recruitment of cases; Inclusion/exclusion criteria not consistent across groups.<br><br>9. Does not clearly report stool sample collection or transport methods; the 16S rRNA sequencing platform and hypervariable region were not clearly reported.<br><br>10. Gut microbiota composition was not measured more than once over time.<br><br>11. Do not clearly report using a validated and consistent tool for psychiatric disorder diagnosis<br><br>14. Collected BMI data and excluded psychotropics, but no consideration of diet; did not adjust for these variables.
70	Chen et al. (2018)[24]	Yes	No	Yes	No	Yes	No	No	Yes	No	No	No	N/A	N/A	No	Poor	2. Lack of clarity regarding study population; No time-period for recruitment mentioned.<br><br>4. Inclusion/exclusion criteria not consistent across groups.<br><br>9. Does not clearly report stool sample collection or transport methods.<br><br>10. Gut microbiota composition was not measured more than once over time.<br><br>11. Used a validated diagnostic tool, however unclear if this was applied uniformly across groups.<br><br>14. Lack of consideration of the pre-specified key potential confounding variables.
69	Chen et al. (2020)[23]	Yes	No	Yes	No	Yes	No	No	Yes	No	No	Yes	N/A	N/A	No	Fair	2. No time-period for recruitment mentioned.<br><br>4. Inclusion/exclusion criteria not consistent across groups.<br><br>9. Does not clearly report stool sample collection, storage, or transport methods.<br><br>10. Gut microbiota composition was not measured more than once over time.<br><br>14. Collected BMI and medication data but did not collect diet data; did not adjust for these variables.

Ref	Author (Year)	Q1	Q2	Q3	Q4	Q5	Q6	Q7	Q8	Q9	Q10	Q11	Q12	Q13	Q14	Quality Rating	Notes
85	Chen et al. (2021)[37]	Yes	No	Yes	Yes	Yes	No	No	Yes	Yes	No	Yes	N/A	N/A	No	Fair	2. No time-period for recruitment mentioned.<br><br>10. Gut microbiota composition was not measured more than once over time.<br><br>14. Matched for age and BMI for metagenomic analyses, however some participants were infrequently taking antidepressants, and this was not adjusted for. Furthermore, diet was not taken into consideration.
71	Chung et al. (2019)[25]	Yes	No	Yes	Yes	No	No	No	Yes	Yes	No	Yes	N/A	N/A	No	Fair	2. Lack of clarity regarding study population; No time-period for recruitment mentioned.<br><br>5. No statistical methods with effect size estimates were reported.<br><br>10. Gut microbiota composition was not measured more than once over time.<br><br>14. Adjusted for fat intake % based on dietary assessment, and also for sequencing platform. However, did not account for antidepressant use or BMI, or measure the use of other psychotropic medications.
93	Coello et al. (2018)[39]	Yes	Yes	Yes	No	Yes	No	No	Yes	Yes	No	Yes	N/A	N/A	No	Fair	4. Inclusion/exclusion criteria not consistent across groups.<br><br>10. Gut microbiota composition was not measured more than once over time.<br><br>14. Some consideration of potential confounding however did not measure/adjust for diet.
88	Dong et al. (2021)	Yes	Yes	Yes	Yes	No	No	No	Yes	Yes	No	No	N/A	N/A	No	Fair	5. No statistical methods with effect size estimates were reported.<br><br>10. Gut microbiota composition was not measured more than once over time.<br><br>11. Do not clearly report using a validated and consistent tool for psychiatric disorder diagnosis.<br><br>14. Some consideration of potential confounding – collected BMI data and excluded obvious dietary preferences and psychotropic use; did not collect specific dietary information or adjust for any of these variables.
94	Evans et al. (2017)[40]	Yes	No	No	No	Yes	No	No	Yes	Yes	No	Yes	N/A	N/A	No	Poor	2. Lack of clarity regarding study population; No time-period for recruitment mentioned.<br><br>3. Less than 50% of eligible persons provided with a stool sample collection kit provided samples for the study.<br><br>4. Inclusion/exclusion criteria were not clearly provided/defined for either group.<br><br>10. Gut microbiota composition was not measured more than once over time.<br><br>14. Some consideration of potential confounding however did not measure/adjust for diet.

Ref	Author (Year)	Q1	Q2	Q3	Q4	Q5	Q6	Q7	Q8	Q9	Q10	Q11	Q12	Q13	Q14	Quality Rating	Notes
95	Hu et al. (2019)[41]	Yes	No	Yes	Yes	Yes	No	No	Yes	Yes	Yes	Yes	N/A	N/A	No	Good	2. Lack of clarity regarding study population; No time-period for recruitment mentioned.<br><br>14. Some consideration of potential confounding however did not measure/adjust for diet.
72	Huang et al. (2018)[26]	Yes	Yes	Yes	Yes	Yes	No	No	Yes	Yes	No	No	N/A	N/A	No	Fair	10. Gut microbiota composition was not measured more than once over time.<br><br>11. Do not clearly report using a validated and consistent tool for psychiatric disorder diagnosis.<br><br>14. No differences in BMI reported at baseline and participants were free from systemic anti-depressant treatment. However, other psychotropics or diet were not measured/adjusted.
73	Jiang et al. (2015)[27]	Yes	Yes	Yes	Yes	Yes	No	No	Yes	Yes	No	Yes	N/A	N/A	No	Good	10. Gut microbiota composition was not measured more than once over time.<br><br>14. Some consideration of potential confounding. Collected BMI and psychotropic data and investigated differences in gut microbiota composition after anti-depressant treatment. However, did not measure/adjust for diet.
74	Kelly et al. (2016)[7]	Yes	No	Yes	Yes	No	No	No	Yes	No	No	No	N/A	N/A	No	Poor	2. No time-period for recruitment mentioned.<br><br>5. No statistical methods with effect size estimates were reported.<br><br>9. Did not clearly report stool sample transport method; the specific 16S rRNA gene sequencing region was not clearly specified.<br><br>10. Gut microbiota composition was not measured more than once over time.<br><br>11. Used a validated diagnostic tool, however unclear if this was applied uniformly across groups.<br><br>14. Some consideration of potential confounding – collected BMI and diet data at baseline. All participants on SSRIs. Did not measure/adjust for other psychotropics.
75	Lai et al. (2019)[28]	Yes	No	Yes	Yes	Yes	No	No	Yes	No	No	Yes	N/A	N/A	No	Fair	2. No time-period for recruitment mentioned.<br><br>9. Did not clearly report stool sample collection or transport method<br><br>10. Gut microbiota composition was not measured more than once over time.<br><br>14. Some consideration of potential confounding – collected baseline BMI and psychotropic data. However, did not measure/adjust for diet.

Ref	Author (Year)	Q1	Q2	Q3	Q4	Q5	Q6	Q7	Q8	Q9	Q10	Q11	Q12	Q13	Q14	Quality Rating	Notes
97	Lai et al. (2021)[43]	Yes	No	Yes	Yes	Yes	No	No	Yes	No	No	Yes	N/A	N/A	No	Fair	2. No time-period for recruitment mentioned.<br><br>9. Did not clearly report stool sample transport or mapping database<br><br>10. Gut microbiota composition was not measured more than once over time.<br><br>14. Some consideration of potential confounding – collected baseline BMI and psychotropic data. However, did not measure/adjust for diet.
98	Li et al. (2020)[44]	Yes	Yes	Yes	No	Yes	No	No	Yes	No	No	Yes	N/A	N/A	No	Fair	4. Inclusion/exclusion criteria not consistent across groups.<br><br>9. Did not clearly report stool sample transport or specify whether they used OTUs or ASVs.<br><br>10. Gut microbiota composition was not measured more than once over time.<br><br>14. Some consideration of potential confounding – collected baseline BMI and psychotropic data. However, did not measure/adjust for diet.
108	Li et al. (2021)	Yes	No	Yes	Yes	No	No	No	Yes	No	No	No	N/A	N/A	No	Poor	2. No time-period for recruitment mentioned.<br><br>5. No statistical methods with effect size estimates were reported.<br><br>9. Did not clearly report stool sample transport or specify whether they used OTUs or ASVs.<br><br>10. Gut microbiota composition was not measured more than once over time.<br><br>11. Possibly used a validated tool for psychiatric disorder diagnosis (SCID) however unclear if this was uniformly applied across groups.<br><br>14. Collected BMI and psychotropic data but did not consider diet; did not adjust for these variables.
76	Lin et al. (2017)[29]	No	No	No	No	No	No	No	Yes	Yes	Yes	No	N/A	N/A	No	Poor	1. Study aims not clearly defined.<br><br>2. Lack of clarity regarding study population; No time-period for recruitment mentioned.<br><br>3. Less than 50% of eligible persons provided samples for the study.<br><br>4. Inclusion/exclusion criteria not consistent across groups.<br><br>5. No statistical methods with effect size estimates were reported.<br><br>11. Do not clearly report using a validated and consistent tool for psychiatric disorder diagnosis.<br><br>14. Collected baseline BMI data and all participants were taking antidepressants. did not measure/adjust for diet or psychotropic use.

Ref	Author (Year)	Q1	Q2	Q3	Q4	Q5	Q6	Q7	Q8	Q9	Q10	Q11	Q12	Q13	Q14	Quality Rating	Notes
78	Liu et al. (2016)[31]	Yes	No	Yes	Yes	No	No	No	Yes	Yes	No	Yes	N/A	N/A	No	Fair	2. Lack of clarity regarding study population; No time-period for recruitment mentioned.<br><br>5. No statistical methods with effect size estimates were reported.<br><br>10. Gut microbiota composition was not measured more than once over time.<br><br>14. Collected baseline BMI data and excluded those using psychotropic medications. Did not measure/adjust for diet.
77	Liu et al. (2020)[30]	Yes	Yes	Yes	Yes	Yes	No	No	Yes	Yes	No	Yes	N/A	N/A	No	Good	10. Gut microbiota composition was not measured more than once over time.<br><br>14. Collected psychotropic medication use data. Did not measure/adjust for BMI or diet.
99	Ma et al. (2020)[45]	Yes	No	Yes	Yes	No	No	No	Yes	Yes	No	Yes	N/A	N/A	No	Fair	2. Lack of clarity regarding study population; No time-period for recruitment mentioned.<br><br>5. No statistical methods with effect size estimates were reported.<br><br>10. Gut microbiota composition was not measured more than once over time.<br><br>14. Lack of consideration of key potentially confounding variables.
109	Manchia et al. (2021)	Yes	No	Yes	Yes	No	No	No	Yes	No	No	No	N/A	N/A	No	Poor	2. No time-period for recruitment mentioned.<br><br>5. No statistical methods with effect size estimates were reported.<br><br>9. Did not clearly report on bioinformatic methods or specify if they used OTUs or ASVs<br><br>10. Gut microbiota composition was not measured more than once over time.<br><br>11. Used a validated diagnostic tool, however unclear if this was applied uniformly across groups.<br><br>14. Adjusted for age, gender, and BMI; collected diet and psychotropic data but did not adjust for these.
79	Mason et al. (2020)[32]	Yes	No	Yes	Yes	No	No	No	Yes	No	No	Yes	N/A	N/A	No	Fair	2. Lack of clarity regarding study population; No time-period for recruitment mentioned.<br><br>5. No statistical methods with effect size estimates were reported.<br><br>9. Did not clearly report stool sample collection, transport, or storage.<br><br>10. Gut microbiota composition was not measured more than once over time.<br><br>14. Some consideration of potential confounding; however, did not measure/adjust for diet.

Ref	Author (Year)	Q1	Q2	Q3	Q4	Q5	Q6	Q7	Q8	Q9	Q10	Q11	Q12	Q13	Q14	Quality Rating	Notes
96	McIntyre et al. (2019)[42]	Yes	No	Yes	Yes	No	No	No	Yes	No	No	No	N/A	N/A	No	Poor	2. No time-period for recruitment mentioned.<br><br>5. No statistical methods with effect size estimates were reported.<br><br>9. Did not clearly report stool sample transport, or 16S rRNA gene sequencing region<br><br>10. Gut microbiota composition was not measured more than once over time.<br><br>11. Used a validated diagnostic tool, however unclear if this was applied uniformly across groups.<br><br>14. Some consideration of potential confounding – collected baseline BMI and dietary data. However, did not measure/adjust for psychotropic medication.
110	Miao et al. (2021)	Yes	No	Yes	No	Yes	No	No	Yes	Yes	No	No	N/A	N/A	No	Poor	2. Lack of clarity regarding control study population.<br><br>4. Lack of clarity regarding inclusion/exclusion criteria for controls and if reported criteria were applied uniformly across groups.<br><br>10. Gut microbiota composition was not measured more than once over time.<br><br>11. Do not clearly report using a validated and consistent tool for psychiatric disorder diagnosis.<br><br>14. Collected BMI data and excluded psychotropic use but did not measure diet; no adjustment for any covariates.
80	Naseribafrouei et al. (2014)[33]	Yes	No	Yes	No	No	No	No	Yes	No	No	Yes	N/A	N/A	No	Poor	2. No time-period for recruitment mentioned.<br><br>4. Lack of clarity regarding inclusion/exclusion criteria across groups.<br><br>5. No statistical methods with effect size estimates were reported.<br><br>9. Did not clearly report 16S rRNA gene sequencing region<br><br>10. Gut microbiota composition was not measured more than once over time.<br><br>14. Some consideration of potential confounding – collected baseline BMI and psychotropic data. However, did not measure/adjust for diet.
100	Nguyen et al. (2019)[46]	Yes	No	Yes	Yes	No	No	No	Yes	Yes	No	No	N/A	N/A	No	Fair	2. No time-period for recruitment mentioned.<br><br>5. No statistical methods with effect size estimates were reported.<br><br>10. Gut microbiota composition was not measured more than once over time.<br><br>11. Appeared to use different validated diagnostic tools across groups.<br><br>14. Some consideration of potential confounding but did not measure/adjust for diet.

Ref	Author (Year)	Q1	Q2	Q3	Q4	Q5	Q6	Q7	Q8	Q9	Q10	Q11	Q12	Q13	Q14	Quality Rating	Notes
107	Nguyen et al. (2021)[52]	Yes	No	Yes	Yes	No	No	No	Yes	Yes	No	No	N/A	N/A	No	Fair	2. No time-period for recruitment mentioned.<br><br>5. No statistical methods with effect size estimates were reported.<br><br>10. Gut microbiota composition was not measured more than once over time.<br><br>11. Appeared to use different validated diagnostic tools across groups.<br><br>14. Some consideration of potential confounding but did not measure/adjust for diet.
101	Pan et al. (2020)[47]	Yes	No	Yes	No	Yes	No	No	Yes	Yes	Yes	No	N/A	N/A	No	Poor	2. Lack of clarity regarding study population; No time-period for recruitment mentioned.<br><br>4. Inclusion/exclusion criteria not consistent across groups.<br><br>11. Do not clearly report using a validated and consistent tool for psychiatric disorder diagnosis.<br><br>14. Some consideration of potential confounding but did not measure/adjust for diet.
81	Rong et al. (2019)[34]	Yes	Yes	Yes	Yes	Yes	No	No	Yes	No	No	No	N/A	N/A	No	Fair	9. Did not clearly report stool sample collection or transport methods, or mapping database<br><br>10. Gut microbiota composition was not measured more than once over time.<br><br>11. Used a validated diagnostic tool, however unclear if this was applied uniformly across groups.<br><br>14. Some consideration of potential confounding but did not measure/adjust for diet.
102	Shen et al. (2018)[48]	Yes	No	Yes	No	Yes	No	No	Yes	Yes	No	No	N/A	N/A	No	Poor	2. Lack of clarity regarding study population; No time-period for recruitment mentioned.<br><br>4. Lack of clarity regarding consistency of inclusion/exclusion criteria across groups; cannot determine if cases and controls were recruited from same population.<br><br>10. Gut microbiota composition was not measured more than once over time.<br><br>11. Do not clearly report using a validated and consistent tool for psychiatric disorder diagnosis.<br><br>14. Measured baseline BMI but did not measure/adjust for diet or psychotropic use.

Ref	Author (Year)	Q1	Q2	Q3	Q4	Q5	Q6	Q7	Q8	Q9	Q10	Q11	Q12	Q13	Q14	Quality Rating	Notes
89	Shen et al. (2021)	Yes	No	Yes	Yes	No	No	No	Yes	Yes	Yes	Yes	N/A	N/A	No	Good	2. Lack of clarity regarding study population; No time-period for recruitment mentioned.<br><br>5. Statistical methods with effect size estimates were reported, however not comparing cases to controls.<br><br>14. Some consideration of potential confounding – collected BMI data and excluded obvious dietary preferences and psychotropic use; did not collect specific dietary information or adjust for any of these variables.
82	Stevens et al. (2020)[35]	Yes	No	Yes	No	Yes	No	No	Yes	Yes	No	No	N/A	N/A	No	Poor	2. Lack of clarity regarding study population; No time-period for recruitment mentioned.<br><br>4. Lack of clarity regarding inclusion/exclusion criteria and if these were applied uniformly across groups.<br><br>10. Gut microbiota composition was not measured more than once over time.<br><br>11. Do not clearly report using a validated and consistent tool for psychiatric disorder diagnosis.<br><br>14. Some consideration of antidepressant use. Did not measure/adjust for BMI, diet, or other psychotropic use.
103	Xu et al. (2020)[49]	Yes	No	Yes	Yes	No	No	No	Yes	Yes	No	No	N/A	N/A	No	Poor	2. Lack of clarity regarding study population; No time-period for recruitment mentioned.<br><br>5. No statistical methods with effect size estimates were reported.<br><br>10. Gut microbiota composition was not measured more than once over time.<br><br>11. Do not clearly report using a validated and consistent tool for psychiatric disorder diagnosis.<br><br>14. Collected diet and BMI baseline data, however did not measure/adjust for psychotropic use.
86	Yang et al. (2020)[38]	Yes	No	Yes	Yes	Yes	No	No	Yes	No	No	Yes	N/A	N/A	Yes	Good	2. Lack of clarity regarding study population; No time-period for recruitment mentioned.<br><br>9. Did not clearly report stool sample collection, transport, or storage methods.<br><br>10. Gut microbiota composition was not measured more than once over time.
90	Ye et al. (2021)	Yes	Yes	Yes	Yes	Yes	No	No	Yes	Yes	No	No	N/A	N/A	No	Good	10. Gut microbiota composition was not measured more than once over time.<br><br>11. Do not clearly report using a validated and consistent tool for psychiatric disorder diagnosis.<br><br>14. Collected BMI data and excluded psychotropic use but did not measure diet; no adjustment for any covariates.

Ref	Author (Year)	Q1	Q2	Q3	Q4	Q5	Q6	Q7	Q8	Q9	Q10	Q11	Q12	Q13	Q14	Quality Rating	Notes
111	Yuan et al. (2021)	Yes	No	Yes	Yes	Yes	No	No	Yes	Yes	Yes	No	N/A	N/A	No	Good	2. Lack of clarity regarding study population<br>11. Used a validated diagnostic tool, however unclear if this was applied uniformly across groups.<br>14. Collected BMI data and excluded psychotropic use, however did not collect dietary data; adjusted alpha-diversity, however did not adjust for diet.
104	Zhang et al. (2020)[50]	Yes	Yes	Yes	No	Yes	No	No	Yes	No	No	No	N/A	N/A	No	Poor	4. Lack of clarity regarding inclusion/exclusion criteria and if these were applied uniformly across groups.<br>9. Did not clearly report 16S rRNA gene sequencing region, or mapping database<br>10. Gut microbiota composition was not measured more than once over time.<br>11. Used a validated diagnostic tool, however unclear if this was applied uniformly across groups.<br>14. Some consideration of BMI and psychotropic data however did not measure/adjust for diet.
91	Zhang et al. (2021)	Yes	Yes	Yes	Yes	Yes	No	No	Yes	No	No	No	N/A	N/A	No	Fair	9. Did not clearly report DNA extraction method or mapping database.<br>10. Gut microbiota composition was not measured more than once over time.<br>11. Do not clearly report using a validated and consistent tool for psychiatric disorder diagnosis.<br>14. Collected BMI data and excluded psychotropic use; did not adjust for diet or BMI.
84	Zheng et al. (2016)[9]	Yes	No	Yes	No	No	No	No	Yes	Yes	No	No	N/A	N/A	No	Poor	2. No time-period for recruitment reported.<br>4. Lack of clarity regarding inclusion/exclusion criteria and if these were applied uniformly across groups.<br>5. No statistical methods with effect size estimates were reported.<br>10. Gut microbiota composition was not measured more than once over time.<br>11. Used a validated diagnostic tool, however unclear if this was applied uniformly across groups.<br>14. Some consideration of BMI and psychotropic use however did not measure/adjust for diet.

Ref	Author (Year)	Q1	Q2	Q3	Q4	Q5	Q6	Q7	Q8	Q9	Q10	Q11	Q12	Q13	Q14	Quality Rating	Notes
105	Zheng et al. (2019)[8]	Yes	No	Yes	No	Yes	No	No	Yes	Yes	No	No	N/A	N/A	No	Poor	2. Lack of clarity regarding study population; No time-period for recruitment mentioned.<br><br>4. Lack of clarity regarding inclusion/exclusion criteria and if these were applied uniformly across groups.<br><br>10. Gut microbiota composition was not measured more than once over time.<br><br>11. Do not clearly report using a validated and consistent tool for psychiatric disorder diagnosis, or consistency of tool across groups.<br><br>14. Some consideration of BMI and psychotropic use however did not measure/adjust for diet.
83	Zheng et al. (2020)[36]	Yes	No	Yes	Yes	Yes	No	No	Yes	No	No	No	N/A	N/A	No	Poor	2. Lack of clarity regarding study population; No time-period for recruitment mentioned.<br><br>9. Did not clearly report stool sample collection, transport, or storage methods<br><br>10. Gut microbiota composition was not measured more than once over time.<br><br>11. Used a validated diagnostic tool, however unclear if this was applied uniformly across groups.<br><br>14. Some consideration of BMI and psychotropic use however did not measure/adjust for diet.
92	Zheng et al. (2021)	Yes	No	Yes	No	Yes	No	No	Yes	Yes	No	No	N/A	N/A	No	Poor	2. Lack of clarity regarding study population.<br><br>4. Lack of clarity regarding inclusion/exclusion criteria and if these were applied uniformly across groups.<br><br>10. Gut microbiota composition was not measured more than once over time.<br><br>11. Do not clearly report using a validated and consistent tool for psychiatric disorder diagnosis, or consistency of tool across groups.<br><br>14. Excluded psychotropic use and changing dietary patterns; did not collect specific dietary data or BMI and did not adjust for these variables.
106	Zhu et al. (2020)[51]	Yes	Yes	Yes	Yes	No	No	No	Yes	No	No	Yes	N/A	N/A	Yes	Good	5. No statistical methods with effect size estimates were reported.<br><br>9. Did not clearly report stool sample collection, transport, storage, or DNA extraction methods<br><br>10. Gut microbiota composition was not measured more than once over time.

Ref	Author (Year)	Q1	Q2	Q3	Q4	Q5	Q6	Q7	Q8	Q9	Q10	Q11	Q12	Q13	Q14	Quality Rating	Notes
112	Zhu et al. (2021)	Yes	Yes	Yes	Yes	Yes	No	No	Yes	No	No	Yes	N/A	N/A	No	Fair	9. Did not clearly report stool sample transport, bioinformatics methods, mapping database, or clearly specify if they used OTUs or ASVs.<br>10. Gut microbiota composition was not measured more than once over time.<br>14. Collected BMI data and excluded psychotropic use and dietary change; did not collect specific dietary data or adjust for diet or BMI.

Quality assessment criteria:

	Question	Guidance
**Q1**	Was the research question or objective in this paper clearly stated?	*Did the authors describe their goal in conducting this research? Is it easy to understand what they were looking to find? This issue is important for any scientific paper of any type. Higher quality scientific research explicitly defines a research question.*
**Q2**	Was the study population clearly specified and defined?	*Did the authors describe the group of people from which the study participants were selected or recruited, using demographics, location, and time-period? If you were to conduct this study again, would you know who to recruit, from where, and from what time-period? Is the cohort population free of the outcomes of interest at the time they were recruited? In cohort studies, it is crucial that the population at baseline is free of the outcome of interest.*
**Q3**	Was the participation rate of eligible persons at least 50%?	*If fewer than 50% of eligible persons participated in the study, then there is concern that the study population does not adequately represent the target population. This increases the risk of bias.*
**Q4**	Were all the subjects selected or recruited from the same or similar populations (including the same time-period)? Were inclusion and exclusion criteria for being in the study prespecified and applied uniformly to all participants?	*Were the inclusion and exclusion criteria developed prior to recruitment or selection of the study population? Were the same underlying criteria used for all the subjects involved?*
**Q5**	Was a sample size justification, power description, or variance and effect estimates provided?	*Did the authors present their reasons for selecting or recruiting the number of people included or analyzed? Do they note or discuss the statistical power of the study? This question is about whether or not the study had enough participants to detect an association if one truly existed.*<br><br>*A paragraph in the methods section of the article may explain the sample size needed to detect a hypothesized difference in outcomes. You may also find a discussion of power in the discussion section. Sometimes estimates of variance and/or estimates of effect size are given, instead of sample size calculations. In any of these cases, the answer would be "yes."*<br>*<u>Author's note</u>: Linear discriminate analysis effect size (LEfSe) was considered adequate.*<br><br>*However, observational cohort studies often do not report anything about power or sample sizes because the analyses are exploratory in nature. In this case, the answer would be "no." This is not a "fatal flaw." It just may indicate that attention was not paid to whether the study was sufficiently sized to answer a prespecified question–i.e., it may have been an exploratory, hypothesis-generating study.*
**Q6**	For the analyses in this paper, were the exposure(s) of interest measured prior to the outcome(s) being measured?	*Per guidelines, cross-sectional studies analyses are awarded a "No" for this criterion. Cross-sectional studies (or cross-sectional analyses of cohort-study data), where the exposures and outcomes are measured during the same timeframe, provide weaker evidence than regular cohort studies regarding a potential causal relationship between exposures and outcomes.*
**Q7**	Was the timeframe sufficient so that one could reasonably expect to see an association between exposure and outcome if it existed?	*Per guidelines, cross-sectional studies analyses are awarded a "No" for this criterion. Cross-sectional analyses allow no time to see an effect, since the exposures and outcomes are assessed at the same time.*
**Q8**	For exposures that can vary in amount or level, did the study examine different levels of the exposure as related to the outcome (e.g., categories of exposure, or exposure measured as continuous variable)?	*If the exposure can be defined as a range, were multiple categories of that exposure assessed? Sometimes discrete categories of exposure are not used, but instead exposures are measured as continuous variables.*<br>*<u>Author's note</u>: for this review, the exposure is gut microbiota composition; thus, studies were awarded a "Yes" for this criterion if they used multiple metrics or statistical techniques to measure gut microbiota composition.*
**Q9**	Were the exposure measures (independent variables) clearly defined, valid, reliable, and implemented consistently across all study participants?	*Were the exposure measures defined in detail? Were the tools or methods used to measure exposure accurate and reliable–for example, have they been validated or are they objective? This issue is important as it influences confidence in the reported exposures.*

		Author's note: for this review, the exposure is gut microbiota composition; thus, studies were awarded a "Yes" if they adequately defined all their methodologies for measuring gut microbiota composition and used validated and/or reliable tools.
**Q10**	Was the exposure(s) assessed more than once over time?	*Was the exposure for each person measured more than once during the course of the study period? Multiple measurements with the same result increase our confidence that the exposure status was correctly classified.*<br>*Author's note: for this review the exposure is gut microbiota composition.*
**Q11**	Were the outcome measures (dependent variables) clearly defined, valid, reliable, and implemented consistently across all study participants?	*Were the outcomes defined in detail? Were the tools or methods for measuring outcomes accurate and reliable–for example, have they been validated or are they objective? This issue is important because it influences confidence in the validity of study results. Also important is whether the outcomes were assessed in the same manner within groups and between groups.*<br>*Author's note: for this review, the outcome is a diagnosis of MDD, BD or SZ; thus, studies were awarded a "Yes" if they used a validated tool for diagnoses, and this tool was used in the same manner between groups.*
**Q12**	Were the outcome assessors blinded to the exposure status of participants?	*Blinding means that outcome assessors did not know whether the participant was exposed or unexposed. It is also sometimes called "masking." The objective is to look for evidence in the article that the person(s) assessing the outcome(s) for the is masked to the exposure status of the participant.*<br>*Author's note: This criterion was considered "Not applicable" (N/A) as study assessors were not aware of the exposure status (gut microbiota composition) of participants when assessing the outcome (psychiatric diagnosis).*
**Q13**	Was loss to follow-up after baseline 20% or less?	*Higher overall follow-up rates are always better than lower follow-up rates, even though higher rates are expected in shorter studies, whereas lower overall follow-up rates are often seen in studies of longer duration. Usually, an acceptable overall follow-up rate is considered 80 percent or more of participants whose exposures were measured at baseline.*<br>*Author's note: This criterion was considered "Not applicable" (N/A) for cross-sectional studies.*
**Q14**	Were key potential confounding variables measured and adjusted statistically for their impact on the relationship between exposure(s) and outcome(s)?	*Were key potential confounding variables measured and adjusted for, such as by statistical adjustment for baseline differences? Logistic regression or other regression methods are often used to account for the influence of variables not of interest.*<br><br>*This is a key issue in cohort studies because statistical analyses need to control for potential confounders, in contrast to an RCT, where the randomization process controls for potential confounders. All key factors that may be associated both with the exposure of interest and the outcome–that are not of interest to the research question–should be controlled for in the analyses.*<br><br>*Author's note: Key confounding variables identified by authors included body mass index (BMI), diet, anti-depressants, and similar psychotropic medication.*

Key. N/A = not applicable; No = study does not satisfactorily meet question criteria; Yes = study satisfactorily meets question criteria; *Notes.* MDD, Major Depressive Disorder; BD, Bipolar Disorder; SZ, Schizophrenia; BMI, Body Mass Index.
