# Supplementary material for: A systematic review of gut microbiota composition in observational studies of major depressive disorder, bipolar disorder and schizophrenia
Source: Mol Psychiatry. 2022 Feb 22;27(4):1920–35. doi: 10.1038/s41380-022-01456-3 (PMC9126816; doi:10.1038/s41380-022-01456-3)

Supplementary Figures

Figure S1. Testing of gut microbiota alpha-diversity across studies of MDD, BD and SZ. α-diversity metrics performed across comparison groups (N=39) by type of metric (i.e., composite index, richness, phylogenetic diversity, evenness) and disorder (MDD, BD, SZ). A purple square indicates lower α-diversity; orange indicates higher α-diversity; grey indicates no reported difference in α-diversity; and white indicates where a metric was not measured. MDD, major depressive disorder; BD, bipolar disorder; SZ, schizophrenia; ACE, abundance-based coverage estimator; TOS, total observed species; SOBS, number of observed species; ICE, incidence-based coverage estimator; MGS, whole-genome metagenomic sequencing; PD, phylogenetic diversity. ** Against the study name denotes MGS, all others used 16S.

Figure S2. Testing of gut microbiota beta-diversity across studies of MDD, BD and SZ. β-diversity metrics performed across comparison groups (N=34) by disorder (MDD, BD, SZ), indicated by a circle. A blue circle indicates a difference in β-diversity between groups, yellow indicates no difference in β-diversity between groups; and white reflects studies that only used β-diversity data to inform visual ordination plots rather than statistical analyses. MDD, major depressive disorder; BD, bipolar disorder; SZ, schizophrenia; ACE, abundance-based coverage estimator; TOS, total observed species; SOBS, number of observed species; ICE, incidence-based coverage estimator; MGS, whole-genome metagenomic sequencing; PD, phylogenetic diversity. ** Against the study name denotes MGS, all others used 16S.

Figure S3. Differentially abundant taxa at the phylum, class, order, and family level, in MDD, BD and SZ. Bacterial taxa that were differentially abundant in equal to or greater than 20% of the included studies at each taxonomic level were collated. A purple square indicates a lower abundance in cases, orange indicates higher abundance in cases, white indicates no difference reported, and grey indicates taxa were reported as both increased and decreased in the same study. MDD, major depressive disorder; BD, bipolar disorder; SZ, schizophrenia; 16S, 16S rRNA sequencing; MGS, whole-genome metagenomic sequencing; A-MDD, active major depressive disorder; R-MDD, remitted major depressive disorder; HCs, healthy controls; nBD, non-bipolar disorder relatives; FEP, first-episode; Treat, treated; rSZ, remitted schizophrenia. ** indicates studies that used MGS sequencing.

Figure S4. Differentially abundant taxa at the genus level in MDD, BD and SZ. Bacterial taxa that were differentially abundant in equal to or greater than 20% of the included studies at the genus level were collated. A purple square indicates a lower abundance in cases, orange indicates higher abundance in cases, white indicates no difference reported, and grey indicates taxa were reported as both increased and decreased in the same study. MDD, major depressive disorder; BD, bipolar disorder; SZ, schizophrenia; 16S, 16S rRNA sequencing; MGS, whole-genome metagenomic sequencing; A-MDD, active major depressive disorder; R-MDD, remitted major depressive disorder; HCs, healthy controls; nBD, non-bipolar disorder relatives; FEP, first-episode; Treat, treated; rSZ, remitted schizophrenia. ** indicates studies that used MGS sequencing.

Figure S5. Differentially abundant taxa at the species level in MDD, BD and SZ. Bacterial taxa that were differentially abundant in equal to or greater than 20% of the included studies at the species level were collated. A purple square indicates a lower abundance in cases, orange indicates higher abundance in cases, white indicates no difference reported, and grey indicates taxa were reported as both increased and decreased in the same study. MDD, major depressive disorder; BD, bipolar disorder; SZ, schizophrenia; 16S, 16S rRNA sequencing; MGS, whole-genome metagenomic sequencing; A-MDD, active major depressive disorder; R-MDD, remitted major depressive disorder; HCs, healthy controls; nBD, non-bipolar disorder relatives; FEP, first-episode; Treat, treated; rSZ, remitted schizophrenia.

Figure S1. Testing of gut microbiota alpha-diversity across studies of MDD, BD and SZ.

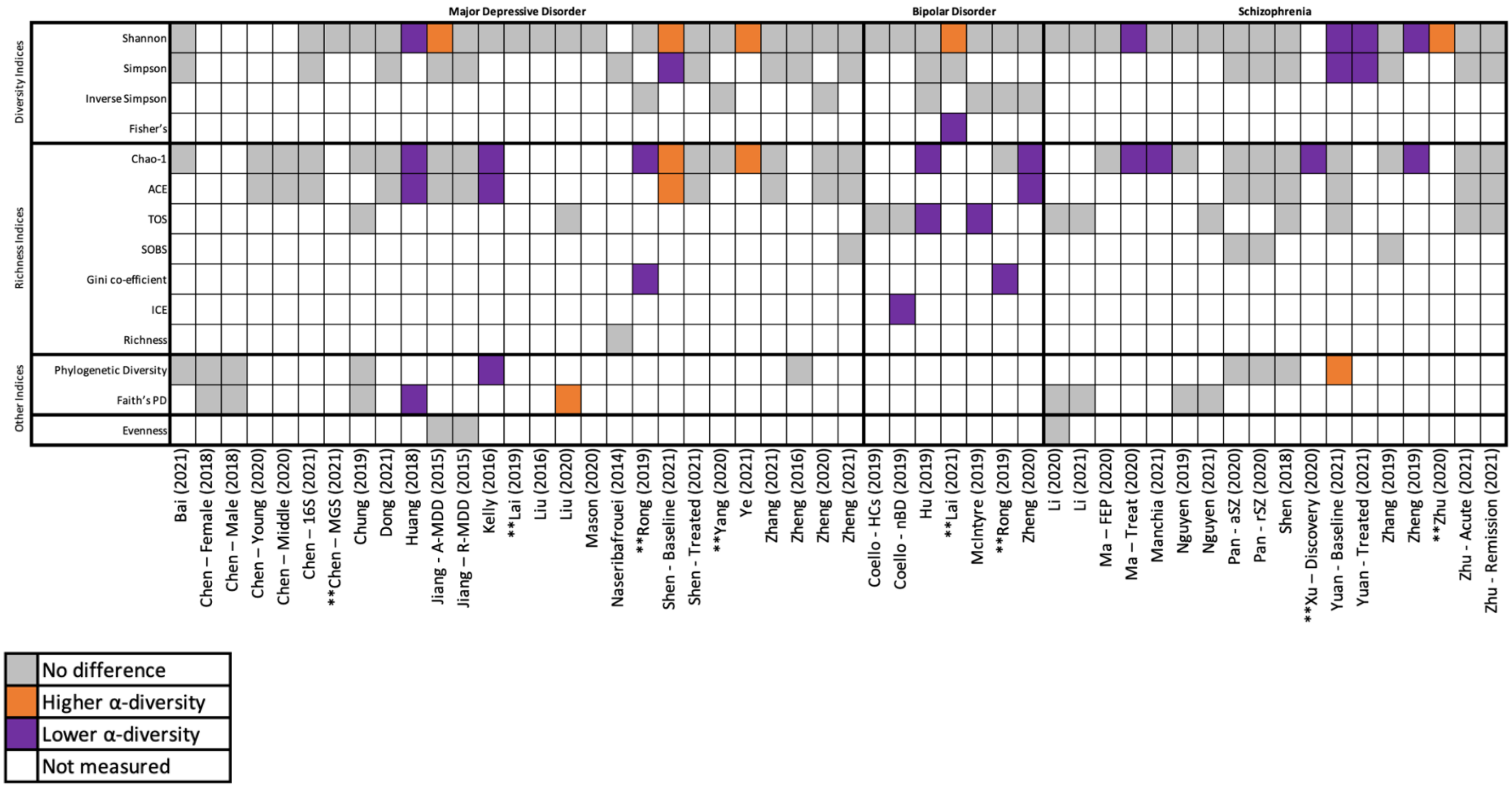

Figure S2. Testing of gut microbiota beta-diversity across studies of MDD, BD and SZ.

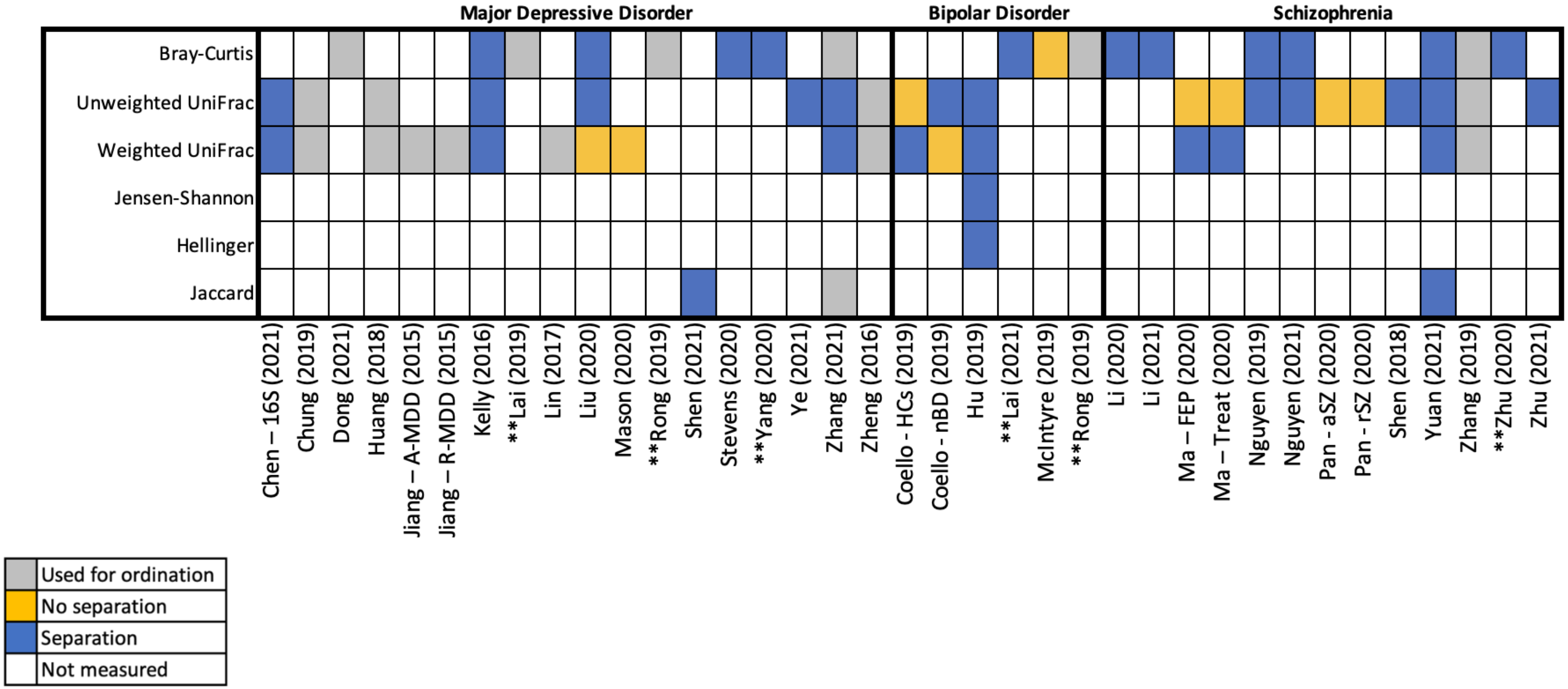

Figure S3. Differentially abundant taxa at the phylum, class, order, and family level, in MDD, BD and SZ.

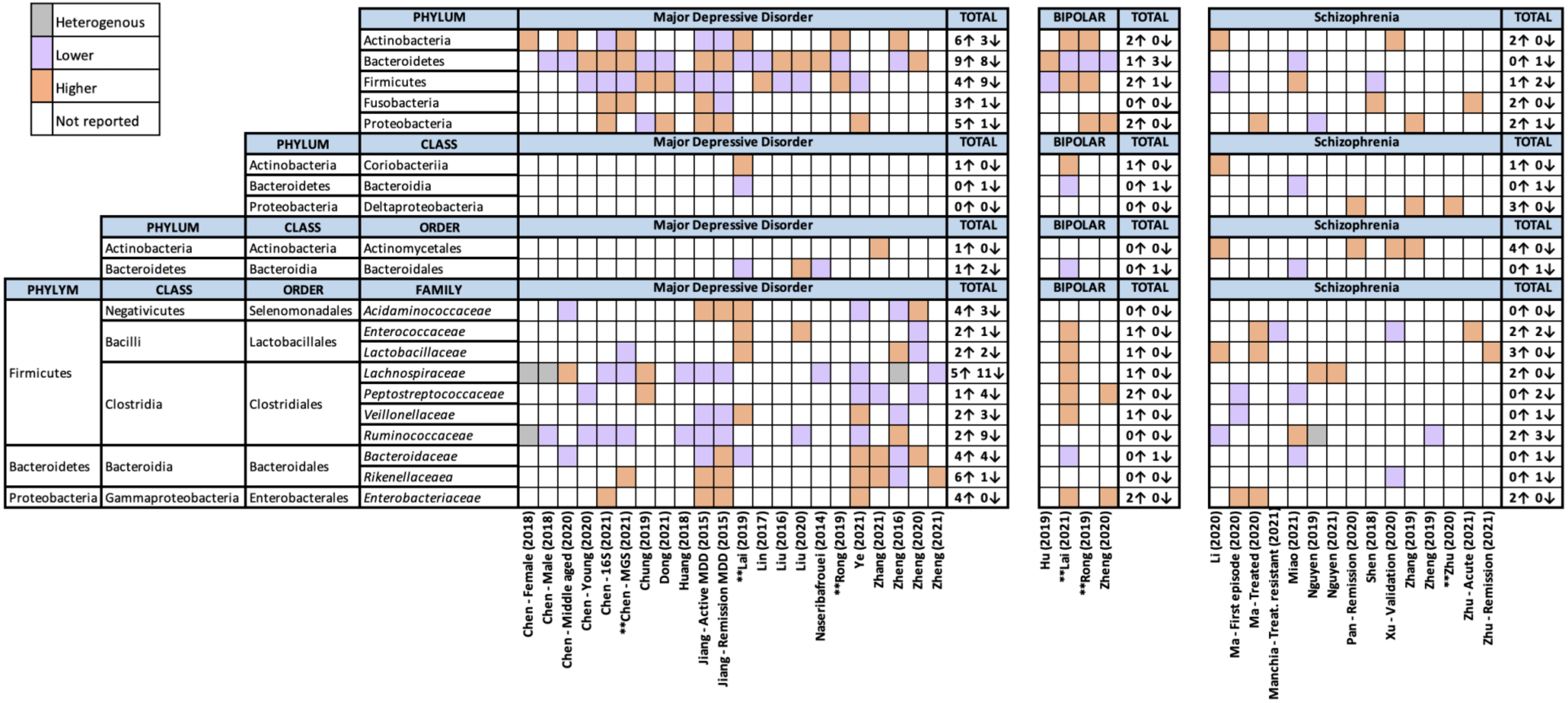

Figure S4. Differentially abundant taxa at the genus level in MDD, BD and SZ.

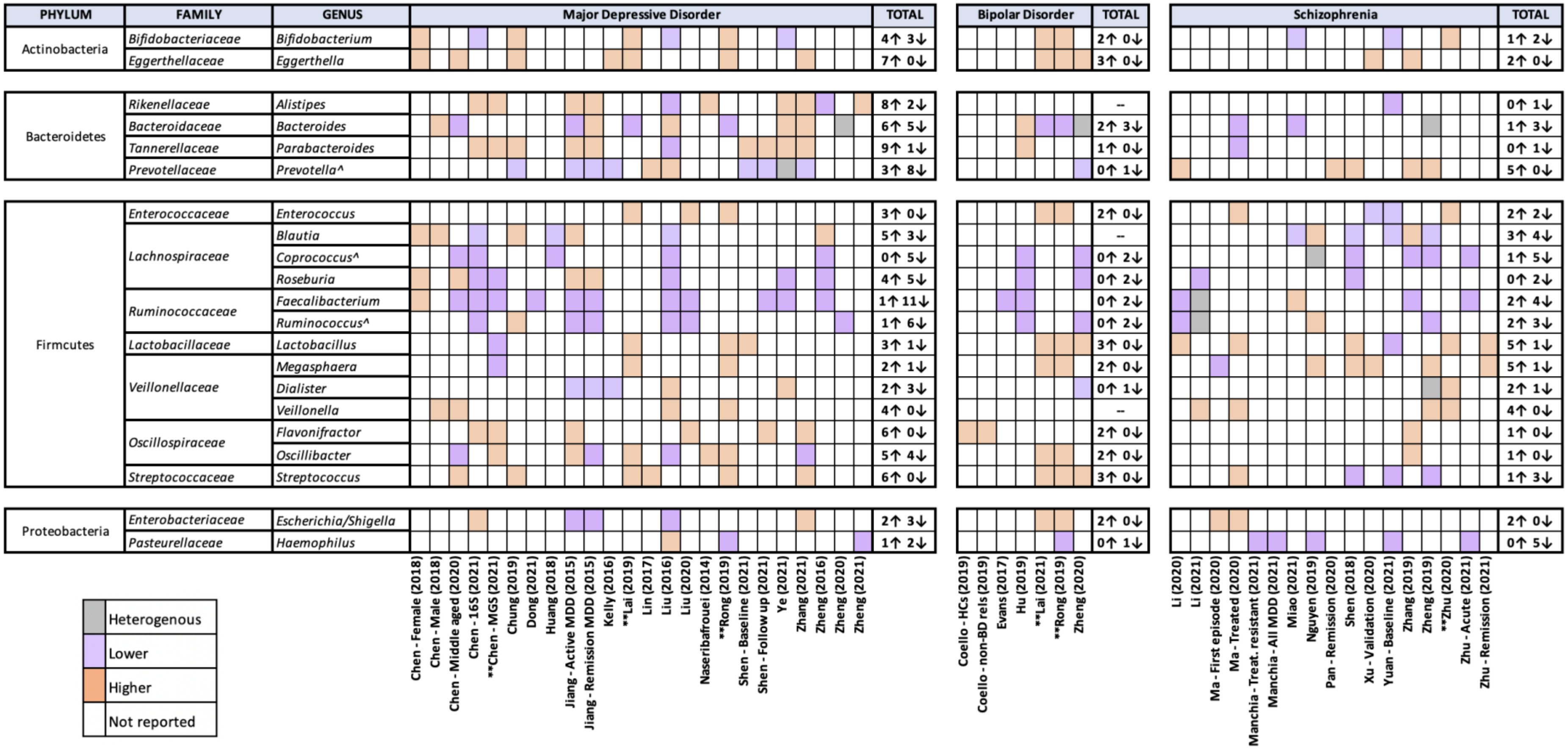

Figure S5. Differentially abundant taxa at the species level in MDD, BD and SZ.

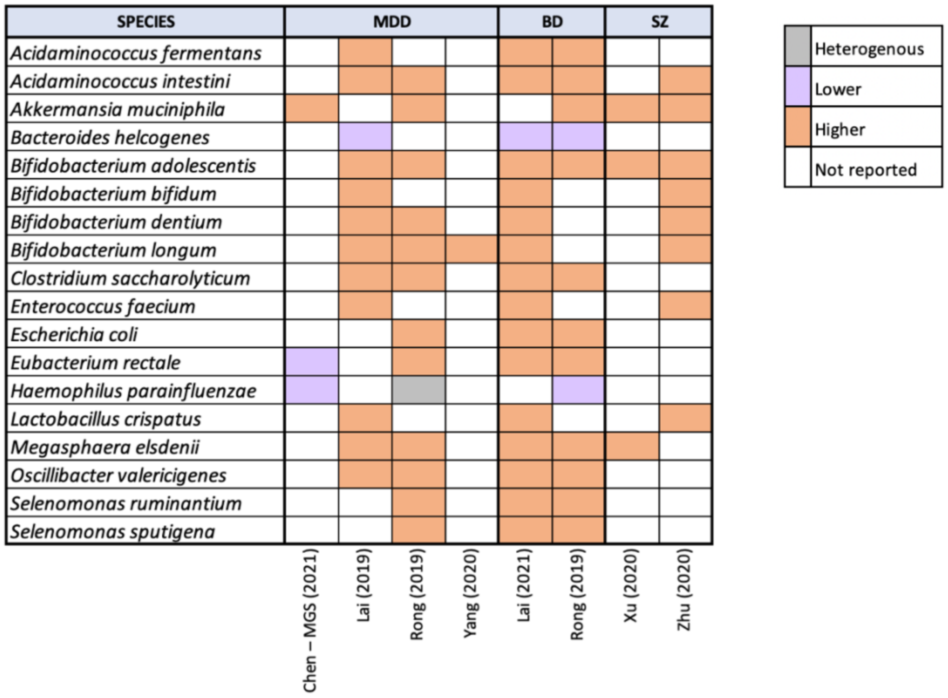

Supplement: Supplementary file 2 — Supplementary Figures S1-S5 [file 41380_2022_1456_MOESM2_ESM.pdf]
